# MBGC: Multiple Bacteria Genome Compressor

**DOI:** 10.1093/gigascience/giab099

**Published:** 2022-01-27

**Authors:** Szymon Grabowski, Tomasz M Kowalski

**Affiliations:** Institute of Applied Computer Science, Lodz University of Technology, ul. Stefanowskiego 18, 90-537 Lodz, Poland; Institute of Applied Computer Science, Lodz University of Technology, ul. Stefanowskiego 18, 90-537 Lodz, Poland

**Keywords:** algorithms, data compression, multiple genome compression, FASTA, pathogens

## Abstract

**Background:**

Genomes within the same species reveal large similarity, exploited by specialized multiple genome compressors. The existing algorithms and tools are however targeted at large, e.g., mammalian, genomes, and their performance on bacteria strains is rather moderate.

**Results:**

In this work, we propose MBGC, a specialized genome compressor making use of specific redundancy of bacterial genomes. Its characteristic features are finding both direct and reverse-complemented LZ-matches, as well as a careful management of a reference buffer in a multi-threaded implementation. Our tool is not only compression efficient but also fast. On a collection of 168,311 bacterial genomes, totalling 587 GB, we achieve a compression ratio of approximately a factor of 1,265 and compression (respectively decompression) speed of ∼1,580 MB/s (respectively 780 MB/s) using 8 hardware threads, on a computer with a 14-core/28-thread CPU and a fast SSD, being almost 3 times more succinct and >6 times faster in the compression than the next best competitor.

## Background

Genome compression is a fairly old research topic, dating back to the mid-1990s [1]. It was soon realized that even sophisticated techniques for compressing a single genome, e.g., [2], cannot offer much higher compression ratios than simple packing of DNA symbols into 2 bits each (see also the recent experimental comparison [3]). The interest of researchers thus shifted into relative compression of a genome given a reference [4–8], typically representing the same species, or compression of a given collection of genomes without an external reference [9–11]. Some of those proposals apply quite advanced techniques (e.g., GDC 2 [11], GeCo3 [12]), while others use rather simple input preprocessing followed by a general-purpose back-end compressor, like 7zip in DELIMINATE [13] or zstd in NAF [14]. For example, GDC 2 uses 2-pass LZ77 matching, and the matches in the latter pass can be built of several matches found in the former pass, to obtain unsurpassed compression ratios on large human genome collections (e.g., the ratio of ∼9,500 on 1,092 human diploid genomes). GeCo3 [12] combines the power of neural networks with specific DNA models, but its compression on a 2–4 GB genomic collection already takes hours. Allowing mismatches (mutations) in matches, thus leveraging a generalized notion of a standard LZ-match, proved successful in MemRGC [8], a relative compressor for a single genome.

The abundance of full genomes available in major repositories, like NCBI or 1KGP, in recent years poses a challenge to compress them efficiently, preferably combining high compression ratios, fast compression and decompression, and reasonable memory requirements. In this work, we focus on the compression of bacterial genomes (without an external reference), for which existing genome collection compressors are not appropriate for algorithmic or technical reasons (e.g., ignoring reverse-complemented matches, slow compression of long DNA sequences interspaced with EOL symbols, lack of N symbol support or constraints concerning the number of sequences in a single FASTA file). We note that there exist also other possible aspects of compressors (or compression-based tools), e.g., random access support [9,15,16] or searching directly in the compressed data, also in an approximate manner [17]. For more references, see the survey [18].

## Analyses

For the experiments we took a large collection of 168,311 bacterial genomes in the FASTA format from the NCBI Pathogen Detection project, and 4 subsets of it that each contained 1,024 genomes and represented a single species (except for a joint subset with *Escherichia coli* and *Shigella* genomes). The proposed MBGC and other compressors were tested on a Linux (Debian) machine equipped with a 14-core Intel Core i9-10940X 3.3 GHz CPU, 128 GB of DDR4-RAM (CL 16, clocked at 2,666MHz), and a fast SSD (ADATA 2 TB M.2 PCIe NVMe XPG SX8200 Pro). MBGC is written in C++ and was compiled with gcc 10.2.1. The disk cache was flushed between runs so as to have raw reads of the input files from the disk. By the compression ratio we mean the ratio between the original input size and the compressed size, e.g., reducing a 500-MB input to 500-kB results in the compression ratio of 1,000. Also, if the ratio improves from 1,000 to 1,500, e.g., owing to changing some parameters of the compressor, we can say that the compression ratio improves by a factor of 1.5 (or by 50%).

For the competitors of MBGC we chose 1 multiple genome compressor (HRCM [7]), 1 more versatile bioinformatics data–oriented compressor (NAF), and a few popular high-quality general-purpose compressors (BSC, 7zip, zstd). Note that NAF makes use of zstd as its back-end compressor. Our selection is based on practical performance of the tools, concerning the compression ratio and (de)compression speed, within reasonable memory requirements. Some well-known multiple genome compressors, namely, GDC 2 [11], iDoComp [6], and memRGC [8], are not used in our (main) experiments, for the reasons explained in the supplementary material (Section 4.2).

As can be seen (Table 1–3), MBGC in the max mode wins easily in the compression ratio on the *E. coli, Listeria monocytogenes*, and *Salmonella enterica* subsets, as well as on the total collection. MBGC with the default settings also usually dominates the rest of the contenders, yet 7zip sometimes beats it by a few percent, owing to its large (4 GB) sliding window; the same feature also makes 7zip the most memory consuming (per worker thread) tool. The only case where 7zip beats MBGC max in the compression ratio is the 1,024-genome *Campylobacter jejuni* collection (Table 1). MBGC also dominates in the compression times, although not always in the decompression times. Both the compression and the decompression speed of our solution are at least on the order of hundreds of MBs per second, partly due to a multi-threaded implementation. The most successful case is arguably the large *S. enterica* collection (Table 2), where the MBGC default slightly exceeds 2 GB/s of the compression speed, achieving the compression ratio of 5,786, which contrasts with the compression ratio of 1,312 from the 11 times slower NAF, and of 696 from the 481 times slower 7zip. The performance of the specialized genome compressor, HRCM, is more or less average, and we refrained from running it on the whole collection because the compression would take several days.

**Table 1:** Compression results—collections of 1,024 genomes

Parameter	HRCM	BSC -p -b2047	7z -md4g	zstd -3 –long = 31	NAF -3 –long = 31	NAF -19 –long = 31	MBGC	
							default	max
*C. jejuni*(1.78 GB)								
ratio	15.0	40.2	^(1)^78.5	31.2	43.0	54.2	^(3)^62.2	^(2)^73.1
ctime (s)	196.3	38.0	1,064.5	^(2)^4.0	^(3)^9.8	136.2	^(1)^3.7	12.4
dtime (s)	27.0	10.6	^(3)^3.0	^(1)^1.5	4.4	4.4	^(2)^2.8	3.7
cmem (GB)	^(2)^1.76	9.00	18.00	1.92	1.90	2.24	^(3)^1.78	^(1)^1.19
dmem (GB)	^(1)^0.28	8.81	1.79	1.76	1.76	1.76	^(3)^1.45	^(2)^1.34
*E. coli*(4.87 GB)								
ratio	158.2	166.9	377.5	357.8	495.2	^(3)^530.0	^(2)^1,406.1	^(1)^1,452.3
ctime (s)	308.3	127.4	2,228.7	^(3)^10.0	20.8	57.5	^(1)^3.0	^(2)^7.3
dtime (s)	28.9	42.4	5.6	^(3)^2.9	12.6	11.9	^(1)^1.5	^(1)^1.5
cmem (GB)	^(2)^1.88	24.04	45.98	2.31	^(3)^2.30	2.65	2.55	^(1)^1.22
dmem (GB)	^(1)^0.66	21.48	4.82	2.15	2.21	2.21	^(2)^1.17	^(2)^1.17
*L. monocytogenes* (3.09 GB)								
ratio	39.3	70.4	^(2)^268.8	82.9	131.1	163.3	^(3)^243.0	^(1)^274.9
ctime (s)	225.5	123.5	1,838.1	^(2)^4.5	13.8	102.8	^(1)^3.2	^(3)^9.3
dtime (s)	26.0	36.7	4.0	^(1)^2.1	7.6	7.6	^(1)^2.1	^(3)^2.4
cmem (GB)	^(2)^1.79	15.29	31.82	2.31	2.30	2.63	^(3)^2.27	^(1)^1.25
dmem (GB)	^(1)^0.30	15.30	3.07	2.15	2.15	2.15	^(3)^1.55	^(2)^1.41
*S. enterica*(5.2 GB)								
ratio	268.4	130.0	472.4	427.9	547.5	^(3)^607.1	^(2)^1,329.8	^(1)^1,356.8
ctime (s)	308.2	130.6	2,350.3	^(2)^6.1	20.4	36.1	^(1)^3.1	^(3)^7.3
dtime (s)	28.2	43.0	5.9	^(3)^3.0	12.8	12.9	^(1)^1.7	^(2)^1.9
cmem (GB)	^(2)^1.91	25.67	49.45	2.31	^(3)^2.30	2.64	2.62	^(1)^1.22
dmem (GB)	^(1)^0.48	25.68	5.15	2.15	2.17	2.17	^(3)^1.27	^(2)^1.26

Ratio indicates the ratio of the input to the output size; cmem and dmem indicate compression and decompression memory usage, respectively; ctime and dtime indicate compression and decompression time. The best 3 results in each row are marked with a superscript number in parentheses. HRCM is single-threaded (except for the latter phase where it invokes 7zip), BSC uses 12 threads, 7zip (up to) 6 threads, zstd 14 threads, and MBGC 8 threads.

**Table 2:** Compression results—large species collections

Parameter	BSC -p -b2047	7z -md4g	zstd -3 –long = 31	NAF -3 –long = 31	NAF -19 –long = 31	MBGC	
						default	max
*C. jejuni* (55,627 genomes, totalling 98.38 GB)							
ratio	69.7	164.9	74.9	137.1	^(3)^176.6	^(2)^412.5	^(1)^450.6
ctime (s)	969.2	2,2881.0	^(2)^211.9	489.5	2,570.7	^(1)^92.7	^(3)^400.8
dtime (s)	241.3	129.3	^(3)^127.3	280.3	250.9	^(1)^78.5	^(2)^102.3
cmem (GB)	128.84	122.54	^(2)^2.32	^(1)^2.31	^(3)^2.66	8.78	7.22
dmem (GB)	129.21	34.59	^(1)^2.15	^(3)^2.70	^(2)^2.69	5.62	5.02
*E. coli* (22,523 genomes, totalling 114.67 GB)							
ratio	93.1	342.7	242.8	^(3)^460.9	458.5	^(2)^1,747.4	^(1)^2,051.6
ctime (s)	1195.1	30,086.0	^(2)^165.5	446.3	1,313.5	^(1)^65.0	^(3)^216.1
dtime (s)	291.6	^(3)^160.2	164.7	296.0	288.7	^(2)^83.3	^(1)^78.4
cmem (GB)	128.84	122.48	^(1)^2.31	^(1)^2.31	^(3)^2.66	9.66	10.87
dmem (GB)	129.17	34.48	^(1)^2.15	^(3)^3.10	^(3)^3.10	3.25	^(2)^2.66
*L. monocytogenes* (36,448 genomes, totalling 112.00 GB)							
ratio	90.2	^(3)^328.2	137.3	274.9	323.9	^(2)^1,086.9	^(1)^1,160.2
ctime (s)	1,166.4	28,065.0	^(2)^162.7	450.1	1,805.7	^(1)^68.0	^(3)^263.8
dtime (s)	287.6	^(3)^162.0	184.5	286.4	279.0	^(1)^84.2	^(2)^97.2
cmem (GB)	128.84	122.48	^(2)^2.32	^(1)^2.31	^(3)^2.66	7.85	7.03
dmem (GB)	129.13	34.48	^(1)^2.15	^(2)^2.39	^(2)^2.39	3.82	3.30
*S. enterica* (53,713 genomes, totalling 262.21 GB)							
ratio	156.7	695.5	606.0	1,205.7	^(3)^1,312.0	^(2)^5,786.0	^(1)^5,881.1
ctime (s)	2,655.8	61,182.0	^(2)^290.2	909.9	1,423.9	^(1)^127.3	^(3)^342.1
dtime (s)	618.0	440.4	^(3)^396.5	659.9	662.3	^(2)^270.3	^(1)^262.0
cmem (GB)	128.84	122.46	^(1)^2.31	^(1)^2.31	^(3)^2.66	11.31	10.48
dmem (GB)	129.06	34.43	^(1)^2.15	2.80	2.79	^(3)^2.51	^(2)^2.36

Ratio indicates the ratio of the input to the output size; cmem and dmem indicate compression and decompression memory usage, respectively; ctime and dtime indicate compression and decompression time. The best 3 results in each row are marked with a superscript number in parentheses. BSC uses 12 threads, 7zip (up to) 6 threads, zstd 14 threads, and MBGC 8 threads.

**Table 3:** Compression results—mixed species collections

Parameter	BSC -p -b2047	7z -md = 4g	zstd -3 –long = 31	NAF -3 –long = 31	NAF -19 –long = 31	MBGC	
						default	max
168,311 genomes (587.26 GB)							
ratio	105.3	354.5	193.6	369.0	^(3)^434.0	^(2)^1,266.6	^(1)^1,411.4
ctime	5,824.0	140,902.0	^(2)^846.1	2,287.3	7,100.0	^(1)^370.9	^(3)^1,271.6
dtime (s)	1,379.7	^(3)^880.8	970.2	1,506.2	1,499.7	^(1)^749.2	^(2)^757.2
cmem (GB)	128.84	122.54	^(1)^2.31	^(1)^2.31	^(3)^2.66	23.06	15.93
dmem (GB)	129.22	34.59	^(1)^2.15	^(2)^4.51	^(3)^4.53	8.70	10.73
4 × 1,024 genomes (14.94 GB)							
ratio	88.2	^(2)^239.2	124.5	177.1	214.6	^(3)^220.8	^(1)^351.5
ctime (s)	189.4	4,124.0	^(2)^24.4	62.6	334.6	^(1)^13.4	^(3)^36.5
dtime (s)	56.7	15.5	^(3)^9.9	37.4	37.3	^(2)^8.9	^(1)^8.6
cmem (GB)	73.79	122.48	^(2)^2.31	^(2)^2.31	2.65	4.69	^(1)^2.22
dmem (GB)	73.82	14.83	^(1)^2.15	^(2)^2.24	^(2)^2.24	3.24	2.83

Ratio indicates the ratio of the input to the output size; cmem and dmem indicate compression and decompression memory usage, respectively; ctime and dtime indicate compression and decompression time. The best 3 results in each row are marked with a superscript number in parentheses. BSC uses 12 threads, 7zip (up to) 6 threads, zstd 14 threads, and MBGC 8 threads.

MBGC in the default mode is more than an order of magnitude faster than NAF -9 in compression and at least twice as fast in the decompression. The gap in the compression ratio between MBGC and NAF (in its stronger mode) increases with larger collections, reaching a factor of 4.4 for the whole *S. enterica*, and is ∼2.9 for the collection of all genomes. On the other hand, NAF is more memory-frugal, which may matter if the experiments are run, e.g., on a standard laptop (MBGC default needs 23 GB of RAM to compress the whole collection). Zstd (only experiments of its default mode -3 are presented) is significantly faster than NAF with the same settings but offers a noticably worse compression, sometimes even twice as bad. Let us also comment on the performance of the stronger MGBC mode. We note that MBGC max obtains a compression ratio that is a few percent better than that for the default mode (with the largest gain for the 4 × 1024 collection), but it is 2.5–4 times slower in the compression. The compression and decompression memory usage remains reasonable (although not as good as for NAF and zstd), and the max mode even tends to be more frugal than the default mode.

The results of 7zip were obtained by limiting thread usage to 6 (to avoid excessive memory usage during the compression). For smaller collections it is slower by more than an order of magnitude in compression (but faster in the decompression) than NAF (-9) while its compression ratio is usually comparable to NAF’s (although it varies for individual cases). BSC, which is a strong general-purpose compressor based on the Burrows–Wheeler transform (BWT), performed relatively poorly on larger pathogen collections, obtaining a compression ratio a few times smaller than other competitors. Moreover, it is the slowest in the decompression and is also quite memory-hungry (particularly striking in the decompression) in our experiments, which can be explained by running 12 blocks of (up to) 2 GB each in parallel.

We point out that for the purpose of testing general-purpose tools (zstd, BSC, and 7zip) we applied a unified strategy to avoid hampering their compression in any way. First, the end-of-line (EOL) symbols were removed from the DNA strings in the input files prior to the experiment. As a side note, we point out that MBGC accepts EOL symbols in the input but does not preserve them in the decompressed output (it uses no EOLs in those strings by default or can insert EOLs in regular gaps in DNA strings, as specified by the user). Second, zstd and BSC work with a single file input (and output) and for this reason we combined the input into a TAR archive (the preprocessing time for the compression process and the postprocessing time for the decompression process were not included).

Preliminary experiments (with 1,024 genome collections) show that on the original data (i.e., with EOLs preserved) 7zip needs ∼40% more time to compress and its compression ratio is worse by a factor of 2–3. The respective losses are even greater for zstd (∼3–7 in the compression ratio, and 2 in the compression time with regard to the stronger mode). Such striking differences are however understandable; there are many long LZ-matches in our data, which are broken in “random” positions with the EOL characters.

It may be interesting to check the impact of reverse-complement matches on the MBGC performance. It is significant indeed; according to our preliminary experiments, on *C. jejuni* and *L. monocytogenes* the compression ratio with RC-matches turned off deteriorates roughly by a factor between 1.1 and 1.7 in the default mode.

Throughout all the presented experiments (except for those presented in Fig. 3, in MBGC and “ncbi” scenarios) the input data are in the uncompressed (FASTA) format. Still, MBGC can read gzipped FASTA and we briefly checked how it affects overall performance. The gzipped stream is decompressed with the aid of libdeflate [19], a library for fast whole-buffer Deflate-based decompression (and compression as well, but we use it only for reading). On the individual species collections the compression time gets slightly better (e.g., by even 21% for *S. enterica*), with 12.5% speedup for the whole genome collection (all numbers with respect to the default mode of MBGC). The compression ratio varies a little (due to unpredictable access to genomes with the worker threads), usually <1%.

**Figure 1:**
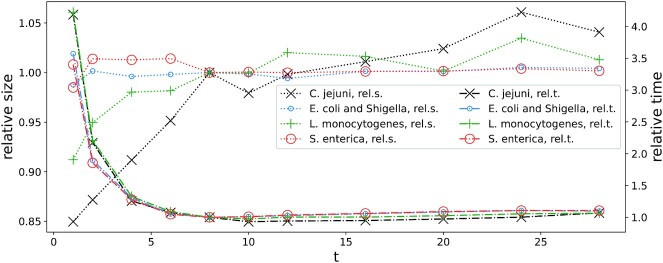
Relative compression ratios and times as a function of the number of threads, with respect to the default mode of MBGC. The left (respectively right) *y*-axis is related to relative compressed ratios (respectively compression times). On both (left and right) *y*-axes smaller is better.

Figure 1 shows the compression ratio and compression speed with varying the number of threads from 1 to 28. The speed does not improve with >6 threads (but perhaps it would with even more efficient disk I/O). The compression ratio is rather unaffected for *E. coli* and *S. enterica*, but using already >1 thread for *C. jejuni* and *L. monocytogenes* yields a few percent compression loss. For *C. jejuni* the gap is as large as ∼15% when the number of threads increases from 1 to (the default) 8. On the other hand, using 8 threads is ∼3–4 times faster than 1 thread in the compression for all 4 datasets, and for this reason we find the compression loss in half of the cases acceptable.

**Figure 2:**
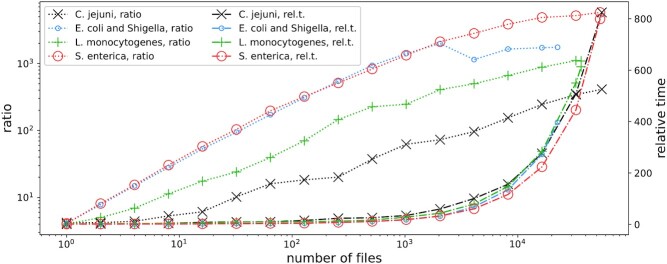
Compression ratios and relative times when the number of input genomes increases, with respect to the default mode of MBGC. The left (respectively right) *y*-axis is related to compressed ratios (respectively relative compression times). On the left (respectively right) *y*-axis greater (respectively smaller) values are better.

Finally, in Fig. 2 we can see how the compression ratio and compression times change when more and more genomes are given as the input. The number of threads was set to 8 (default). As expected, the compression time increases roughly linearly (note the *x*-axis scale), but the compression ratio improves, as for further genomes more similar “pieces” can be found in the already processed collection (or, to be more precise, in the currently used *REF* sequence). The only exception is *E. coli*, where after processing ∼2,000 genomes the compression ratio first deteriorates somewhat and then no longer improves. This can be easily explained by the heterogeneity of this dataset, which comprises both *E. coli* and (closely related to *E. coli*, but different) *Shigella* genomes.

For a separate experiment, we took 2 non-bacterial genome collections, *Saccharomyces cerevisiae* and *Saccharomyces paradoxus* (Table 4). We did not expect MBGC to be competitive here, and indeed, GDC 2 and 7z are superior in the compression ratio but MBGC remains the second fastest (after zstd -3) tool in the compression process while still maintaining a relatively high compression ratio. A better overall choice is, however, GDC 2, with a significantly higher compression ratio and being only slightly slower in the compression than MBGC max on *S. cerevisiae*. On the other hand, the compression speed difference is >6-fold, in favor of MBGC max, in the case of *S. paradoxus*. In decompression, zstd is the fastest, followed by GDC 2 and 7z, and then by NAF and MBGC. BSC and HRCM are more than twice as slow in the decompression than MBGC. Of these two, HRCM is a better pick owing to higher compression ratio and relatively fast compression.

**Table 4:** Compression results on non-bacterial genome collections, *S. cerevisiae* and *S. paradoxus*

Tool	*S. cerevisiae* (39 genomes, 486 MB)					*S. paradoxus* (36 genomes, 429 MB)				
	ratio	ctime (s)	dtime (s)	cmem (GB)	dmem (GB)	ratio	ctime (s)	dtime (s)	cmem (GB)	dmem (GB)
GDC 2	^(1)^109.8	4.12	^(2)^0.57	^(1)^0.52	^(2)^0.14	^(2)^80.7	21.92	^(3)^0.82	^(2)^0.51	^(2)^0.17
HRCM	78.8	7.13	2.85	1.18	^(1)^0.06	52.6	8.30	3.18	1.18	^(1)^0.07
BSC -p -b2047	52.9	10.76	2.65	2.49	2.44	33.8	9.59	2.59	2.21	2.16
7z -md4g	^(2)^100.6	316.38	^(3)^0.73	4.92	0.50	^(1)^83.9	295.95	^(2)^0.69	4.41	0.44
zstd -3 –long=31	45.8	^(1)^0.98	^(1)^0.43	^(2)^0.54	^(3)^0.49	30.5	^(1)^0.87	^(1)^0.40	^(1)^0.49	^(3)^0.43
NAF -3 –long=31	67.0	^(3)^2.80	1.02	^(3)^0.63	^(3)^0.49	43.2	^(3)^2.61	0.92	^(3)^0.57	^(3)^0.43
NAF -19 –long=31	77.0	30.54	1.03	0.97	^(3)^0.49	43.2	33.54	0.93	0.91	^(3)^0.43
MBGC default	87.3	^(2)^1.68	0.82	1.93	0.80	49.1	^(2)^2.08	1.07	1.84	0.73
MBGC max	^(3)^90.6	3.72	1.04	1.50	0.87	^(3)^61.5	3.58	1.24	1.40	0.70

Ratio indicates the ratio of the input to the output size; cmem and dmem indicate compression and decompression memory usage, respectively; ctime and dtime indicate compression and decompression time. The best 3 results in a column are marked with a superscript number in parentheses. HRCM is single-threaded (except for the latter phase where it invokes 7zip),HRCM is single-threaded (except for the latter phase where it invokes 7zip), BSC uses 12 threads, 7zip (up to) 6 threads, zstd 14 threads, and MBGC 8 threads.

In the supplementary material (Tables S1 and S2) we also present compression results for a small collection of human genomes (hg16,..., hg19). Although these kinds of data are not the target of MBGC, our tool performs satisfactorily here as well, with quite competitive compression ratios and speed.

## Potential Implications

Our experiments with MBGC show that the genomes of some bacteria species can be collectively compressed by a factor >1,000, at a (de)compression speed of >1 GB/s (as shown on the total collection of 168k pathogen individuals). This may be an argument for replacing the dominant gzip compression format with a more resource-effective solution in DNA repositories, both for the end user (i.e., faster dissemination of genomic data) and for data resource management (e.g., easier backup). A slightly less obvious but still promising application could be using the proposed format for rapid download. To this end, the genomes selected by a user to download could then be lumped together and compressed by a factor, say, between 10 and 100 (depending on the count and similarity of the datasets of choice), which is likely to offset the cost of the compression process. It is not clear whether and how *caching* compressed groups of genomes downloaded together could improve this process, yet this possibility and resulting trade-offs seem worth exploring.

**Figure 3:**
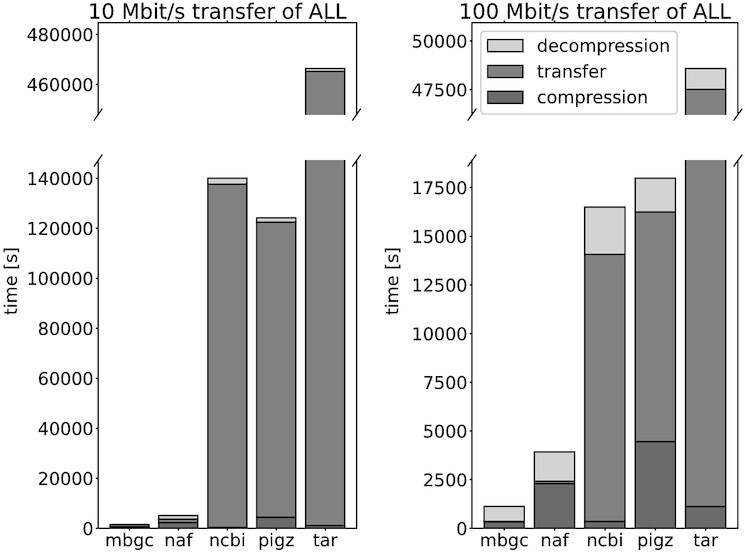
Total times of compressing, transferring, and decompressing a collection of 168,311 genomes. The inputs in the MBGC and “ncbi” scenarios were gzipped FASTA files.

Figure 3 presents a combined measure expressed as the total time to transfer (download) the entire collection of our test genomes. Each bar consists of 3 parts, the compression time (at our test machine), the transfer time (assuming a network connection link of 10 Mbit/s or 100 Mbit/s, on the left and right panel, respectively), and the decompression time (at the same test machine). The compression switches used are as follows: NAF -3 –long=31, pigz -6, mbgc -c 1 (default). The inputs for MBGC are the original gzip files (as provided in NCBI). The input FASTA files for pigz were stripped of EOL symbols prior to the compression. The “tar” bars basically correspond to transmitting raw FASTA files, where the compression phase is data tarring (merging), and the decompression phase is data untarring. The “ncbi” bars correspond to the gzip archives in the NCBI repository, where the compression time comprises only tarring the data (so, it is some lower bound estimation). Clearly, MBGC has a huge edge over the competitors, and only NAF comes relatively close with the faster network connection. Note also that even with a faster connection the gzip-based approaches (bars “ncbi” and “pigz”, which is a multithreaded gzip implementation) are more than an order of magnitude slower than MBGC. The gaps are generally greater with a slower connection, and for the transfer of 10 Mbit/s the advantage of MBGC over NAF is >3-fold and by a factor of almost 100 over “ncbi”. Clearly (cf. also Fig. 2), the gains will be smaller with a smaller amount of data to download at a time, and also the impact of the compression and the decompression times increases with faster networks, making the results generally flatter. Similar figures for species collections of (all and 1,024) genomes are included in the supplementary material (Fig. S1 and Fig S2).

We believe that the ideas behind MBGC can be adapted for a dedicated compressed index for bacterial genomes, allowing for fast pattern counting and reporting. Such an index could handle multi-genome mapping, i.e., mapping sequencing reads against multiple genomes in an efficient way (see, e.g., [18, 20, 21] and references therein). Compressed indexes for repetitive data have been a major research area in the string matching community in the past decade, but few solutions have been tested on a large scale, e.g., hundreds of gigabytes (one exception could be the MuGI index [22] which, e.g., can maintain 1,092 diploid human genomes in <8 GB of space, serving exact pattern queries of length 150 bp in <80 μs on a commodity PC). Perhaps the major obstacle in running industry-scale experiments was construction costs, in both time and space, for many worst-case–oriented indexing data structures. It could be argued that the level of similarity of bacterial genomes allows for relaxing the requirements and focusing on typical, not worst, cases, to obtain practical performance. Although the prospects are not fully clear, it is our opinion that the ideas of MBGC could be adapted to obtain a compressed index for bacterial collections combining high compression ratios, relatively low computational requirements of the construction, and short access times.

## Methods

There is significant redundancy in bacterial genomes that cannot be fully exploited using existing multiple genome compressors. The standard approach of finding repetitions between the currently processed genome and a reference genome (or possibly all previously processed genomes), and encoding them as LZ-phrases of the form (offset, length), is only moderately successful. We found out that many strings repeat as reverse-complements of corresponding strings from other genomes, a phenomenon known, but surprisingly rarely handled earlier (the COMRAD tool being an exception [23]).

It is also beneficial not to limit the reference to 1, or a few, previous genome(s) but to make it possible to find matches occurring almost anywhere earlier. This, however, requires a potentially unbounded memory buffer. We mitigate this problem by building the reference string, i.e., a reservoir for possible matches, in an incremental manner, appending only blocks that are “new enough,” i.e., containing a relatively large fraction of DNA subsequences not seen before. This (general) approach, i.e., building “a dictionary of repeats,” is known in the context of relative genome compression; see, e.g., [9] and [24].

Because the key ideas of our solution, Multiple Bacteria Genome Compressor (MBGC), have already been sketched, now we present the algorithm in detail.

### Basic algorithm

The main stages of MBGC compression are the contig matching process and the back-end compression of matching products. Below we focus on explaining the former, essential stage.

The goal is to compress the sequence of genomes *G*_1_, …, *G_n_* in the FASTA format. The genomes consist of 1 or many contigs (by a contig, throughout the article, we mean a sequence in the FASTA file). At the start the reference string “REF” is initialized with *G*_1_ followed by *rc*(*G*_1_), where *rc*( · ) stands for the reverse complement of the passed string. MBGC also stores a literal buffer, which is initialized with REF (but not its reverse complement). During the compression process, a hash table of fixed size (e.g., 2^25^ slots) is maintained, and pairs of the form (*h*, pos) are inserted to it, where the positions “pos” are taken from REF accessed sparsely, with a stride of 16 symbols, and *h* are hash values of corresponding *k*-mer seeds taken from the sampled positions. A collision on the hash *h* overwrites the previous value associated with it.

In the following steps the genomes *G*_2_, …, *G_n_* are taken one by one and LZ-matches of the form (offset, length), where “offset” is the position of REF where a match of length “length” begins, are sought. The contigs in the current genome are processed in their original order. If a match is not found for the given position (note that such a check takes a constant time, owing to the simplicity of the hash table organization), we move to the next position in the current contig, and so forth, and once we have a (tentative) match, we verify its *k* symbols and try to extend it maximally in both directions (with a restriction that matches cannot cross contig boundaries). The left extension of the current match is allowed to “swallow” the (whole) previous match(es). Surprisingly, this little idea is a powerful optimization trick that improves the compression ratio sometimes by >50% on our datasets and is also moderately beneficial for the compression speed because there are significantly fewer LZ-matches for further encoding. To make this effect even stronger (by up to a few percent), the next position just after a match is *decreased* by *m* (which is 16 by default). Using such a “skip margin” in some cases allows longer matches to be found.

**Figure 4:**
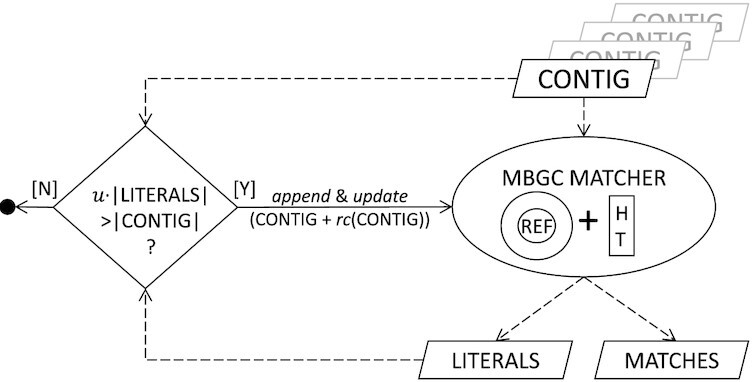
General scheme of the contig matching process with an emphasis on appending the reference buffer strategy.

Finally, the symbols between matches are added to the literal buffer. At this point, we can define the strategy for augmenting the REF string depicted in Fig. 4. Once we are at the end of a contig, the portion of its symbols not covered with matches is checked; if it is large enough (exceeds 1/*u* of the contig length, where *u* = 192 by default), the REF string is appended with the contig and its reverse complement. The rationale is that contigs too similar to some parts of REF are almost completely redundant and thus do not contribute enough to facilitate compression, but increase the memory requirement. This design decision was indeed very successful, as in our test data the string REF together with the concatenated literals often took <2% of the input. If, however, the contigs to compress are not similar enough to the previous ones, the REF string grows quickly and may reach its limit, which depends on the number of genomes in the collection and the size of the first genome (details in the supplementary material, Section 4.3). From this point on, the REF string works like a circular buffer; i.e., instead of being *appended* it is being overwritten from the starting position.

The resulting streams of match data (offsets, lengths), literals, header and filename data, and flags are compressed with LZMA and PPMd, using a well-known open-source software development kit (LZMA SDK).

For easier understanding of the MBGC internals, we created an example (see Supplementary Fig. S5). Moreover, the last section of the supplementary material covers the details of back-end compression of the streams resulting from the matching stage.

### MBGC in the max mode

The description above corresponds to the single-threaded version of our algorithm. MBGC is, however, multi-threaded. MBGC’s max mode (invoked as mbgc -c 3), with preference to the compression ratio rather than compression speed, does not use multithreading except for parallel input, back-end compression, and possibly gzip decompression; to understand such a design decision, see Fig. 1 and the related discussion.

We note that in the max mode the initialization of REF with *G*_1_ is not required. Because matching is sequential, it can be started from *G*_1_ instead of *G*_2_ (even with an empty REF sequence). *G*_1_ will be used to extend REF before matching the remaining genomes.

As multi-threaded matching implementation uses more memory, upper-bounding the reference buffer by 2^32^ bytes in the default mode helps to reduce the memory consumption during the compression of larger collections. On the other hand, in the max mode the buffer is allowed to grow up to 2^40^ bytes, which is beneficial for the compression ratio.

The last major difference between MBGC compression modes concerns back-end compression. To optimize the performance in the default mode, the most time consuming of the resulting streams (i.e., match data and literals) are broken down into blocks and compressed in parallel, sacrificing however some compression ratio.

### Multi-threading

MBGC makes use of the producer-consumer dataflow pattern. Assuming *t* worker threads, we have at most *t* − 1 producers and ≥1 consumer for the compression. The producers decompress and handle the input (gzip) files in parallel and store them in buffers (if the input file is uncompressed, the gzip decompression phase is simply skipped); each producer can handle ≤32 files (genomes) in its buffer. The consumer parses headers and contigs and performs the actual compression (maintaining the hash table, finding LZ-matches, and so forth). Once a producer fills up its buffer, it switches to compress the next unprocessed genome (entering a temporary consumer mode), which serves as a simple load balancing technique.

When a genome is fully encoded, the REF sequence is prolonged with the relevant contigs; updates to REF are performed in a critical section, preserving the original genome order (via a queuing mechanism). Let us explain this issue in more detail. We take care that the area of REF in which a worker looks for matches is not overwritten with newer contigs by other workers. To this end, when a worker *W* begins its job, it marks a guard position in the REF that prevents other workers from overwriting REF beyond this position until *W* terminates processing a current genome. It might mean that some contigs cannot be written to REF and are thus ignored. Fortunately, in our experiments this detrimental effect hampers the compression ratio rather negligibly. When the buffer of a producer is not full, the producer again fills up its buffer by reading and processing the input data, and the consumer proceeds to compress new genomes.

## Availability of Source Code and Requirements

- Project name: MBGC: Multiple Bacteria Genome Compressor
- Project home page: https://github.com/kowallus/mbgc
- Operating systems: Linux
- Programming language: C++
- Other requirements: C++14 standard or higher, cmake 3.4 or higher
- License: e.g., GNU GPL v3.0
- biotools ID: mbgc
- RRID:SCR_021875

## Availability of Supporting Data and Materials

The pathogen datasets underlying this article are available in the NCBI repository: https://www.ncbi.nlm.nih.gov/pathogens.

The yeast datasets (*S. cerevisiae* and *S. paradoxus)* genomes were taken from the Sanger Institute repository: ftp://ftp.sanger.ac.uk/pub/users/dmc/yeast/latest/.

All benchmark data are available online: http://coach.kis.p.lodz.pl/mbgc-datasets/.

Snapshots of our code and other data further supporting this work are openly available in the *GigaScience* repository, GigaDB [25].

## Supplementary Material

giab099_GIGA-D-21-00217_Original_Submission

giab099_GIGA-D-21-00217_Revision_1

giab099_GIGA-D-21-00217_Revision_2

giab099_GIGA-D-21-00217_Revision_3

giab099_Response_to_Reviewer_Comments_Revision_2

giab099_Reviewer_1_Report_Original_Submission Diogo Pratas -- 7/27/2021 Reviewed

giab099_Reviewer_1_Report_Revision_1 Diogo Pratas -- 11/23/2021 Reviewed

giab099_Reviewer_1_Report_Revision_2 Diogo Pratas -- 12/6/2021 Reviewed

giab099_Reviewer_2_Report_Original_Submission Jinyan Li -- 8/12/2021 Reviewed

giab099_Reviewer_2_Report_Revision_1 Jinyan Li -- 11/17/2021 Reviewed

giab099_Reviewer_3_Report_Original_Submission idoia ochoa -- 8/13/2021 Reviewed

giab099_Reviewer_3_Report_Revision_1 idoia ochoa -- 11/28/2021 Reviewed

giab099_Supplemental_File

## Additional Files

**Supplementary Figure S1:** Total times for compressing, transferring, and decompressing large collections of genomes, with network connection speed of 10 Mbit/s (bottom panels) and 100 Mbit/s (top panels).

**Supplementary Figure S2:** Total times for compressing, transferring, and decompressing collections of 1k genomes, with network connection speed of 10 Mbit/s (bottom panels) and 100 Mbit/s (top panels).

**Supplementary Figure S3:** Relative compression ratios and times when 1 of the compression parameters is varied and the remaining parameters keep their default value. The left (respectively right) *y*-axis is related to relative compressed sizes (respectively compression times).

**Supplementary Figure S4:** Relative compression ratios and times when 1 of the compression parameters is varied and the remaining parameters keep their default value. The left (respectively right) *y*-axis is related to relative compressed sizes (respectively compression times).

**Supplementary Figure S5:** Steps of the MBGC compression, on a high level, for a toy example. The parameters used for this example are as follows: seed length 6, *k* = 8, *m* = 2, *u* = 4, *s* = 5. (i) REF is created from the first genome, G1, of length 28 (REF is now the concatenation of G1 and rc(G1), and thus twice as long), and the hash table HT is populated with seeds taken from REF. (ii) Substrings from the first contig are sought in HT, and the found matches are left- and right-extended. Note how the skip margin idea is applied. After the contig is processed, the decision is made whether REF should be extended (affirmative here). (iii) REF is appropriately extended and HT is updated; newer seeds overwrite older ones in the case of a collision. The new seeds in HT are marked red, and additionally with bold typeface when overwriting. (iv) Processing another config. Note how the skip margin idea allows the previously found match to be swallowed (thus reducing the number of output matches). The decision whether REF should be extended is negative this time. (v) The final stream of literals and matches, where matches are only marked with % and contig separators are marked with a semicolon. The prefix of this stream of length 28 is the genome G1.

**Supplementary Table S1:** Compression results—small collections of human genomes (chromosomes separated).

**Supplementary Table S2:** Compression results—small collections of human genomes (ordered by genomes).

**Supplementary Table S3:** Compression results for single-file inputs (single Multi-Multi-FASTA); RNA data.

**Supplementary Table S4:** Compression results for single-file inputs (single Multi-Multi-FASTA); protein data.

**Supplementary Table S5:** Compression results for single-file inputs (single Multi-Multi-FASTA); DNA data.

## Abbreviations

1KGP: 1000 Genomes Project; BSC: Block Sorting Compressor; COMRAD: COMpression using RedundAncy of Dna; CPU: central processing unit; EOL: end-of-line; GCC: GNU Compiler Collection; GDC2: Genome Differential Compressor 2; HRCM: Hybrid Referential Compression Method; MBGC: Multiple Bacteria Genome Compressor; MuGI: Multiple Genome Index; NAF: Nucleotide Archival Format; NCBI: National Center for Biotechnology Information; LZ: Lempel-Ziv; LZMA: Lempel-Ziv-Markov chain-Algorithm; PPMd: Prediction by Partial Matching (variant by Dmitry Shkarin); RAM: random access memory; SSD: solid state drive.
