# Supplementary material for: MBGC: Multiple Bacteria Genome Compressor
Source: Gigascience. 2022 Jan 27;11:giab099. doi: 10.1093/gigascience/giab099 (PMC8848312; doi:10.1093/gigascience/giab099)
Supplement: giab099_GIGA-D-21-00217_Original_Submission [file giab099_giga-d-21-00217_original_submission.pdf]

GigaScience
MBGC: Multiple Bacteria Genome Compressor
--Manuscript Draft--

**Manuscript Number:**	GIGA-D-21-00217
**Full Title:**	MBGC: Multiple Bacteria Genome Compressor
**Article Type:**	Research
**Funding Information:**	
**Abstract:**	\begin{abstract}<br><br>\textbf{Background}<br><br>Genomes within the same species reveal large similarity, exploited by specialized multiple genome compressors.<br><br>The existing algorithms and tools are however targeted at large, e.g., mammalian, genomes, and their performance on bacteria strains is mediocre. \\<br><br>\textbf{Results}<br><br>In this work, we propose MBGC, a specialized genome compressor making use of specific redundancy of bacterial genomes.<br><br>Its characteristic features are finding both direct and reverse-complemented LZ-matches, as well as a careful management of a reference buffer in a multi-threaded implementation.<br><br>Our tool is not only compression efficient, but also fast.<br><br>On a collection of 168,311 bacterial genomes, totalling 587\,GB, we achieve the compression ratio around the factor of 1260, and the compression (resp. decompression) speed around 1630\,MB/s (resp. 760\,MB/s) using<br><br>8 hardware threads, on a computer with a 14-core~/~28-thread CPU and a fast SSD, being about 3.5 times more succinct and more than an order of magnitude faster in the compression than our main competitors.\\<br><br>\end{abstract}
**Corresponding Author:**	Szymon Grabowski, D.Sc.<br>Lodz University of Technology<br>Łódź, POLAND
**Corresponding Author Secondary Information:**	
**Corresponding Author's Institution:**	Lodz University of Technology
**Corresponding Author's Secondary Institution:**	
**First Author:**	Szymon Grabowski, D.Sc.
**First Author Secondary Information:**	
**Order of Authors:**	Szymon Grabowski, D.Sc.<br>Tomasz M. Kowalski, PhD
**Order of Authors Secondary Information:**	
**Additional Information:**	
**Question**	**Response**

Powered by Editorial Manager® and ProduXion Manager® from Aries Systems Corporation

Are you submitting this manuscript to a special series or article collection?	No
**Experimental design and statistics**<br><br>Full details of the experimental design and statistical methods used should be given in the Methods section, as detailed in our Minimum Standards Reporting Checklist. Information essential to interpreting the data presented should be made available in the figure legends.<br><br>Have you included all the information requested in your manuscript?	Yes
**Resources**<br><br>A description of all resources used, including antibodies, cell lines, animals and software tools, with enough information to allow them to be uniquely identified, should be included in the Methods section. Authors are strongly encouraged to cite Research Resource Identifiers (RRIDs) for antibodies, model organisms and tools, where possible.<br><br>Have you included the information requested as detailed in our Minimum Standards Reporting Checklist?	Yes
**Availability of data and materials**<br><br>All datasets and code on which the conclusions of the paper rely must be either included in your submission or deposited in publicly available repositories (where available and ethically appropriate), referencing such data using a unique identifier in the references and in the "Availability of Data and Materials" section of your manuscript.<br><br>Have you have met the above requirement as detailed in our Minimum	Yes

Standards Reporting Checklist?	

Click here to access/download;Manuscript;main.tex

GigaScience, 2021, 1–8

doi: xx.xxxx/xxxx
Manuscript in Preparation
Paper

PAPER

MBGC: Multiple Bacteria Genome Compressor

Szymon Grabowski[1,*,†] and Tomasz M. Kowalski[2,*,†]

[1]Institute of Applied Computer Science, Lodz University of Technology, Poland

[*]{sgrabow,tkowals}@kis.p.lodz.pl
[†]Contributed equally.

Abstract

Background Genomes within the same species reveal large similarity, exploited by specialized multiple genome compressors. The existing algorithms and tools are however targeted at large, e.g., mammalian, genomes, and their performance on bacteria strains is mediocre.
Results In this work, we propose MBGC, a specialized genome compressor making use of specific redundancy of bacterial genomes. Its characteristic features are finding both direct and reverse-complemented LZ-matches, as well as a careful management of a reference buffer in a multi-threaded implementation. Our tool is not only compression efficient, but also fast. On a collection of 168,311 bacterial genomes, totalling 587 GB, we achieve the compression ratio around the factor of 1260, and the compression (resp. decompression) speed around 1630 MB/s (resp. 760 MB/s) using 8 hardware threads, on a computer with a 14-core / 28-thread CPU and a fast SSD, being about 3.5 times more succinct and more than an order of magnitude faster in the compression than our main competitors.

Key words: Algorithms, Data compression, Multiple genome compression, FASTA, Pathogens

Background

Genome compression is a fairly old research topic, dating back to mid-1990s [1]. It was soon realized that even sophisticated techniques for compressing a single genome, e.g., [2], cannot offer much higher compression ratios than simple packing of DNA symbols into 2 bits per each (see also the recent experimental comparison [3]). The interest of researchers thus shifted into relative compression of a genome given a reference [4, 5, 6, 7, 8], typically representing the same species, or compression of a given collection of genomes without an external reference [9, 10, 11]. We focus on the last problem variant.

The abundance of full genomes available in major repositories, like NCBI or 1KGP, in recent years poses a challenge to compress them efficiently, preferably combining high compression ratios, fast compression and decompression, and reasonable memory requirements. In this work, we focus on the compression of bacterial genomes, for which existing genome collection compressors are not appropriate from algorithmic or technical reasons.

Analyses

For the experiments we took a large collection of 168,311 bacterial genomes in the FASTA format from the NCBI Pathogen Detection project, and four 1024-genome subsets of it, each representing a single species (except for a joint subset with E. Coli and Shigella genomes). MBGC and other compressors were tested on a Linux (Debian) machine equipped with a 14-core Intel Core i9-10940X 3.3 GHz CPU, 128 GB of DDR4-RAM (CL 16, clocked at 2666 MHz) and a fast SSD (ADATA 2 TB M.2 PCIe NVMe XPG SX8200 Pro). MBGC is written in C++ and was compiled with gcc 10.2.1. The disk cache was flushed between runs, to have raw reads of the input files from the disk. By the compression ratio we mean the ratio between the original input size and the compressed size. If, for example, the ratio improves from 1000 to 1500, e.g., due to changing some parameters of the compressor, we can say that the compression ratio improves 1.5 times (or by 50%).

For the competitors of MBGC we chose one multiple genome compressor (HRCM) and a few popular high-quality general-purpose compressors (BSC, 7z, zstd).

As it can be seen (Table 1–3), MBGC wins easily in the com-

Compiled on: July 20, 2021.
Draft manuscript prepared by the author.

Key Points

- Bacterial genomes are highly similar, due to repeating direct and reverse-complemented substrings.
- Our bacterial genome compressor, MBGC, achieves compression ratios up to above 1000, much higher than its competitors.
- A careful multi-threaded implementation allows to reach (de)compression speed above 1 GB/s on a mid-end workstation.

pression ratio on the *E. coli*, *L. monocytogenes* and *S. enterica* subsets, as well as on the total collection. It also dominates in the compression times, although not always in the decompression times. Both the compression and the decompression speed of our solution are at least in the order of hundreds of MBs per second, partly due to a multi-threaded implementation. The performance of the specialized genome compressor, HRCM [7], is only mediocre, and we refrained from running it on the whole collection, as the compression would take about a week. We note that some other specialized genome compressors were tried out on our data as well, with no success (for details, see the supplementary data).

Our experiments with MBGC show that the genomes of some bacteria species can be collectively compressed by a factor exceeding 1000, at the (de)compression speed over 1 GB/s. This may be an argument for replacing the dominating gzip compression format (applied to individual genomes) with a much more resource-effective solution in DNA repositories.

MBGC in the default mode is more than an order of magnitude faster than zstd -19 in compression, and marginally faster in the decompression. The gap in the compression ratio between MBGC and zstd grows with larger collections, reaching a factor of over 4.5 for the whole *S. enterica*, and is about 3.5 for the collection of all genomes. On the other hand, zstd is more memory-frugal, which may matter if the experiments are run, e.g., on a standard laptop (MBGC default needs 23 GB of RAM to compress the whole collection). Let us also comment the performance of the stronger MGBC mode. We can notice that MBGC max (which does not use multithreading except for parallel input and possibly gzip decompression; to understand such a design decision, see Fig. 1 and the related discussion further) obtains the compression ratio by a few percent better than the default mode (with the largest gain for the *E. coli* data), but it is 3–4 times slower in the compression. The compression and decompression memory usage remains reasonable (although not as good as for zstd), and the max mode even tends to be more frugal than the default mode.

The results of 7zip -mx=9 were obtained limiting threads usage to 12 (to avoid excessive memory usage during the compression). For smaller collections it is about 8–15 times (resp. 1.5 times) slower in compression (resp. decompression) than zstd (-19) and its compression ratio is usually worse than zstd's on average (although it varies for individual cases). BSC, which is a strong general-purpose compressor based on the Burrows–Wheeler transform (BWT), performed relatively poor on larger pathogen collections, obtaining compression ratio 2–4 times smaller than other competitors. Moreover, it is the slowest in the decompression and is also quite memory-hungry (particularly striking in the decompression) in our experiments, which can be explained by running 14 blocks of (up to) 1 GB each in parallel.

We point out that for the purpose of testing general-purpose tools (zstd, BSC and 7zip) we applied a unified strategy not to hamper their compression in any way. First, the End-Of-Line (EOL) symbols were removed from the DNA strings in the input files prior to the experiment. Second, zstd and BSC can work with a single file input (and output) and for this reason we combined the input into a TAR archive (the preprocessing time for the compression process and the postprocessing time for the decompression process were not included).

Preliminary experiments (with 1k genome collections) show that on the original data (i.e., with EOLs preserved) 7zip needs about 40% more time to compress and its compression ratio is worse by a factor of 2–3. The respective losses are even greater for zstd (around 3–7 in the compression ratio, and 2 in the compression time with regard to the stronger mode). Such striking differences are however understandable; there are many long LZ-matches in our data, which are broken in "random" positions with the EOL characters.

It may be interesting to check the impact of reverse-complement matches on the MBGC performance. It is significant indeed; according to our preliminary experiments, on

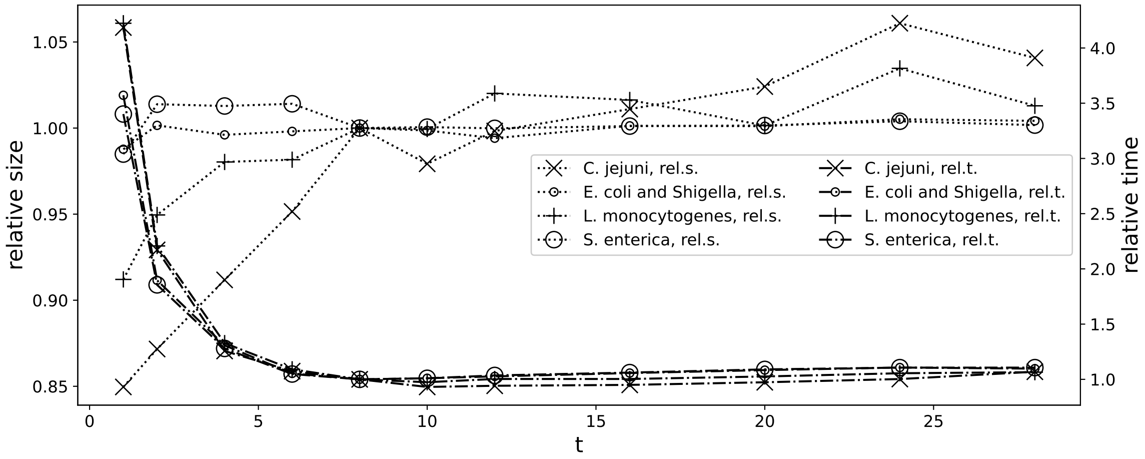

Figure 1. Relative compression ratios and times in the function of the number of threads. The left (resp. right) Y axes are related to relative compressed ratios (resp. compression times).

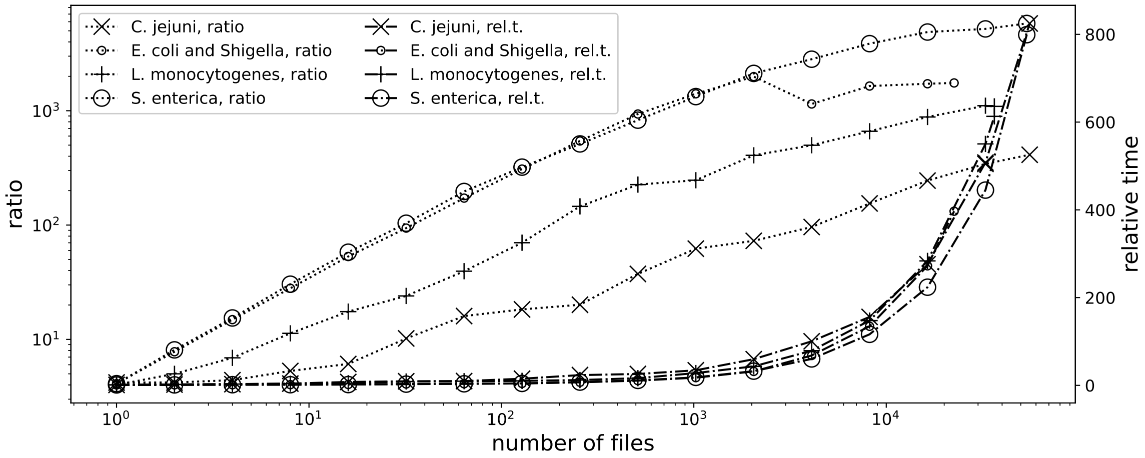

Figure 2. Compression ratios and relative times when the number of input genomes grows. The left (resp. right) Y axes are related to compressed ratios (resp. relative compression times).

C. jejuni and *L. monocytogenes* the compression ratio with RC-matches turned off deteriorates roughly by a factor between 1.1 and 1.7 in the default mode.

Throughout all the presented experiments the input data are in the uncompressed (FASTA) format. Still, MBGC can read gzipped FASTA and we briefly checked how it affects overall performance. The gzipped stream is decompressed with the aid of libdeflate (`https://github.com/ebiggers/libdeflate`), a library for fast whole-buffer Deflate-based decompression (and compression as well, but we use it only for reading). On the individual species collections the compression time gets slightly better (e.g., by even 21% for *S. enterica*), with 12.5% speedup for the whole genome collection; all figures with respect to the default mode of MBGC. The compression ratio varies a little (due to unpredictable access to genomes with the worker threads), usually below 1%.

Fig. 1 shows the compression ratio and compression speed with varying the number of threads from 1 to 28. The speed does not improve with more than 6 threads (but perhaps it would with even more efficient disk I/O). The compression ratio is rather unaffected for *E. coli* and *S. enterica*, but using already more than 1 thread for *C. jejuni* and *L. monocytogenes* yields a few percent compression loss. For *C. jejuni* the gap is as large as about 15% when the number of threads grows from 1 to (the default) 8. On the other hand, using 8 threads is about 3–4 times faster than 1 thread in the compression for all four datasets and for this reason we find the compression loss in half of the cases rather acceptable.

Finally, in Fig. 2 we can see how the compression ratio and compression times change when more and more genomes are given as the input. The number of threads was set to 8 (default). As expected, the compression time grows roughly linearly (note the X-axis scale), but the compression ratio improves, as for further genomes more similar "pieces" can be found in the already processed collection (or, to be more precise, in the currently used *REF* sequence). The only exception is *E. coli*, where after processing about 2,000 genomes the compression ratio first deteriorates somewhat and then no longer improves. This can be easily explained by the heterogeneity of this dataset, which comprises both E. coli and (closely related to E. coli, but different) Shingella genomes.

For a separate experiment, we took two non-bacterial genome collections, *S. cerevisiae* and *S. paradoxus* (Table 4). We didn't expect MBGC to be competitive here, and indeed, GDC 2 and 7z are superior in the compression ratio (while zstd is slightly better than MBGC max), but MBGC remains the fastest tool in the compression process while still maintaining a relatively high ratio. A better overall choice is, however, GDC 2, with a significantly higher compression ratio and slower by only 34% in the compression with respect to MBGC max on *S. cerevisiae*. On the other hand, the compression speed difference is 7-fold (in favor of MBGC max) in case of *S. paradoxus*. In decompression, zstd is the fastest, followed by GDC 2 and 7z, and then by MGDC (default, then max). BSC is also here the slowest in the decompression, and is followed by HRCM, which is, on the other hand, relatively fast in the compression.

In the supplementary data we also present compression results for a small collection of human genomes (hg16, ..., hg19). Although these kinds of data are not the target of MBGC, our tool performs satisfactorily here as well, with quite competitive compression ratios and speed.

Potential implications

The large similarity of bacterial genomes, allowing for compression ratios exceeding the factor of 1000, as shown on the total collection of 168k pathogen individuals, could be directly benefited in genomic repositories for easier backup and replication. A slightly less obvious, but still promising application, could be using the proposed format for rapid download. To this end, the genomes selected by a user to download could then be lumped together and compressed by a factor, say, between 10 and 100 (depending on the count and similarity of the datasets of choice), which is likely to offset the cost of the compression process. It is not clear if and how *caching* compressed groups of genomes downloaded together could improve this process, yet this possibility and resulting tradeoffs seem worth exploring.

Figure 3 presents a combined measure expressed as the total time to transfer (download) the entire collection of our test genomes. Each bar consists of three parts, the compression time (at our test machine), the transfer time (assuming the network connection link of 10 Mbit/s or 100 Mbit/s, on the left and right figure, respectively), and the decompression time (at the same test machine). The used compression switches are: zstd -3 –long=31, pigz -6, mbgc -c 2 (default). The input for MBGC are the original gzip files (as provided in NCBI). The input FASTA files for zstd and pigz were stripped of EOL symbols prior to the compression. The "tar" bars basically correspond to transmitting raw FASTA files, where the compression phase is data tarring (merging), and the decompression phase is data untarring. The "ncbi" bars correspond to the gzip archives in

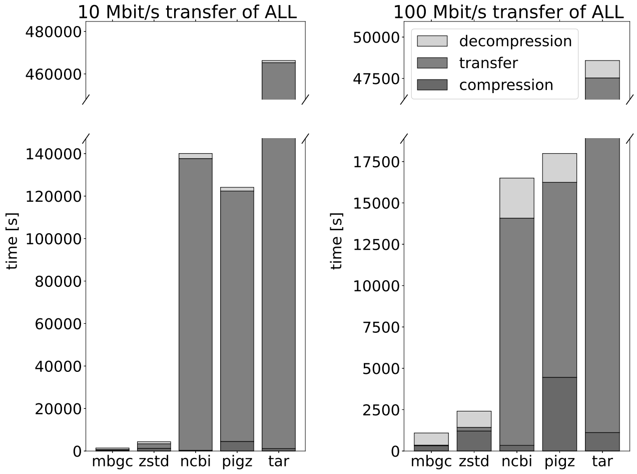

Figure 3. Total times of compressing, transferring and decompressing a collection of 168,311 genomes

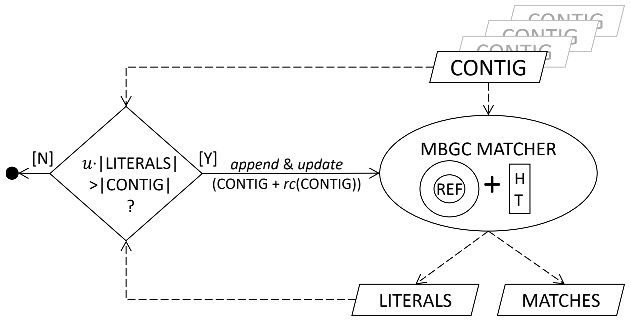

Figure 4. General scheme of the contig matching process with an emphasis on appending the reference buffer strategy

the NCBI repository, where the compression time comprises only tarring the data (so, it is some lower bound estimation). Clearly, MBGC has a huge edge over the competitors, and only zstd comes relatively close with the faster network connection. Note also that even with a faster connection the gzip-based approaches (bars "ncbi" and "pigz", which is multithreaded gzip implementation) are more than an order of magnitude slower than MBGC. The gaps are greater with a slower connection, and for the transfer of 10 Mbit/s the advantage of MBGC over zstd is 3-fold and by a factor of almost 100 over "ncbi". Clearly (cf. also Fig. 2), the gains will be smaller with a smaller amount of data to download at a time, and also the impact of the compression and the decompression times grows with faster networks, making the results generally flatter. Similar figures for species collections of (all and 1k) genomes are included in the supplementary data.

We believe that the ideas behind MBGC can be adapted for a dedicated compressed index for bacterial genomes, allowing for fast pattern counting and reporting. Such an index could handle multi-genome mapping, i.e., mapping sequencing reads against multiple genomes in an efficient way (see, e.g., [12, 13, 14] and references therein). Compressed indexes for repetitive data have been a major research area in the string matching community in the past decade, but few solutions have been tested on a large scale, e.g., hundreds of gigabytes (one exception could be the MuGI index [15] which, for example, can maintain 1092 diploid human genomes in less than 8 GB of space, serving exact pattern queries of length 150 bp in below 80 µs on a commodity PC). Perhaps the major obstacle in running industry-scale experiments were construction costs, both in time and space, for many worst-case oriented indexing data structures. It could be argued that the level of similarity of bacterial genomes allows for relaxing the requirements and focusing on typical, not worst, cases, to obtain practical performance. Although the prospects are not fully clear, it is our opinion that the ideas of MBGC could be adapted to obtain a compressed index for bacterial collections combining high compression ratios, relatively low computational requirements of the construction and short access times.

Methods

There is significant redundancy in bacterial genomes which cannot be fully exploited using existing multiple genome compressors. The standard approach of finding repetitions between the currently processed genome and a reference genome (or possibly all previously processed genomes), and encoding them as LZ-phrases of the form (offset, length), is only moderately successful. We found out that many strings repeat as reverse-complements of corresponding strings from other genomes, a phenomenon known, but surprisingly rarely handled earlier (the COMRAD tool being an exception [16]).

It is also beneficial not to limit the reference to one, or a few, previous genome(s), but to allow finding matches occurring almost anywhere earlier. This, however, requires a potentially unbounded memory buffer. We mitigate this problem with building the reference string, i.e., a reservoir for possible matches, in an incremental manner, appending only blocks which are "new enough", that is, containing a relatively large fraction of DNA subsequences not seen before. This (general) approach, i.e., building "a dictionary of repeats", is known in the context of relative genome compression, see, e.g., [9] and [17].

Basic algorithm

As the key ideas of our solution, Multiple Bacteria Genome Compressor (MBGC), are already sketched, now we present the algorithm in detail. The goal is to compress the sequence of genomes $G_1, \ldots, G_n$ in the FASTA format. The genomes consist of one or many contigs (by a contig, throughout the paper, we mean a sequence in the FASTA file). At the start the reference string *REF* is initialized with G_1 followed by $rc(G_1)$, where $rc(\cdot)$ stands for the reverse complement of the passed string. MBGC also stores a literal buffer, which is initialized with *REF* (but not its reverse-complement). During the compression process, a hash table of fixed size (e.g., 2^{25} slots) is maintained, and the pairs of the form (h, pos) are inserted to it, where the positions *pos* are taken from *REF* accessed sparsely, with a stride of 16 symbols, and h are the hash values of corresponding k-mer seeds taken from the sampled positions. A collision on the hash h overwrites the previous value associated with it.

In the following steps the genomes $G_2, \ldots, G_n$ are taken one by one and LZ-matches of the form (*offset*, *length*) are sought in *REF*. The contigs in the current genome are processed in their original order. If a match is not found for the given position (note that such a check takes a constant time, due to the extremely simple hash table organization), we move to the next position in the current contig, etc., and once we have a (tentative) match, we verify its k symbols and try to extend it maximally in both directions (with a restriction that matches cannot cross contig boundaries). The left extension of the current match is allowed to "swallow" the (whole) previous match(es). Surprisingly, this little idea is a powerful optimization trick which improves the compression ratio sometimes by more than 50% on our datasets, and is also moderately beneficial for the

compression speed, as there is significantly less LZ-matches for further encoding. To make this effect even stronger (by up to a few percent), the next position just after a match is *decreased* by *m* (which is 16 by default). Using such a "skip margin" in some cases allows to find longer matches.

Finally, the symbols between matches are added to the literal buffer. At this point, we can define the strategy for augmenting the *REF* string depicted in Fig. 4. Once we are at the end of a contig, the portion of its symbols not covered with matches is checked; if it is large enough (exceeds 1/192 of the contig length, by default), the *REF* string is appended with the contig and its reverse complement. The rationale is that contigs too similar to some parts of *REF* are almost completely redundant and thus do not contribute enough to facilitate compression, but increase the memory requirement. This design decision was indeed very successful, as in our test data the string *REF* together with the concatenated literals often took less than 2% of the input. If, however, the contigs to compress are not similar enough to the previous ones, the *REF* string grows quickly and may reach its limit, which depends on the number of genomes in the collection and the size of the first genome (details in the supplementary data), and is also upper-bounded by 2^{32} bytes in the current implementation. From this point on, the *REF* string works like a circular buffer, i.e., instead of being *appended* it is being overwritten from the starting position.

Multi-threading

The description above corresponds to the single-threaded version of our algorithm. MBGC is, however, multi-threaded and makes use of the producer-consumer dataflow pattern. Assuming t worker threads, we have at most $t-1$ producers and at least one consumer for the compression. The producers decompress and handle the input (gzip) files in parallel and store them in buffers (if the input file is uncompressed, the gzip decompression phase is simply skipped); each producer can handle up to 32 files (genomes) in its buffer. The consumer parses headers and contigs, and performs the actual compression (maintaining the hash table, finding LZ-matches, etc.). Once a producer fills up its buffer, it switches to compress the next unprocessed genome (entering a temporary consumer mode), which serves as a simple load balancing technique.

When a genome is fully encoded, the *REF* sequence is prolonged with the relevant contigs; updates to *REF* are performed in a critical section, preserving the original genome order (via a queuing mechanism). Let us explain this issue in more detail. We take care that the area of *REF* in which a worker looks for matches is not overwritten with newer contigs by other workers. To this end, when a worker *W* begins its job, it marks a guard position in the *REF* which prevents other workers from overwriting *REF* beyond this position until *W* terminates processing a current genome. It might mean that some contigs cannot be written to *REF* and are thus ignored. Fortunately, in our experiments this detrimental effect hampers the compression ratio rather negligibly. When the buffer of a producer is not full, the producer again fills up its buffer by reading and processing the input data, and the consumer proceeds to compress new genomes.

The resulting streams of match data (offsets, lengths), literals, header and filename data, and flags are compressed with LZMA and PPMd, using a well-known open-source software development kit (LZMA SDK).

Availability of source code and requirements

- Project name: MBGC: Multiple Bacteria Genome Compressor
- Project home page: https://github.com/kowallus/mbgc
- Operating system(s): Linux
- Programming language: C++
- Other requirements: C++14 standard or higher, cmake 3.4 or higher
- License: e.g. GNU GPL v3.0

Availability of supporting data and materials

The pathogen data sets supporting the results of this article are available in the *US National Center for Biotechnology Information* repository: https://www.ncbi.nlm.nih.gov/pathogens.

The yeast datasets (*S. cerevisiae* and *S. paradoxus)* genomes were taken from from *Sanger Institute* repository: ftp://ftp.sanger.ac.uk/pub/users/dmc/yeast/latest/.

All benchmark data are available online: http://coach.kis.p.lodz.pl/mbgc-datasets/.

Declarations

List of abbreviations

1KGP: 1000 Genomes Project; BSC: Block Sorting Compressor; COMRAD: COMpression using RedundAncy of Dna; EOL: End-Of-Line; GCC: GNU Compiler Collection; GDC2: Genome Differential Compressor 2; HRCM: Hybrid Referential Compression Method; MBGC: Multiple Bacteria Genome Compressor; MuGI: Multiple Genome Index; NCBI: National Center for Biotechnology Information; LZ: Lempel-Ziv; LZMA: Lempel-Ziv-Markov chain-Algorithm; PPMd: Prediction by Partial Matching (variant by Dmitry Shkarin).

Ethical Approval

Not applicable.

Consent for publication

Not applicable.

Competing Interests

The authors declare that they have no competing interests.

Funding

This work was partially supported by the Faculty of Electrical, Electronic, Computer, and Control Engineering, Lodz University of Technology, as a statutory activity (both authors).

Author's Contributions

S.G. developed the overall conception and participated in the design of the work and in drafting the manuscript. T.M.K. participated in the design of the work, implemented the tool and conducted all major experiments, and participated in drafting the manuscript. Both authors reviewed and approved the final manuscript.

Table 1. Compression results – collections of 1k genomes.

	HRCM	BSC -p -e2 -b1024	7z -mx=9 -md=1536m	zstd -3 –long=31	zstd -19 –long=31	MBGC default	MBGC -c 3
				C. jejuni (1.78 GB)			
ratio	15.1	30.4	67.5	30.8	51.9	61.3	**73.1**
ctime	196.3	114.4	1051.2	4.8	71.2	**3.8**	12.3
dtime	27.0	70.1	2.8	1.5	**1.4**	3.0	3.7
cmem	1.8	7.9	18.3	1.9	4.2	1.8	**1.2**
dmem	**0.3**	8.8	1.8	1.8	1.8	1.5	1.3
				E. coli (4.87 GB)			
ratio	165.2	115.2	310.7	374.3	463.7	1405.2	**1441.3**
ctime	308.3	127.5	1156.4	12.8	84.8	**3.0**	7.1
dtime	28.9	88.0	4.2	2.9	2.9	**1.7**	**1.7**
cmem	1.9	21.5	49.7	2.3	4.9	2.6	**1.2**
dmem	**0.7**	24.1	4.8	2.2	2.2	1.2	1.2
				L. monocyt. (3.09 GB)			
ratio	39.7	52.9	187.3	80.1	172.6	247.0	**274.9**
ctime	225.5	121.7	1169.3	7.0	147.5	**3.1**	9.3
dtime	26.0	81.6	3.2	2.2	**2.1**	2.3	2.5
cmem	1.8	13.8	31.8	2.3	4.9	2.2	**1.3**
dmem	**0.3**	15.3	3.1	2.2	2.2	1.5	1.4
				S. enterica (5.20 GB)			
ratio	288.0	87.9	295.4	485.1	588.1	1326.4	**1354.1**
ctime	308.3	133.0	1199.1	11.3	134.6	**3.1**	7.1
dtime	28.2	89.3	4.5	3.0	3.0	**1.8**	2.0
cmem	1.9	23.2	53.2	2.3	4.9	2.6	**1.2**
dmem	**0.5**	25.7	5.2	2.2	2.2	1.3	1.3

The rows "ratio" show the ratio of the input to the output size. Compress / decompress times (as "ctime" / "dtime") are given in seconds. The best results in rows are marked bold. HRCM is single-threaded (except for the latter phase where it invokes 7zip), BSC uses 14 threads, 7zip (up to) 12 threads, zstd 28 threads and MBGC 8 threads.

Acknowledgements

Not applicable.

References

1. Grumbach S, Tahi F. Compression of DNA sequences. In: Proc. Data Compression Conference, IEEE; 1993. p. 340–350.
2. Cao MD, et al. A simple statistical algorithm for biological sequence compression. In: Proc. Data Compression Conference, IEEE; 2007. p. 43–52.
3. Kryukov K, et al. Sequence Compression Benchmark (SCB) database—A comprehensive evaluation of reference-free compressors for FASTA-formatted sequences. GigaScience 2020;9(7):1–12.
4. Christley S, et al. Human genomes as email attachments. Bioinformatics 2009;25(2):274–275.
5. Pavlichin DS, et al. The human genome contracts again. Bioinformatics 2013;29(17):2199–2202.
6. Ochoa I, et al. iDoComp: a compression scheme for assembled genomes. Bioinformatics 2015;31(3):626–633.
7. Yao H, et al. HRCM: An Efficient Hybrid Referential Compression Method for Genomic Big Data. BioMed Research International;2019:3108950.
8. Liu Y, et al. Allowing mutations in maximal matches boosts genome compression performance. Bioinformatics 2020;36(18):4675–4681.
9. Deorowicz S, Grabowski S. Robust relative compression of genomes with random access. Bioinformatics 2011;27(21):2979–2986.
10. Wandelt S, Leser U. FRESCO: Referential compression of highly similar sequences. IEEE/ACM Transactions on Computational Biology and Bioinformatics 2013;10(5):1275–1288.
11. Deorowicz S, et al. GDC 2: Compression of large collections of genomes. Sci Rep 2015;5:11565.
12. Gagie T, Puglisi S. Searching and Indexing Genomic Databases via Kernelization. Front Bioeng Biotechnol 2015;3.
13. Kuhnle A, et al. Efficient Construction of a Complete Index for Pan-Genomics Read Alignment. J Comput Biol 2020;27(4):500–513.
14. Sherman RM, Salzberg SL. Pan-genomics in the human genome era. Nature Reviews Genetics 2020;21:243–254.
15. Danek A, et al. Indexes of Large Genome Collections on a PC. PLoS ONE 2014;9(10):1–12.
16. Kuruppu S, et al. Iterative Dictionary Construction for Compression of Large DNA Data Sets. IEEE ACM Trans Comput Biol Bioinform 2012;9(1):137–149.
17. Kuruppu S, et al. Reference Sequence Construction for Relative Compression of Genomes. In: SPIRE, vol. 7024 of Lecture Notes in Computer Science Springer; 2011. p. 420–425.

Table 2. Compression results – large species collections.

	BSC -p -e2 -b1024	7z -mx=9 -md=1536m	zstd -3 -long=31	zstd -19 -long=31	MBGC default	MBGC -c 3
			C. jejuni (98.38 GB)			
ratio	49.3	117.4	75.2	130.0	413.4	**450.6**
ctime	1197.1	13311.0	234.6	6185.0	**91.8**	382.5
dtime	693.4	121.3	136.8	161.6	**101.5**	119.8
cmem	75.1	100.0	**2.3**	4.9	8.7	6.2
dmem	75.6	34.1	**2.2**	**2.2**	5.6	5.0
			E. coli (114.67 GB)			
ratio	56.5	199.7	340.4	496.5	1736.5	**2055.9**
ctime	1445.9	17796.0	236.0	7243.0	**64.9**	209.3
dtime	795.9	145.3	172.0	148.7	93.0	**80.1**
cmem	75.2	100.0	**2.3**	4.9	9.6	3.4
dmem	75.6	34.1	**2.2**	**2.2**	3.3	2.7
			L. monocyt. (112.00 GB)			
ratio	58.3	202.1	136.7	268.9	1086.8	**1154.9**
ctime	1404.8	16613.0	227.3	7351.0	**67.5**	254.2
dtime	789.1	158.3	163.1	203.8	92.7	**88.2**
cmem	75.2	100.0	**2.3**	4.9	8.1	4.0
dmem	75.5	34.0	**2.2**	**2.2**	3.8	3.4
			S. enterica (262.21 GB)			
ratio	92.8	373.4	988.3	1277.3	5793.0	**5859.2**
ctime	3365.5	36400.0	516.5	11169.0	**125.1**	338.2
dtime	1774.6	369.1	393.0	404.4	**289.2**	292.6
cmem	75.1	99.9	**2.3**	4.9	11.5	3.1
dmem	75.4	34.0	**2.2**	**2.2**	2.5	2.4

The rows “ratio” show the ratio of the input to the output size. Compress / decompress times (as “ctime” / “dtime”) are given in seconds. The best results in rows are marked bold. BSC uses 14 threads, 7zip (up to) 12 threads, zstd 28 threads and MBGC 8 threads.

Table 3. Compression results – mixed species collections.

	BSC -p -e2 -b1024	7z -mx=9 -md=1536m	zstd -3 -long=31	zstd -19 -long=31	MBGC default	MBGC -c 3
			168,311 genomes (587.26 GB)			
ratio	66.9	219.4	215.2	364.7	1264.5	**1421.2**
ctime	7198.0	82924.0	1204.0	32078.0	**361.0**	1222.3
dtime	3901.0	886.1	987.6	923.2	**773.3**	801.8
cmem	75.2	100.0	**2.3**	4.9	23.0	12.1
dmem	75.6	34.1	**2.2**	**2.2**	8.7	8.5
			$4 \times$ 1k genomes (14.94 GB)			
ratio	63.3	182.7	124.6	209.7	220.5	**351.5**
ctime	186.2	2617.9	34.3	622.0	**13.5**	36.2
dtime	102.0	14.8	9.7	**8.6**	9.4	9.1
cmem	66.2	98.7	2.3	4.9	4.7	**2.2**
dmem	73.8	14.9	**2.2**	**2.2**	3.3	2.8

The rows “ratio” show the ratio of the input to the output size. Compress / decompress times (as “ctime” / “dtime”) are given in seconds. The best results in rows are marked bold. BSC uses 14 threads, 7zip (up to) 12 threads, zstd 28 threads and MBGC 8 threads.

Table 4. Compression results on non-bacterial genome collections, *S. cerevisiae* and *S. paradoxus*.

	S. cerevisiae					S. paradoxus				
	ratio	ctime	dtime	cmem	dmem	ratio	ctime	dtime	cmem	dmem
HRCM	78.8	7.13	2.85	1.18	**0.06**	52.6	8.30	3.18	1.18	**0.07**
GDC 2	**109.8**	4.12	0.57	**0.52**	0.14	80.7	21.92	0.82	0.51	0.17
BSC -p -e2 -b1024	52.9	38.74	3.78	2.44	2.44	33.8	33.97	3.67	2.15	2.15
7z -mx=9 -md=1536	100.6	340.98	0.75	4.92	0.50	**83.9**	312.07	0.68	4.41	0.44
zstd -3 –long=31	45.1	**1.17**	0.45	0.56	0.49	30.3	**1.00**	0.38	**0.50**	0.43
zstd -19 –long=31	91.7	51.05	**0.40**	1.21	0.49	66.8	52.44	**0.37**	1.07	0.43
MBGC default	87.5	1.69	0.83	1.92	0.78	49.8	2.07	1.07	1.85	0.73
MBGC -c 3	90.2	3.08	1.05	1.52	0.86	61.2	3.16	1.19	1.45	0.72

The columns "ratio" show the ratio of the input to the output size. Compress / decompress times (as "ctime" / "dtime") are given in seconds. The best results in rows are marked bold. HRCM is single-threaded (except for the latter phase where it invokes 7zip), BSC uses 14 threads, 7zip (up to) 12 threads, zstd 28 threads and MBGC 8 threads.

Click here to access/download
Supplementary Material
MBGC_supplementary.pdf
