# Supplementary material for: MBGC: Multiple Bacteria Genome Compressor
Source: Gigascience. 2022 Jan 27;11:giab099. doi: 10.1093/gigascience/giab099 (PMC8848312; doi:10.1093/gigascience/giab099)
Supplement: giab099_GIGA-D-21-00217_Revision_1 [file giab099_giga-d-21-00217_revision_1.pdf]

GigaScience

MBGC: Multiple Bacteria Genome Compressor

--Manuscript Draft--

**Manuscript Number:**	GIGA-D-21-00217R1
**Full Title:**	MBGC: Multiple Bacteria Genome Compressor
**Article Type:**	Research
**Funding Information:**	
**Abstract:**	\begin{abstract}<br>\textbf{Background}<br>Genomes within the same species reveal large similarity, exploited by specialized multiple genome compressors.<br>The existing algorithms and tools are however targeted at large, e.g., mammalian, genomes, and their performance on bacteria strains is mediocre. \\<br>\textbf{Results}<br>In this work, we propose MBGC, a specialized genome compressor making use of specific redundancy of bacterial genomes.<br>Its characteristic features are finding both direct and reverse-complemented LZ-matches, as well as a careful management of a reference buffer in a multi-threaded implementation.<br>Our tool is not only compression efficient, but also fast.<br>On a collection of 168,311 bacterial genomes, totalling 587\,GB, we achieve the compression ratio around the factor of 1265,<br>and the compression (resp. decompression) speed around 1580\,MB/s<br>(resp.\ 780\,MB/s) using 8 hardware threads, on a computer with a 14-core~/~28-thread CPU and a fast SSD, being<br>almost 3 times more succinct and over 6 times faster in the compression than the next best competitor.\\<br>\end{abstract}<br>\begin{keywords}<br>Algorithms, Data compression, Multiple genome compression, FASTA, Pathogens<br>\end{keywords}
**Corresponding Author:**	Szymon Grabowski, D.Sc.<br>Lodz University of Technology<br>Łódź, POLAND
**Corresponding Author Secondary Information:**	
**Corresponding Author's Institution:**	Lodz University of Technology
**Corresponding Author's Secondary Institution:**	
**First Author:**	Szymon Grabowski, D.Sc.
**First Author Secondary Information:**	
**Order of Authors:**	Szymon Grabowski, D.Sc.
	Tomasz M. Kowalski, PhD
**Order of Authors Secondary Information:**	
**Response to Reviewers:**	We are grateful for Reviewers' suggestions concerning the usability of the MBGC tool. Together with some other improvements we decided to apply the suggestions where it was possible, and have released a new version. The major changes in the new version are:<br>* it supports standard input and output during (de)compression,<br>* option to compress a collection of genomes stored in a single FASTA file,<br>* higher reference buffer size limit (2^40 bytes) in the max mode.<br>We are aware of the following minor bug: during decompression MBGC may report a wrong number of extracted files (too large by one) in case of collections compressed in

Powered by Editorial Manager® and ProduXion Manager® from Aries Systems Corporation

max mode. We are going to release a fix (in our next revision).

The manuscript is modified and extended in many places (all changes in the main paper and the supplementary material are marked in color), including:

* extended Background section, with more references given and a broader perspective,
* experiments with a broader list of competitors (for some of them, the experiments were not successful, which is explained in detail in the suppl. mat.), in particular: NAF is added to tables in the main paper,
* section Method: extended discussion of the experimental results, explaining some methodological choices or software limitations,
* more systematic introduction of MBGC techniques and involved parameters,
* more details in the tables and figures (e.g., in their captions); note also that the top 3 or 4 tools in each category (e.g., compression ratio or decompression time) are marked in tables with a number in a superscript,
* colored lines in the figures,
* updated software versions (zstd, 7-zip, BSC, together with MBGC),
* Suppl. Material: added single FASTA file mode experiments and their discussion (sect. 2 and 4),
* Suppl. Material: MBGC parameters are now better explained (sect. 4.3),
* Suppl. Material: Fig. 5 (steps of the MBGC compression, on a high level) added,
* Suppl. Material: sect. 5 (MBGC backend compression) added,
* a couple of cosmetic changes throughout the main and supplementary manuscript.

Apart from the changes in the manuscript, the webpage of MBGC (https://github.com/kowallus/mbgc/) is now extended; e.g., exemplary data and scripts demonstrating usage of MBGC in basic compression scenarios are now provided.
Also, now it is possible to install MBGC via conda:
conda install -c bioconda mbgc

Please note also that in the max mode of MBGC the large reference buffer support (which was added recently, as signalled above) may naturally lead to an increased memory usage, hence, some internal memory management change (namely, we resigned from boosting the value of 'o' parameter) was also introduced, to mitigate this growth in practice.

Reviewer \#1: This article presents a new compressor that uses both direct and reverse-complemented LZ-matches with multi-threaded and cache optimizations. Generally, the reported results of this tool are exciting, and once confirmed, they have good applicability in the bioinformatics community.
However, I could not reproduce the results by lack of instructions, the benchmark is not representative of the state-of-the-art, and there are also several associated questions. Below the comments are specified.

Regarding the experiments:

1. The experiments could not be reproduced. Unfortunately, the instructions and documentation are not clear (See below my tentatives).

RESPONSE:
We tried our best to make re-running the experiments easier. More details can be found in the responses below.

2. The benchmarking is missing several well-known tools (for example, naf, geco3, Deliminate, MFCompress, Leon, ...). See, for example:

Kryukov, Kirill, et al. "Nucleotide Archival Format (NAF) enables efficient lossless reference-free compression of DNA sequences." Bioinformatics 35.19 (2019): 3826-

Powered by Editorial Manager® and ProduXion Manager® from Aries Systems Corporation

3828.
Silva, Milton, et al. "Efficient DNA sequence compression with neural networks." GigaScience 9.11 (2020): giaa119.
Yao, Haichang, et al. "Parallel compression for large collections of genomes." Concurrency and Computation: Practice and Experience (2021): e6339.

To access more compressors, please see the following benchmark (that is already cited in the article):
https://academic.oup.com/gigascience/article/9/7/giaa072/5867695

RESPONSE:
We thank for the suggestion. We tested the suggested compressors, i.e., naf, geco3, DELIMINATE, MFCompress, Leon, but only naf (both in the main paper and the supplementary material) and DELIMINATE (in the supplementary material) were added to the experiments. The reasons to omit the others are as follows:
* geco3: the compression ratio roughly comparable with BSC and HRCM, but much slower in compression,
* Leon: cannot decompress some datasets (segmentation fault), is rather slow in compression and its compression ratio is somewhat worse than DELIMINATE's,
* MFCompress: quite bad compression ratio, slow decompression (roughly symmetric in performance),
* MtGC (earlier name: MtHRCM): decompression failed in our tests. (As a side note, it does compress stronger than its predecessor, HRCM, but at a price of slower compression.)

DELIMINATE had generally rather mediocre compression ratio and is slow in decompression (roughly symmetric in performance). Still, it performed a little better in compression of some single file repetitive datasets, so we decided to add it to the results presented in supplementary experiments.

We are aware there are more compression tools for genomic data available, but our selection was motivated by a general overview of the literature, and in particular, the comprehensive benchmarks in the Kryukov et al. 2020 paper that you pointed out above. In other words, we added the most promising compressors, NAF (which was relatively successful in our experiments) and DELIMINATE (which didn't perform too well).

Regarding the manuscript:

1. The State-of-the-art in genomic data compression (or at least in collections of genomes) is brief and does not offer a consistent and diverse description of the already developed tools.

RESPONSE:
Now the first paragraph of the Background section is prolonged, with new references and basic descriptions of several outstanding compressors.
Also the second paragraph now mentions other possible features of compression tools (but examining them closer goes beyond the scope of this work).

2. "By the compression ratio we mean the ratio between the original input size and the compressed size. If, for example, the ratio improves from 1000 to 1500, e.g., due to changing some parameters of the compressor, we can say that the compression ratio improves 1.5 times (or by 50%)."
This sentence seems a little confusing (at least for me). Please, rephrase.

RESPONSE:
We changed the corresponding passage to:
"By the compression ratio we mean the ratio between the original input size and the compressed size, e.g., reducing a 500\,MB input to 500\,kB results in the compression ratio of 1000.
Also, if the ratio improves from 1000 to 1500, e.g., due to changing some parameters of the compressor, we can say that the compression ratio improves by a factor of 1.5

(or by 50\%)."

3. "The performance of the specialized genome compressor, HRCM [7], is only mediocre, and we refrained from running it on the whole collection, as the compression would take about a week.".
The purpose of a data compressor can be very different: to use in machines with lower RAM, for compression-based analysis, for long-term storage, research purposes, among others.
The qualification of HRCM without putting it into context seems to be depreciative.

RESPONSE:
We agree that compressors may be evaluated according various criteria (please also see the last paragraph of "Background" in the revised manuscript).
Yet, throughout this paper, we focus on the most basic ones: compression ratio, (de)compression speed and (de)compression memory, where the memory usage is, in a sense, least important here.
Or to make the last statement more concrete: it does matter if two compressors with comparable compression ratio differ in, say, decompression speed: 100\,MB/s vs 200\,MB, but it makes little practical change (on a standard machine) if one of them needs 1\,GB of RAM to decompress a large collection and the other twice more.
In other words, using less memory is good, but below some level makes little difference in typical scenarios.
For these reasons, we kept this sentence almost unchanged ("about a week" -> "several days").

Regarding the tool and documentation:

1. Although I have downloaded and compiled the tool, I had to dedicate some minutes to a "libdeflate" default version issue.
The majority of the bioinformatics community uses conda. In order to minimize installation issues for the users, please, provide a conda installation for the proposed tool.
Also, the libdeflate can already be retrieved with conda. Then, with the instructions for the installation of mbgc, please, add this line to mbgc repository:
conda install -c bioconda libdeflate
Notice that this "conda" part is a suggestion that will facilitate the usage of mbgc by the bioinformatics community.

RESPONSE:
Thank you for this suggestion. Now it is possible to install mbgc via:
conda install -c bioconda mbgc

2. Running ./mbgc gives the output:
./mbgc: For compression expected 2 arguments after options (found 0)
try './mbgc -?' for more information
If the menu appears as default (no arguments besides the program's name), it will be much more helpful.

RESPONSE:
Now running ./mbgc presents the basic usage.

3. The program should have a version flag to depict the version of the program (besides the version at the menu).
This feature is essential for integration/implementations (e.g., conda) and to differentiate from eventual new versions to the mbgc software.

RESPONSE:
We have also implemented -v flag to print solely MBGC version information.

4. Please, provide a running example at the help menu (with tiny existing sequences at the repository).

RESPONSE:
We have decided to provide exemplary data and scripts demonstrating usage of MBGC in basic compression scenarios in MBGC repository subfolder.
https://github.com/kowallus/mbgc/tree/master/example-scripts
Besides that we have extended the usage of description in the repositiory readme and in MBGC help menu.

5. Is this characteristic of mbgc a strict property: "decompresses DNA streams 80 bases per line"?
This characteristic may create differences between original files and uncompressed files. Perhaps, having the possibility to have a custom line size would be a valuable feature, at least for data compression scientists to access and compare with other compressors, mainly because it makes the decompressor not completely lossless (although in practice, there is minimal information required to maintain the whole lossless property). Nevertheless, if the program decompresses FASTA data with a unique line size (for DNA bases) of 80 bases, this should also be mentioned in the article (besides what already exists in the repository).

RESPONSE:
Thank you for the suggestion. We have implemented the custom line length option (-l) and decided to print no EOLs within DNA by default. We also added the paragraph in Analyses section to make the issue explicit:
"As a side note, we point out that MBGC accepts EOL symbols in the input, but does not preserve them in the decompressed output (it uses no EOLs in those strings by default or can insert EOLs in regular gaps in DNA strings, as specified by the user)."
And yes, we agree that due to the EOL issue the MBGC compression is not fully lossless.

6. The first impression was that "sequencesListFile" are the IDs of the bacterial genomes, then I found out that they are the URL-suffixes for the FASTA repository.

RESPONSE:
There must have been a misunderstanding. We have used the approach we encountered in general-purpose and FASTA compressors such as 7zip, HRCM or GDC2. The sequencesListFile should contain a list of names of locally stored FASTA files that are to be compressed. To avoid more ambiguities, we have provided:
* an example with sequencesListFile file in the MBGC repository,
* extended usage information in repository readme and MBGC help:
<sequencesListFile> name of text file containing a list of FASTA files (raw or in gz archives) (given in separate lines) for compression.

Then, I start to wonder if mbgc could accept directly the FASTA containing the collection of genomes.
How can the user provide the FASTA file directly? This feature would simplify a lot the usage of mbgc.
Rationale: most of the reconstruction pipelines output multi-FASTA sequences in a single file. Therefore, this feature has direct applicability.
Please, add more information about this in the help print and at the README.
A higher goal would be to have stdin and stdout in compression/decompression as an option and the style of the argument as POSIX (Program Argument Syntax Conventions).
This features are important for building bioinformatics pipelines and perform analysis (especially since the tools seems to be ultra-fast).

RESPONSE:
Thank you for these suggestions. Earlier, we have focused on genome collections in a set of FASTA files. Now we have implemented support for:
* a single "FASTA containing the collection of genomes" (option -i),

Powered by Editorial Manager® and ProduXion Manager® from Aries Systems Corporation

* input from stdin,
* output to stdout (which can be used to pipe a decompression to a single multi-FASTA file).
As far as we are aware, our tool preserves POSIX arguments convention.
We have extended usage information and MBGC help to cover the aforementioned features.

7. Table 1,2,3,4 (and the additional table at supplementary material) have "Compress / decompress times (as "ctime" / "dtime") are given in seconds,"
but no unity reference is provided in the cap to the cmemory and dmemory. Is this value on a GigaByte unity?

RESPONSE:
Yes, in gigabytes. We extended the table captions with such information.

8. The README should provide a small example for testing purposes with the files already available at the repository or by efetch download (see below).

RESPONSE:
As mentioned earlier, examples are now available:
https://github.com/kowallus/mbgc/tree/master/example-scripts

9. The reproducibility is hard to follow:
I had to search for the following procedure to test the software:
wget
https://github.com/kowallus/mbgc/releases/download/v1.1/tested_samples_lists.7z
7z e tested_samples_lists.7z
After the cere download, also
tar -vzxf cere_assemblies.tgz
Then, I realized that it was missing the sequences, and by the NCBI interface, I lost track. I gave up after a few segmentation faults/combinations without understanding if the program or the settings generated the issue.

RESPONSE:
First, we would like to comment the above procedure. We didn't obtain yeast genomes from NCBI. After extracting the tar archive you should be able to find the sequences in genome.fa FASTA files located in strains subfolders, e.g.:
./strains/YS9/assembly/genome.fa
./strains/K11/assembly/genome.fa
./strains/DBVPG1788/assembly/genome.fa
The full list of the sequences after extracting tested_samples_lists.7z (using 7zip 'e' option) should be inside the current folder in cere_39 file (can be checked using 'cat cere_39' command). Assuming that MBGC binary is located in a programs' folder included in the system path (e.g., inside /usr/local/bin), in order to compress cere sequences we need to switch to cere (cd cere) folder and run:
mbgc ../cere_39 cere.mbgc
With regard to the example above, we have created a script (with some minor modifications) to reproduce MBGC compression and decompression on yeast datasets:
http://coach.kis.p.lodz.pl/mbgc-datasets/scripts/test-yeast.sh

Please provide supplementary material and README with the complete instructions to reproduce the experiments (the exact commands).

Also, this simple way to download a multi-FASTA file with Escherichia Coli sequences may be helpful:

conda install -y -c conda-forge -c bioconda -c defaults entrez-direct
esearch -db nucleotide -query "Escherichia coli" | efetch -format fasta >
Escherichia.mfa

RESPONSE:
We are sorry for lack of precision regarding MBGC usage. The supplementary materials to facilitate reproduction of experiments are located on our server: http://coach.kis.p.lodz.pl/mbgc-datasets
They are divided into several subfolders with the following contents:
* collections - data used in experiments with FASTA files collections (pathogens, yeast and human) in compressed formats together with scripts to extract the data (without or with appropriate EOLs in DNA sequence),
* lists - the sequencesListFiles for the above experiments (the same files are in tested_samples_lists.7z available with github release),
* singlefile - data used in experiments regarding compression of individual FASTA files (DNA, RNA and proteins) in compressed formats together with scripts to extract the data (without or with appropriate EOLs in sequences),
* scripts - bash scripts to recreate main experiments.
In the future, we are planning to make the data available at gigaDB service.

Thank you for all your comments.

Reviewer \#2: This paper proposed a compression algorithm to compress sets of bacterial genome sequences. The motivation is based on the reason that the existing algorithms and tools are targeted at large, e.g., mammalian, genomes, and their performance on bacteria strains is unknown. The key idea of the proposed method is to detect characteristic features from both the direct and reverse-complemented copies of the reference genome via LZ-matching. The compression ratio is high and the compression speed is fast. Specifically, on a collection of 168,311 bacterial genomes (587 GB in file size), the algorithm achieved a compression ratio around the factor of 1260. The author claimed that the performance is much better than the existing algorithms. Overall, the quality of the paper is quite good.

I have two suggestions for the author to improve the manuscript:

1/ it's not clear to me about this sentence that "we focus on the compression of bacterial genomes, for which existing genome collection compressors are not appropriate from algorithmic or technical reasons." More clarifications are needed.

RESPONSE:
We extended the quoted sentence with a parenthetical note: "(e.g., ignoring reverse-complemented matches, slow compression of long DNA sequences interspaced with EOL symbols, lack of N symbol support or constraints concerning the number of sequences in a single FASTA file)".

2/ With my own experience, GDC2 has a better performance on virus genome collections
than HRCM. It's strongly suggested for the author to add the performance of GDC2 on the bacterial genome collections.

RESPONSE:
Please note we did briefly describe unsuccessful experiments with several tools, including GDC 2 in the supplementary data in the subsection "Other tools" ("GDC 2 refused to compress due to uneven number of contigs in the input files."). To make things more clear though (and also in response to a remark from another Reviewer), we added a sentence in the main text, in "Analysis": "Some well-known multiple genome compressors, namely GDC 2 [11], iDoComp [6] and memRGC [8], are not used in our (main) experiments, for the reasons explained in the supplementary data." Still, we made GDC 2 usable for our (bacterial data), which is described in section 4.2 (Other tools) of the supplementary material.
It says: "GDC 2 refused to compress due to uneven number of contigs in the input files. We made it work by prior concatenation of all sequences within each input file. Its compression ratio on 1024-genome collections (Table 1 in the main paper) was rather good, between MBGC and NAF, but the compression time was usually more than 10

times longer than 7z's, the slowest competitor in that experiment."

Thank you for your comments.

Reviewer \#3: The authors present a novel tool for the compression of collections of bacterial genomes. The authors present sound results that demonstrate the performance gain of their tool, MBGC, with respect to the state-of-the-art. As such, I do not have concerns about the method itself. My main concerns are with respect the description of the tool, and how the results are presented. Next I list some of my suggestions (in no particular order):

Main Paper:

- Analysis section: Before naming MBGC specify that it is the proposed tool.

RESPONSE:
In the first paragraph of this section we changed MBGC -> The proposed MBGC.

- Analysis section: Reference for HRCM. Mention here also that other tools such as iDoComp, GDC2, etc. are discussed in the Supplementary (this way the reader knows more tools were analyzed or at least tried on the data).

RESPONSE:
The reference for HRCM is added.
We added a sentence in the main text, in "Analysis":
"Some well-known multiple genome compressors, namely GDC 2 [11], iDoComp [6] and memRGC [8], are not used in our (main) experiments, for the reasons explained in the supplementary data."

- Analysis section: The paragraph "Our experiments with MBGC show that... " is a little misleading, since it seems that the tool has the capacity to compress a collection and just extract a single genome from it. This becomes clear later in the text when it is discussed how the tool could be used to speed up the download of a collection of genomes from a repository. So maybe explain that in more detail here, or mention that it could be used to compress a bunch of genomes prior to download. And then point to the part of the text where this is discussed in more detail.

RESPONSE:
Thank you for this comment. Actually, we found the paragraph misplaced, and decided to use it in the beginning of "Potential implications" section. Now, it introduces the context for collective genomes downloading scenario. We have also modified this paragraph (e.g., removed misleading comment concerning gzip format: "applied to individual genomes") hoping that our message is now more clear.

- Analysis section: The results talk about the "stronger MGBC mode", the "MGBC max",
but in the tables it reads "MBGC default" or "MBGC -c 3". I assume "MBGC -c 3" refers to "MBGC max", but it is not stated anywhere. maybe better to call it "MBGC default" and "MBGC max".

RESPONSE:
Indeed. We changed "MBGC -c 3" to "MBGC max".

- Analysis section: Although the method is explained later in the text, it would be a good idea to give a sense of the difference between the default and max modes of the tool. Or some hints on the trade-off between the two. Also, the parameter "-c 3" is never explained.

RESPONSE:
Now in "Methods", after the subsection "Basic algorithm" we have a short subsection "MBGC in the max mode". We hope that finding the differences between the default and the max mode is now easier to spot.

- Analysis section: Figures, it is difficult to see the trade-off between relative size and relative time, can you use colored lines? such that the same color refers to the same set of genomes. Also, in the caption, explain if we want small or high relative size and time. it may be clear, but better to clearly state it.

RESPONSE:
Thank for you the suggestions. We added colored lines in the figures and also "smaller is better" kind of remarks in the captions.

- Analysis section: there is a sentence that says "all figures w.r.t. the default mode of MBCG". It would be good also to state that in the caption, so that the reader knows which mode of the tool is being used to generate the presented results. and if the input files are gzipped or not. For example, for the following paragraph that starts with Fig. 1, it is not clear if the files are gzipped or not.

RESPONSE:
We added "with respect to the default mode of MBGC" in the figure captions.
Concerning the other suggestion, we have such information in the text: "Throughout all the presented experiments (except for those presented in Fig. 3, in MBGC and ``ncbi" scenarios) the input data are in the uncompressed (FASTA) format.", but also added a similar note in the caption of Fig. 3.
We also changed the phrase "all figures with respect to the default mode of MBGC" in the text to "all numbers...", to make an unambiguous reference to values preceding the phrase.

- Analysis section: First time GDC2 is mentioned, the first thing that comes to mind is why it was not used for the bacterial experiments. See my previous point on having a couple of sentences about the other tools that were considered, and why they are not included in the main tables/figures.

RESPONSE:
Sect. 4.2 (Other tools) of the supplementary material explains briefly why some genomic compressors were problematic in our experiments.
With regard to GDC 2, we write there: "GDC 2 refused to compress due to uneven number of contigs in the input files. We made it work by prior concatenation of all sequences within each input file. Its compression ratio on 1024-genome collections (Table 1 in the main paper) was rather good, between MBGC and NAF, but the compression time was usually more than 10 times longer than 7z's, the slowest competitor in that experiment."

- Methods:

-- Here I am really missing a diagram explaining the main steps of the tool. It seems the paper has been rewritten slightly to fit the format of the journal and some things are not in the correct order. For example, it says the key ideas are already sketched, but i do not think that is true.

RESPONSE:
By the "key ideas" which "are already sketched" we simply meant: LZ77-style compression, handling reverse-complement matches and the reference string ("i.e., a reservoir for possible matches"); all those ideas are mentioned briefly in the two starting paragraphs of "Methods", just before the subsection "Basic algorithm". To emphasize this, we moved the sentence:
"As the key ideas of our solution, Multiple Bacteria Genome Compressor (MBGC), are already sketched, now we present the algorithm in detail."

Powered by Editorial Manager® and ProduXion Manager® from Aries Systems Corporation

before "Basic algorithm" subsection title.
Actually, the main steps of our tool are:
1. contig matching process (shown in Fig. 4), preceded with simple REF & HT initialization,
2. backend compression (discussed more thoroughly in the last section of suppl. mat.).
An additional diagram wouldn't help much, but we added the following sentences at the beginning of "Basic algorithm" section:
"The main stages of MBGC compression are the contig matching process and the backend compression of matching products. Below we focus on explaining the former, essential stage."

-- (offset, length) i assume refers to the position of the REF where the match begins, and the length of the match, but again, not really explained. A diagram would help. Also, when it is time to compress the pairs, are the offset delta encoded? or encoded as they are with a general compressor?

RESPONSE:
Offset is now better defined in the second paragraph of "Basic algorithm".
The offsets are not delta encoded prior to backend compression.
The example we created (Fig. 5 in the suppl. mat.) now shows how matches are encoded.

-- How are the produced tokens (offset, length, literals, etc.) finally encoded?

RESPONSE:
We added a short section 5 (MBGC backend compression) to the supplementary material, which explains how MBGC's streams are finally encoded.

-- First time parameter "k" is mention, default value? Also, how can you do a left extension and "swallow" the previous match? is it because the previous match could have been at another position? otherwise if it was in that position it would have been already extended to the right, correct? i mean, it would have generated a longer match.

RESPONSE:
Yes, you guessed correctly. And thanks for the pointing out that such details were perhaps not clearly presented. We added quite a comprehensive example in the supplementary material, which demonstrates our parsing (=match search/extension) procedure.
Concerning the parameter k: the default value is 32. It is given in the second paragraph of sect. 4.3 of the suppl. material ("The parameter k, whose default value is 32, is the minimum match length.").

-- The "skip margin" idea is not well explained. not sure why the next position after a match is decreased by m. please explain better or use a diagram with an example.

RESPONSE:
The rationale behind the "skip margin" idea is to increase opportunities of finding a better match than the recent one (or even several recent matches). It's actually a trade-off; in an extreme approach, we could start looking for matches through all positions of the current match, but it would kill the performance (speed-wise). Experimentally, we found that just after a match it is beneficial to look for the next match not right at the current position, but at the position decreased by m=16. It helps slightly with ratio and does not deteriorate the compression speed.
Note that fewer, but longer, matches not only result in higher compression ratio but they also shorten the time of backend compression as well as decompression.
We hope that the example we created (Fig. 5 in the suppl. mat.) will help to understand how it works (apart from the brief description in the third paragraph of section 4.3).

Powered by Editorial Manager® and ProduXion Manager® from Aries Systems Corporation

-- when you mention 1/192, maybe already state that this is controlled by the parameter u. otherwise when you mention the different parameters is difficult to relate them to the explanation of the algorithm.

RESPONSE:
Fixed (we replaced 1/192 with "1/u of the contig length, where u = 192 by default").

Availability of supp...

-- from from (typo)

RESPONSE:
Fixed.

Tables

-- Specify the number of genomes in each collection.

RESPONSE:
Now those numbers are given in the respective tables.

-- change MBGC -c 3 to MBGC max or something similar. (see my previous comment -c
flag is not explained!)

RESPONSE:
Fixed.

Supplementary Material

-- move table 1 after the text for ease of reading

RESPONSE:
We hope that now the location of Table 1 is OK.

-- not clcear if the tool has random access or not. it is discussed the percentage of time (w.r.t. decompreessing the whole collection i believe) that it would take to decompress one of the first gneomes vs one of the last ones. this should be better explained. for example, if we decompress the last genome of the collection we will employ 100\% of the time, right? given that previous genomes are part of REF (potentially). please explain better and discuss this point in the analysis part, not only in the supplementary. seems like an important aspect of the algorithm.

RESPONSE:
We added such a clarification in the first paragraph of section 4 (and no, there is no random access). Decompressing the last of n genomes is faster than decompression of all n genomes due to reduced I/O.

-- I assume this is not possible, but should be discussed as well. can you add a genome to an already compressed collection? this together with the random access capabilities will highlight better the main possible uses of the tool.

RESPONSE:
Also in the first paragraph of section 4 we added the sentence: "We also point out that the current version of MBGC does not allow to update an archive with new genomes."

Powered by Editorial Manager® and ProduXion Manager® from Aries Systems Corporation

	-- section 4.3: here HT is used, and then HT is introduced in the next paragraph. please revise the whole text and make sure everything is in the right order.<br><br>RESPONSE:<br>We fixed the "HT" usage issue.<br><br><br>-- parameter m, please explain better.<br><br>RESPONSE:<br>Additionally, to the provided example (Fig. 5 in the suppl. mat.), we have extended the description of the parameter with the sentence: "The rationale behind this idea is to increase opportunities of finding a better match than the recent one (or even several recent matches).".<br><br><br>-- add colors to figures, it will be easier to read them.<br><br>RESPONSE:<br>We did. The lines in Figs 1-2 are now in color, as well as Figs 3-4 in suppl. mat.<br><br><br>Overall, as I mentioned before, I believe the tool offers significant improvements with respect to the competitors for bacterial genomes, and performs well on non bacterial genomes as well. What should be improved for publication is the description of the method, since at the end of the day is the main contribution, and how the text is presented.<br><br>RESPONSE:<br>Thank you. We hope we addressed well enough your concerns (as well as those from the other reviews) and the presentation is now better.<br><br>Thank you for all your comments.
**Additional Information:**	
**Question**	**Response**
Are you submitting this manuscript to a special series or article collection?	No
**Experimental design and statistics**<br><br><br>Full details of the experimental design and statistical methods used should be given in the Methods section, as detailed in our Minimum Standards Reporting Checklist. Information essential to interpreting the data presented should be made available in the figure legends.<br><br><br>Have you included all the information requested in your manuscript?	Yes
**Resources**<br><br><br>A description of all resources used,	Yes

including antibodies, cell lines, animals and software tools, with enough information to allow them to be uniquely identified, should be included in the Methods section. Authors are strongly encouraged to cite Research Resource Identifiers (RRIDs) for antibodies, model organisms and tools, where possible.<br><br>Have you included the information requested as detailed in our Minimum Standards Reporting Checklist?	
**Availability of data and materials**<br><br>All datasets and code on which the conclusions of the paper rely must be either included in your submission or deposited in publicly available repositories (where available and ethically appropriate), referencing such data using a unique identifier in the references and in the "Availability of Data and Materials" section of your manuscript.<br><br>Have you have met the above requirement as detailed in our Minimum Standards Reporting Checklist?	Yes

Manuscript Click here to access/download;Manuscript;main.tex

OXFORD (GIGA)^n SCIENCE

GigaScience, 2021, 1–9

doi: xx.xxxx/xxxx
Manuscript in Preparation
Paper

PAPER

MBGC: Multiple Bacteria Genome Compressor

Szymon Grabowski[1,*,†] and Tomasz M. Kowalski[2,*,†]

[1]Institute of Applied Computer Science, Lodz University of Technology, Poland

[*]{sgrabow,tkowals}@kis.p.lodz.pl
[†]Contributed equally.

Abstract

Background Genomes within the same species reveal large similarity, exploited by specialized multiple genome compressors. The existing algorithms and tools are however targeted at large, e.g., mammalian, genomes, and their performance on bacteria strains is mediocre.
Results In this work, we propose MBGC, a specialized genome compressor making use of specific redundancy of bacterial genomes. Its characteristic features are finding both direct and reverse-complemented LZ-matches, as well as a careful management of a reference buffer in a multi-threaded implementation. Our tool is not only compression efficient, but also fast. On a collection of 168,311 bacterial genomes, totalling 587 GB, we achieve the compression ratio around the factor of 1265, and the compression (resp. decompression) speed around 1580 MB/s (resp. 780 MB/s) using 8 hardware threads, on a computer with a 14-core / 28-thread CPU and a fast SSD, being almost 3 times more succinct and over 6 times faster in the compression than the next best competitor.

Key words: Algorithms, Data compression, Multiple genome compression, FASTA, Pathogens

Background

Genome compression is a fairly old research topic, dating back to mid-1990s [1]. It was soon realized that even sophisticated techniques for compressing a single genome, e.g., [2], cannot offer much higher compression ratios than simple packing of DNA symbols into 2 bits per each (see also the recent experimental comparison [3]). The interest of researchers thus shifted into relative compression of a genome given a reference [4, 5, 6, 7, 8], typically representing the same species, or compression of a given collection of genomes without an external reference [9, 10, 11]. Some of those proposals apply quite advanced techniques (e.g., GDC 2 [11], GeCo3 [12]), while other use rather simple input preprocessing followed by a general-purpose backend compressor, like 7zip in DELIMINATE [13] or zstd in NAF [14]. For example, GDC 2 uses two-pass LZ77 matching and the matches in the latter pass can be built of several matches found in the former pass, to obtain unsurpassed compression ratios on large human genome collections (e.g., the ratio of around 9,500 on 1092 human diploid genomes). GeCo3 [12] combines the power of neural networks with specific DNA models, but its compression on a 2–4 gigabyte genomic collections already takes hours. Allowing mismatches (mutations) in matches, leveraging thus a generalized notion of a standard LZ-match, proved successful in MemRGC [8], a relative compressor for a single genome.

The abundance of full genomes available in major repositories, like NCBI or 1KGP, in recent years poses a challenge to compress them efficiently, preferably combining high compression ratios, fast compression and decompression, and reasonable memory requirements. In this work, we focus on the compression of bacterial genomes (without an external reference), for which existing genome collection compressors are not appropriate from algorithmic or technical reasons (e.g., ignoring reverse-complemented matches, slow compression of long DNA sequences interspaced with EOL symbols, lack of N symbol support or constraints concerning the number of sequences in a single FASTA file). We note that there exist also other possible aspects of compressors (or compression-based tools), e.g., random access support [15, 9, 16] or searching directly in the compressed data, also in an approximate manner [17]. For more references, see the survey [18].

Compiled on: November 10, 2021.
Draft manuscript prepared by the author.

Key Points

- Bacterial genomes are highly similar, due to repeating direct and reverse-complemented substrings.
- Our bacterial genome compressor, MBGC, achieves compression ratios up to above 1000, much higher than its competitors.
- A careful multi-threaded implementation allows to reach (de)compression speed above 1 GB/s on a mid-end workstation.

Analyses

For the experiments we took a large collection of 168,311 bacterial genomes in the FASTA format from the NCBI Pathogen Detection project, and four 1024-genome subsets of it, each representing a single species (except for a joint subset with E. Coli and Shigella genomes). The proposed MBGC and other compressors were tested on a Linux (Debian) machine equipped with a 14-core Intel Core i9-10940X 3.3 GHz CPU, 128 GB of DDR4-RAM (CL 16, clocked at 2666 MHz) and a fast SSD (ADATA 2 TB M.2 PCIe NVMe XPG SX8200 Pro). MBGC is written in C++ and was compiled with gcc 10.2.1. The disk cache was flushed between runs, to have raw reads of the input files from the disk. By the compression ratio we mean the ratio between the original input size and the compressed size, e.g., reducing a 500 MB input to 500 kB results in the compression ratio of 1000. Also, if the ratio improves from 1000 to 1500, e.g., due to changing some parameters of the compressor, we can say that the compression ratio improves by a factor of 1.5 (or by 50%).

For the competitors of MBGC we chose one multiple genome compressor (HRCM [7]), one more versatile bioinformatics data oriented compressor (NAF), and a few popular high-quality general-purpose compressors (BSC, 7zip, zstd). Note that NAF makes use of zstd as its backend compressor. Our selection is based on practical performance of the tools, concerning the compression ratio and (de)compression speed, within reasonable memory requirements. Some well-known multiple genome compressors, namely GDC 2 [11], iDoComp [6] and memRGC [8], are not used in our (main) experiments, for the reasons explained in the supplementary material.

As it can be seen (Table 1–3), MBGC in the max mode wins easily in the compression ratio on the *E. coli*, *L. monocytogenes* and *S. enterica* subsets, as well as on the total collection. MBGC with the default settings also usually dominates over the rest of the contenders, yet 7zip sometimes wins over it by a few percent, due to its large (4 GB) sliding window; the same feature also makes 7zip the most memory consuming (per worker thread) tool. The only case where 7zip beats MBGC max in the compression ratio is the 1024-genome *C. jejuni* collection (Table 1). MBGC also dominates in the compression times, although not always in the decompression times. Both the compression and the decompression speed of our solution are at least in the order of hundreds of MBs per second, partly due to a multi-threaded implementation. The most successful case is arguably the large *S. enterica* collection (Table 2), where MBGC default slightly exceeds 2 GB/s of the compression speed, achieving the compression ratio of 5786, which contrasts with compression ratio of 1312 from 11 times slower NAF, and of 696 from 481 times slower 7zip. The performance of the specialized genome compressor, HRCM, is only mediocre, and we refrained from running it on the whole collection, as the compression would take several days.

MBGC in the default mode is more than an order of magnitude faster than NAF -19 in compression, and at least twice faster in the decompression. The gap in the compression ratio between MBGC and NAF (in its stronger mode) grows with larger collections, reaching a factor of 4.4 for the whole *S. enterica*, and is about 2.9 for the collection of all genomes. On the other hand, NAF is more memory-frugal, which may matter if the experiments are run, e.g., on a standard laptop (MBGC default needs 23 GB of RAM to compress the whole collection). Zstd (experiments of its default mode -3 are only presented) is significantly faster than NAF with the same settings, but offers a noticably worse compression, sometimes even twice. Let us also comment the performance of the stronger MGBC mode. We can notice that MBGC max obtains the compression ratio by a few percent better than the default mode (with the largest gain for the 4×1024 collection) but it is 2.5–4 times slower in the compression. The compression and decompression memory usage remains reasonable (although not as good as for NAF and zstd), and the max mode even tends to be more frugal than the default mode.

The results of 7zip were obtained limiting threads usage to 6 (to avoid excessive memory usage during the compression). For smaller collections it is by more than an order of magnitude slower in compression (but faster in the decompression) than NAF (-19) while its compression ratio is usually comparable to NAF's (although it varies for individual cases). BSC, which is a strong general-purpose compressor based on the Burrows–Wheeler transform (BWT), performed relatively poor on larger pathogen collections, obtaining compression ratio a few times smaller than other competitors. Moreover, it is the slowest in the decompression and is also quite memory-hungry (particularly striking in the decompression) in our experiments, which can be explained by running 12 blocks of (up to) 2 GB each in parallel.

We point out that for the purpose of testing general-purpose tools (zstd, BSC and 7zip) we applied a unified strategy not to hamper their compression in any way. First, the End-Of-Line (EOL) symbols were removed from the DNA strings in the input files prior to the experiment. As a side note, we point out that MBGC accepts EOL symbols in the input, but does not preserve them in the decompressed output (it uses no EOLs in those strings by default or can insert EOLs in regular gaps in DNA strings, as specified by the user). Second, zstd and BSC work with a single file input (and output) and for this reason we combined the input into a TAR archive (the preprocessing time for the compression process and the postprocessing time for the decompression process were not included).

Preliminary experiments (with 1024 genome collections) show that on the original data (i.e., with EOLs preserved) 7zip needs about 40% more time to compress and its compression ratio is worse by a factor of 2–3. The respective losses are even greater for zstd (around 3–7 in the compression ratio, and 2 in the compression time with regard to the stronger mode). Such striking differences are however understandable; there are many long LZ-matches in our data, which are broken in "random" positions with the EOL characters.

It may be interesting to check the impact of reverse-complement matches on the MBGC performance. It is significant indeed; according to our preliminary experiments, on *C. jejuni* and *L. monocytogenes* the compression ratio with RC-matches turned off deteriorates roughly by a factor between 1.1 and 1.7 in the default mode.

Throughout all the presented experiments (except for those

presented in Fig. 3, in MBGC and "ncbi" scenarios) the input data are in the uncompressed (FASTA) format. Still, MBGC can read gzipped FASTA and we briefly checked how it affects overall performance. The gzipped stream is decompressed with the aid of libdeflate (https://github.com/ebiggers/libdeflate), a library for fast whole-buffer Deflate-based decompression (and compression as well, but we use it only for reading). On the individual species collections the compression time gets slightly better (e.g., by even 21% for *S. enterica*), with 12.5% speedup for the whole genome collection (all numbers with respect to the default mode of MBGC). The compression ratio varies a little (due to unpredictable access to genomes with the worker threads), usually below 1%.

Fig. 1 shows the compression ratio and compression speed with varying the number of threads from 1 to 28. The speed does not improve with more than 6 threads (but perhaps it would with even more efficient disk I/O). The compression ratio is rather unaffected for *E. coli* and *S. enterica*, but using already more than 1 thread for *C. jejuni* and *L. monocytogenes* yields a few percent compression loss. For *C. jejuni* the gap is as large as about 15% when the number of threads grows from 1 to (the default) 8. On the other hand, using 8 threads is about 3–4 times faster than 1 thread in the compression for all four datasets and for this reason we find the compression loss in half of the cases rather acceptable.

Finally, in Fig. 2 we can see how the compression ratio and compression times change when more and more genomes are given as the input. The number of threads was set to 8 (default). As expected, the compression time grows roughly linearly (note the X-axis scale), but the compression ratio improves, as for further genomes more similar "pieces" can be found in the already processed collection (or, to be more precise, in the currently used *REF* sequence). The only exception is *E. coli*, where after processing about 2,000 genomes the compression ratio first deteriorates somewhat and then no longer improves. This can be easily explained by the heterogeneity of this dataset, which comprises both E. coli and (closely related to E. coli, but different) Shingella genomes.

For a separate experiment, we took two non-bacterial genome collections, *S. cerevisiae* and *S. paradoxus* (Table 4). We didn't expect MBGC to be competitive here, and indeed, GDC 2 and 7z are superior in the compression ratio but MBGC remains the second fastest (after zstd -3) tool in the compression process while still maintaining a relatively high compression ratio. A better overall choice is, however, GDC 2, with a significantly higher compression ratio and being only slightly slower in the compression than MBGC max on *S. cerevisiae*. On the other hand, the compression speed difference is more than 6-fold, in favor of MBGC max, in case of *S. paradoxus*. In decompression, zstd is the fastest, followed by GDC 2 and 7z, and then by NAF and MBGC. BSC and HRCM are more than twice slower in the decompression than MBGC. Of these two, HRCM is a better pick due to higher compression ratio and relatively fast compression.

In the supplementary material we also present compression results for a small collection of human genomes (hg16, ..., hg19). Although these kinds of data are not the target of MBGC, our tool performs satisfactorily here as well, with quite competitive compression ratios and speed.

Potential implications

Our experiments with MBGC show that the genomes of some bacteria species can be collectively compressed by a factor exceeding 1000, at the (de)compression speed over 1 GB/s (as shown on the total collection of 168k pathogen individuals). This may be an argument for replacing the dominating gzip compression format with a much more resource-effective solution in DNA repositories, both for the end user (i.e., faster dissemination of genomic data) and the data resource management (e.g., easier backup). A slightly less obvious, but still promising application, could be using the proposed format for rapid download. To this end, the genomes selected by a user to download could then be lumped together and compressed by a factor, say, between 10 and 100 (depending on the count and similarity of the datasets of choice), which is likely to offset the cost of the compression process. It is not clear if and how *caching* compressed groups of genomes downloaded together could improve this process, yet this possibility and resulting tradeoffs seem worth exploring.

Fig. 3 presents a combined measure expressed as the total time to transfer (download) the entire collection of our test genomes. Each bar consists of three parts, the compression time (at our test machine), the transfer time (assuming the network connection link of 10 Mbit/s or 100 Mbit/s, on the left and right figure, respectively), and the decompression time (at the same test machine). The used compression switches are: NAF -3 –long=31, pigz -6, mbgc -c 2 (default). The input for MBGC are the original gzip files (as provided in NCBI). The input FASTA files for pigz were stripped of EOL symbols prior to the compression. The "tar" bars basically correspond to trans-

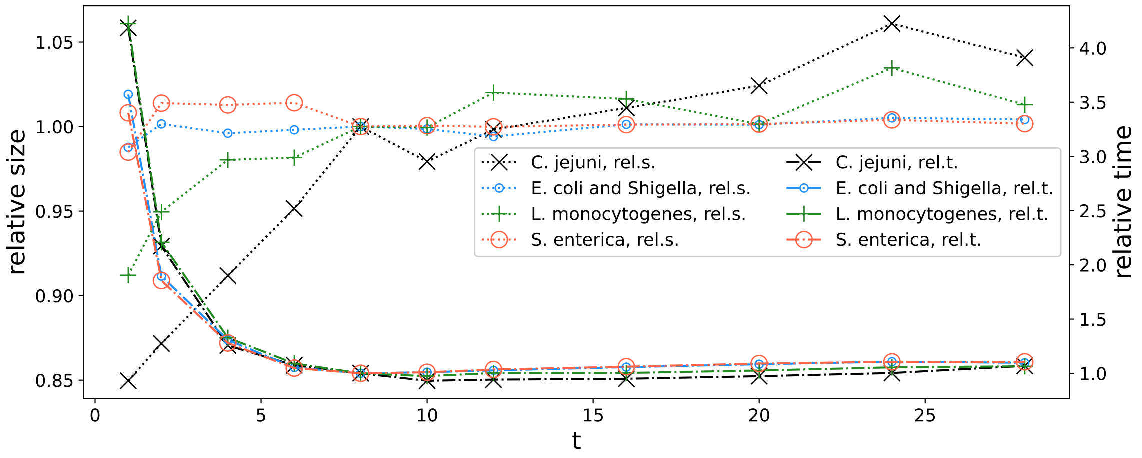

Figure 1. Relative compression ratios and times in the function of the number of threads, with respect to the default mode of MBGC. The left (resp. right) Y axes are related to relative compressed ratios (resp. compression times). On both (left and right) Y axes smaller is better.

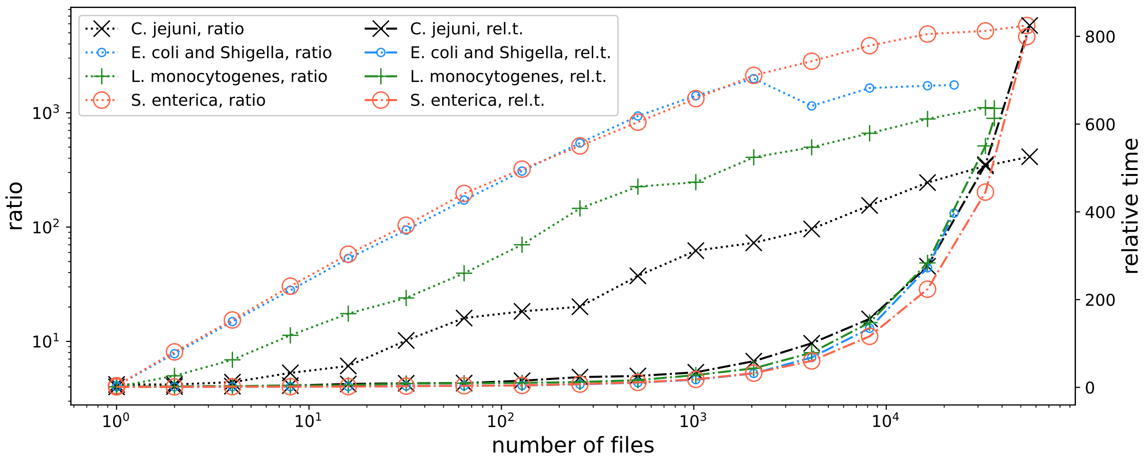

Figure 2. Compression ratios and relative times when the number of input genomes grows, with respect to the default mode of MBGC. The left (resp. right) Y axes are related to compressed ratios (resp. relative compression times). On the left (resp. right) Y axis greater (resp. smaller) values are better.

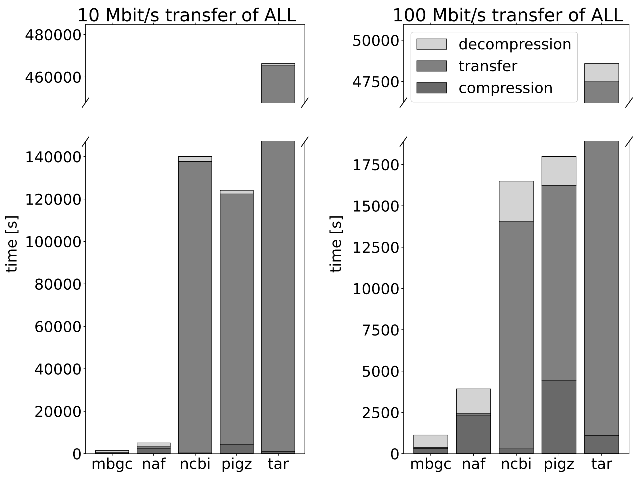

Figure 3. Total times of compressing, transferring and decompressing a collection of 168,311 genomes. The input in the MBGC and "ncbi" scenarios were gzipped FASTA files.

mitting raw FASTA files, where the compression phase is data tarring (merging), and the decompression phase is data untarring. The "ncbi" bars correspond to the gzip archives in the NCBI repository, where the compression time comprises only tarring the data (so, it is some lower bound estimation). Clearly, MBGC has a huge edge over the competitors, and only NAF comes relatively close with the faster network connection. Note also that even with a faster connection the gzip-based approaches (bars "ncbi" and "pigz", which is multithreaded gzip implementation) are more than an order of magnitude slower than MBGC. The gaps are generally greater with a slower connection, and for the transfer of 10 Mbit/s the advantage of MBGC over NAF is more than 3-fold and by a factor of almost 100 over "ncbi". Clearly (cf. also Fig. 2), the gains will be smaller with a smaller amount of data to download at a time, and also the impact of the compression and the decompression times grows with faster networks, making the results generally flatter. Similar figures for species collections of (all and 1024) genomes are included in the supplementary material.

We believe that the ideas behind MBGC can be adapted for a dedicated compressed index for bacterial genomes, allowing for fast pattern counting and reporting. Such an index could handle multi-genome mapping, i.e., mapping sequencing reads against multiple genomes in an efficient way (see, e.g., [18, 19, 20] and references therein). Compressed indexes for repetitive data have been a major research area in the string matching community in the past decade, but few solutions have been tested on a large scale, e.g., hundreds of gigabytes (one exception could be the MuGI index [21] which, for example, can maintain 1092 diploid human genomes in less than 8 GB of space, serving exact pattern queries of length 150 bp in below 80 µs on a commodity PC). Perhaps the major obstacle in running industry-scale experiments were construction costs, both in time and space, for many worst-case oriented indexing data structures. It could be argued that the level of similarity of bacterial genomes allows for relaxing the requirements and focusing on typical, not worst, cases, to obtain practical performance. Although the prospects are not fully clear, it is our opinion that the ideas of MBGC could be adapted to obtain a compressed index for bacterial collections combining high compression ratios, relatively low computational requirements of the construction and short access times.

Methods

There is significant redundancy in bacterial genomes which cannot be fully exploited using existing multiple genome compressors. The standard approach of finding repetitions between the currently processed genome and a reference genome (or possibly all previously processed genomes), and encoding them as LZ-phrases of the form (offset, length), is only moderately successful. We found out that many strings repeat as reverse-complements of corresponding strings from other genomes, a phenomenon known, but surprisingly rarely handled earlier (the COMRAD tool being an exception [22]).

It is also beneficial not to limit the reference to one, or a few, previous genome(s), but to allow finding matches occurring almost anywhere earlier. This, however, requires a potentially unbounded memory buffer. We mitigate this problem with building the reference string, i.e., a reservoir for possible matches, in an incremental manner, appending only blocks which are "new enough", that is, containing a relatively large fraction of DNA subsequences not seen before. This (general) approach, i.e., building "a dictionary of repeats", is known in the context of relative genome compression, see, e.g., [9] and [23].

As the key ideas of our solution, Multiple Bacteria Genome Compressor (MBGC), are already sketched, now we present the algorithm in detail.

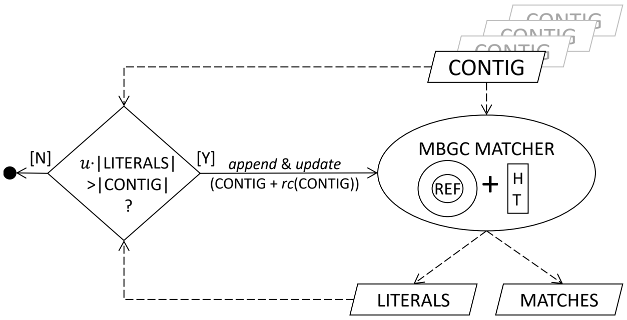

Figure 4. General scheme of the contig matching process with an emphasis on appending the reference buffer strategy

Basic algorithm

The main stages of MBGC compression are the contig matching process and the backend compression of matching products. Below we focus on explaining the former, essential stage.

The goal is to compress the sequence of genomes $G_1, \ldots, G_n$ in the FASTA format. The genomes consist of one or many contigs (by a contig, throughout the paper, we mean a sequence in the FASTA file). At the start the reference string *REF* is initialized with G_1 followed by $rc(G_1)$, where $rc(\cdot)$ stands for the reverse complement of the passed string. MBGC also stores a literal buffer, which is initialized with *REF* (but not its reverse complement). During the compression process, a hash table of fixed size (e.g., 2^{25} slots) is maintained, and the pairs of the form (h, pos) are inserted to it, where the positions *pos* are taken from *REF* accessed sparsely, with a stride of 16 symbols, and h are the hash values of corresponding k-mer seeds taken from the sampled positions. A collision on the hash h overwrites the previous value associated with it.

In the following steps the genomes $G_2, \ldots, G_n$ are taken one by one and LZ-matches of the form (*offset*, *length*), where *offset* is the position of *REF* where a match of length *length* begins, are sought. The contigs in the current genome are processed in their original order. If a match is not found for the given position (note that such a check takes a constant time, due to the extremely simple hash table organization), we move to the next position in the current contig, etc., and once we have a (tentative) match, we verify its k symbols and try to extend it maximally in both directions (with a restriction that matches cannot cross contig boundaries). The left extension of the current match is allowed to "swallow" the (whole) previous match(es). Surprisingly, this little idea is a powerful optimization trick which improves the compression ratio sometimes by more than 50% on our datasets, and is also moderately beneficial for the compression speed, as there are significantly fewer LZ-matches for further encoding. To make this effect even stronger (by up to a few percent), the next position just after a match is *decreased* by m (which is 16 by default). Using such a "skip margin" in some cases allows to find longer matches.

Finally, the symbols between matches are added to the literal buffer. At this point, we can define the strategy for augmenting the *REF* string depicted in Fig. 4. Once we are at the end of a contig, the portion of its symbols not covered with matches is checked; if it is large enough (exceeds $1/u$ of the contig length, where $u = 192$ by default), the *REF* string is appended with the contig and its reverse complement. The rationale is that contigs too similar to some parts of *REF* are almost completely redundant and thus do not contribute enough to facilitate compression, but increase the memory requirement. This design decision was indeed very successful, as in our test data the string *REF* together with the concatenated literals often took less than 2% of the input. If, however, the contigs to compress are not similar enough to the previous ones, the *REF* string grows quickly and may reach its limit, which depends on the number of genomes in the collection and the size of the first genome (details in the supplementary material). From this point on, the *REF* string works like a circular buffer, i.e., instead of being *appended* it is being overwritten from the starting position.

The resulting streams of match data (offsets, lengths), literals, header and filename data, and flags are compressed with LZMA and PPMd, using a well-known open-source software development kit (LZMA SDK).

For easier understanding of the MBGC internals, we created an example (see Fig. 5 in the supplementary material). Moreover, the last section of the supplementary material covers the details of backend compression of the streams resulting from the matching stage.

MBGC in the max mode

The description above corresponds to the single-threaded version of our algorithm. MBGC is, however, multi-threaded. The max MBGC's mode (invoked as `mbgc -c 3`), with preference to the compression ratio rather than compression speed, does not use multithreading except for parallel input, backend compression and possibly gzip decompression; to understand such a design decision, see Fig. 1 and the related discussion.

We note that in the max mode the initialization of *REF* with G_1 is not required. Since matching is sequential, it can be started from G_1 instead of G_2 (even with an empty *REF* sequence). G_1 will be used to extend *REF* before matching the remaining genomes.

As multi-threaded matching implementation uses more memory, upper-bounding the reference buffer by 2^{32} bytes in the default mode helps to reduce the memory consumption during the compression of larger collections. On the other hand, in the max mode the buffer is allowed to grow up to 2^{40} bytes, which is beneficial for the compression ratio.

The last major difference between MBGC compression modes concerns backend compression. To optimize the performance in the default mode, the most time consuming of the resulting streams (i.e., match data and literals) are broken down into blocks and compressed in parallel, sacrificing however some compression ratio.

Multi-threading

MBGC makes use of the producer-consumer dataflow pattern. Assuming t worker threads, we have at most $t - 1$ producers and at least one consumer for the compression. The producers decompress and handle the input (gzip) files in parallel and store them in buffers (if the input file is uncompressed, the gzip decompression phase is simply skipped); each producer can handle up to 32 files (genomes) in its buffer. The consumer parses headers and contigs, and performs the actual compression (maintaining the hash table, finding LZ-matches, etc.). Once a producer fills up its buffer, it switches to compress the next unprocessed genome (entering a temporary consumer mode), which serves as a simple load balancing technique.

When a genome is fully encoded, the *REF* sequence is prolonged with the relevant contigs; updates to *REF* are performed in a critical section, preserving the original genome order (via a queuing mechanism). Let us explain this issue in more detail. We take care that the area of *REF* in which a worker looks for matches is not overwritten with newer contigs by other workers. To this end, when a worker W begins its job, it marks a guard position in the *REF* which prevents other workers from

overwriting *REF* beyond this position until *W* terminates processing a current genome. It might mean that some contigs cannot be written to *REF* and are thus ignored. Fortunately, in our experiments this detrimental effect hampers the compression ratio rather negligibly. When the buffer of a producer is not full, the producer again fills up its buffer by reading and processing the input data, and the consumer proceeds to compress new genomes.

Availability of source code and requirements

- Project name: MBGC: Multiple Bacteria Genome Compressor
- Project home page: https://github.com/kowallus/mbgc
- Operating system(s): Linux
- Programming language: C++
- Other requirements: C++14 standard or higher, cmake 3.4 or higher
- License: e.g. GNU GPL v3.0

Availability of supporting data and materials

The pathogen data sets supporting the results of this article are available in the *US National Center for Biotechnology Information* repository: https://www.ncbi.nlm.nih.gov/pathogens.

The yeast datasests (*S. cerevisiae* and *S. paradoxus)* genomes were taken from *Sanger Institute* repository: ftp://ftp.sanger.ac.uk/pub/users/dmc/yeast/latest/.

All benchmark data are available online: http://coach.kis.p.lodz.pl/mbgc-datasets/.

Declarations

List of abbreviations

1KGP: 1000 Genomes Project; BSC: Block Sorting Compressor; COMRAD: COMpression using RedundAncy of Dna; EOL: End-Of-Line; GCC: GNU Compiler Collection; GDC2: Genome Differential Compressor 2; HRCM: Hybrid Referential Compression Method; MBGC: Multiple Bacteria Genome Compressor; MuGI: Multiple Genome Index; NAF: Nucleotide Archival Format; NCBI: National Center for Biotechnology Information; LZ: Lempel-Ziv; LZMA: Lempel-Ziv-Markov chain-Algorithm; PPMd: Prediction by Partial Matching (variant by Dmitry Shkarin).

Ethical Approval

Not applicable.

Consent for publication

Not applicable.

Competing Interests

The authors declare that they have no competing interests.

Funding

This work was partially supported by the Faculty of Electrical, Electronic, Computer, and Control Engineering, Lodz University of Technology, as a statutory activity (both authors).

Author's Contributions

S.G. developed the overall conception and participated in the design of the work and in drafting the manuscript. T.M.K. participated in the design of the work, implemented the tool and conducted all major experiments, and participated in drafting the manuscript. Both authors reviewed and approved the final manuscript.

Acknowledgements

Not applicable.

References

1. Grumbach S, Tahi F. Compression of DNA sequences. In: Proc. Data Compression Conference, IEEE; 1993. p. 340–350.
2. Cao MD, et al. A simple statistical algorithm for biological sequence compression. In: Proc. Data Compression Conference, IEEE; 2007. p. 43–52.
3. Kryukov K, et al. Sequence Compression Benchmark (SCB) database—A comprehensive evaluation of reference-free compressors for FASTA-formatted sequences. GigaScience 2020;9(7):1–12.
4. Christley S, et al. Human genomes as email attachments. Bioinformatics 2009;25(2):274–275.
5. Pavlichin DS, et al. The human genome contracts again. Bioinformatics 2013;29(17):2199–2202.
6. Ochoa I, et al. iDoComp: a compression scheme for assembled genomes. Bioinformatics 2015;31(3):626–633.
7. Yao H, et al. HRCM: An Efficient Hybrid Referential Compression Method for Genomic Big Data. BioMed Research International 2019;2019:3108950.
8. Liu Y, et al. Allowing mutations in maximal matches boosts genome compression performance. Bioinformatics 2020;36(18):4675–4681.
9. Deorowicz S, Grabowski S. Robust relative compression of genomes with random access. Bioinformatics 2011;27(21):2979–2986.
10. Wandelt S, Leser U. FRESCO: Referential compression of highly similar sequences. IEEE/ACM Transactions on Computational Biology and Bioinformatics 2013;10(5):1275–1288.
11. Deorowicz S, et al. GDC 2: Compression of large collections of genomes. Sci Rep 2015;5:11565.
12. Silva M, et al. Efficient DNA sequence compression with neural networks. GigaScience 2020;9(11). https://doi.org/10.1093/gigascience/giaa119, giaa119.
13. Mohammed MH, et al. DELIMINATE—a fast and efficient method for loss-less compression of genomic sequences: Sequence analysis. Bioinformatics 2012;28(19):2527–2529. https://doi.org/10.1093/bioinformatics/bts467.
14. Kryukov K, et al. Nucleotide Archival Format (NAF) enables efficient lossless reference-free compression of DNA sequences. Bioinformatics 2019;35(19):3826–3828. https://doi.org/10.1093/bioinformatics/btz144.
15. Kuruppu S, et al. Relative Lempel-Ziv Compression of Genomes for Large-Scale Storage and Retrieval. In: Chávez E, Lonardi S, editors. String Processing and Information Retrieval - 17th International Symposium, SPIRE 2010, Los Cabos, Mexico, October 11-13, 2010. Proceedings, vol. 6393 of Lecture Notes in Computer Science Springer; 2010. p. 201–206. https://doi.org/10.1007/978-3-642-16321-0_20.
16. Belazzougui D, et al. Block Trees. Journal of Computer and System Sciences 2021;117:1–22.

Table 1. Compression results – collections of 1024 genomes.

	HRCM	BSC -p -b2047	7z -md4g	zstd -3 -long=31	NAF -3 -long=31	NAF -19 -long=31	MBGC default	MBGC max
				C. jejuni (1.78 GB)				
ratio	15.0	40.2	(1)78.5	31.2	43.0	54.2	(3)62.2	(2)73.1
ctime	196.3	38.0	1064.5	(2)4.0	(3)9.8	136.2	(1)3.7	12.4
dtime	27.0	10.6	(3)3.0	(1)1.5	4.4	4.4	(2)2.8	3.7
cmem	(2)1.76	9.00	18.00	1.92	1.90	2.24	(3)1.78	(1)1.19
dmem	(1)0.28	8.81	1.79	1.76	1.76	1.76	(3)1.45	(2)1.34
				E. coli (4.87 GB)				
ratio	158.2	166.9	377.5	357.8	495.2	(3)530.0	(2)1406.1	(1)1452.3
ctime	308.3	127.4	2228.7	(3)10.0	20.8	57.5	(1)3.0	(2)7.3
dtime	28.9	42.4	5.6	(3)2.9	12.6	11.9	(1)1.5	(1)1.5
cmem	(2)1.88	24.04	45.98	2.31	(3)2.30	2.65	2.55	(1)1.22
dmem	(1)0.66	21.48	4.82	2.15	2.21	2.21	(2)1.17	(2)1.17
				L. monocyt. (3.09 GB)				
ratio	39.3	70.4	(2)268.8	82.9	131.1	163.3	(3)243.0	(1)274.9
ctime	225.5	123.5	1838.1	(2)4.5	13.8	102.8	(1)3.2	(3)9.3
dtime	26.0	36.7	4.0	(1)2.1	7.6	7.6	(1)2.1	(3)2.4
cmem	(2)1.79	15.29	31.82	2.31	2.30	2.63	(3)2.27	(1)1.25
dmem	(1)0.30	15.30	3.07	2.15	2.15	2.15	(3)1.55	(2)1.41
				S. enterica (5.2 GB)				
ratio	268.4	130.0	472.4	427.9	547.5	(3)607.1	(2)1329.8	(1)1356.8
ctime	308.2	130.6	2350.3	(2)6.1	20.4	36.1	(1)3.1	(3)7.3
dtime	28.2	43.0	5.9	(3)3.0	12.8	12.9	(1)1.7	(2)1.9
cmem	(2)1.91	25.67	49.45	2.31	(3)2.30	2.64	2.62	(1)1.22
dmem	(1)0.48	25.68	5.15	2.15	2.17	2.17	(3)1.27	(2)1.26

The rows "ratio" show the ratio of the input to the output size. Compress / decompress times (as "ctime" / "dtime") are given in seconds, memory usages ("cmem" / "dmem") given in GB (G = 10^9). The best three results in a row are marked with a number in parentheses. HRCM is single-threaded (except for the latter phase where it invokes 7zip), BSC uses 12 threads, 7zip (up to) 6 threads, zstd 14 threads and MBGC 8 threads.

17. Rahn R, et al. Journaled string tree–a scalable data structure for analyzing thousands of similar genomes on your laptop. Bioinformatics 2014;30(24):3499–3505. https://doi.org/10.1093/bioinformatics/btu438.
18. Gagie T, Puglisi S. Searching and Indexing Genomic Databases via Kernelization. Front Bioeng Biotechnol 2015;3.
19. Kuhnle A, et al. Efficient Construction of a Complete Index for Pan-Genomics Read Alignment. J Comput Biol 2020;27(4):500–513.
20. Sherman RM, Salzberg SL. Pan-genomics in the human genome era. Nature Reviews Genetics 2020;21:243–254.
21. Danek A, et al. Indexes of Large Genome Collections on a PC. PLoS ONE 2014;9(10):1–12.
22. Kuruppu S, et al. Iterative Dictionary Construction for Compression of Large DNA Data Sets. IEEE ACM Trans Comput Biol Bioinform 2012;9(1):137–149.
23. Kuruppu S, et al. Reference Sequence Construction for Relative Compression of Genomes. In: SPIRE, vol. 7024 of Lecture Notes in Computer Science Springer; 2011. p. 420–425.

Table 2. Compression results – large species collections.

	BSC -p -b2047	7z -md4g	zstd -3 -long=31	NAF -3 -long=31	NAF -19 -long=31	MBGC default	MBGC max
			C. jejuni (55,627 genomes, totalling 98.38 GB)				
ratio	69.7	164.9	74.9	137.1	(3)176.6	(2)412.5	(1)450.6
ctime	969.2	22881.0	(2)211.9	489.5	2570.7	(1)92.7	(3)400.8
dtime	241.3	129.3	(3)127.3	280.3	250.9	(1)78.5	(2)102.3
cmem	128.84	122.54	(2)2.32	(1)2.31	(3)2.66	8.78	7.22
dmem	129.21	34.59	(1)2.15	(3)2.70	(2)2.69	5.62	5.02
			E. coli (22,523 genomes, totalling 114.67 GB)				
ratio	93.1	342.7	242.8	(3)460.9	458.5	(2)1747.4	(1)2051.6
ctime	1195.1	30086.0	(2)165.5	446.3	1313.5	(1)65.0	(3)216.1
dtime	291.6	(3)160.2	164.7	296.0	288.7	(2)83.3	(1)78.4
cmem	128.84	122.48	(1)2.31	(1)2.31	(3)2.66	9.66	10.87
dmem	129.17	34.48	(1)2.15	(3)3.10	(3)3.10	3.25	(2)2.66
			L. monocyt. (36,448 genomes, totalling 112.00 GB)				
ratio	90.2	(3)328.2	137.3	274.9	323.9	(2)1086.9	(1)1160.2
ctime	1166.4	28065.0	(2)162.7	450.1	1805.7	(1)68.0	(3)263.8
dtime	287.6	(3)162.0	184.5	286.4	279.0	(1)84.2	(2)97.2
cmem	128.84	122.48	(2)2.32	(1)2.31	(3)2.66	7.85	7.03
dmem	129.13	34.48	(1)2.15	(2)2.39	(2)2.39	3.82	3.30
			S. enterica (53,713 genomes, totalling 262.21 GB)				
ratio	156.7	695.5	606.0	1205.7	(3)1312.0	(2)5786.0	(1)5881.1
ctime	2655.8	61182.0	(2)290.2	909.9	1423.9	(1)127.3	(3)342.1
dtime	618.0	440.4	(3)396.5	659.9	662.3	(2)270.3	(1)262.0
cmem	128.84	122.46	(1)2.31	(1)2.31	(3)2.66	11.31	10.48
dmem	129.06	34.43	(1)2.15	2.80	2.79	(3)2.51	(2)2.36

The rows “ratio” show the ratio of the input to the output size. Compress / decompress times (as “ctime” / “dtime”) are given in seconds, memory usages (“cmem” / “dmem”) given in GB (G = 10^9). The best three results in a row are marked with a number in parentheses. BSC uses 12 threads, 7zip (up to) 6 threads, zstd 14 threads and MBGC 8 threads.

Table 3. Compression results – mixed species collections.

	BSC -p -b2047	7z -md=4g	zstd -3 -long=31	NAF -3 -long=31	NAF -19 -long=31	MBGC default	MBGC max
			168,311 genomes (587.26 GB)				
ratio	105.3	354.5	193.6	369.0	(3)434.0	(2)1266.6	(1)1411.4
ctime	5824.0	140902.0	(2)846.1	2287.3	7100.0	(1)370.9	(3)1271.6
dtime	1379.7	(3)880.8	970.2	1506.2	1499.7	(1)749.2	(2)757.2
cmem	128.84	122.54	(1)2.31	(1)2.31	(3)2.66	23.06	15.93
dmem	129.22	34.59	(1)2.15	(2)4.51	(3)4.53	8.70	10.73
			4 × 1024 genomes (14.94 GB)				
ratio	88.2	(2)239.2	124.5	177.1	214.6	(3)220.8	(1)351.5
ctime	189.4	4124.0	(2)24.4	62.6	334.6	(1)13.4	(3)36.5
dtime	56.7	15.5	(3)9.9	37.4	37.3	(2)8.9	(1)8.6
cmem	73.79	122.48	(2)2.31	(2)2.31	2.65	4.69	(1)2.22
dmem	73.82	14.83	(1)2.15	(2)2.24	(2)2.24	3.24	2.83

The rows “ratio” show the ratio of the input to the output size. Compress / decompress times (as “ctime” / “dtime”) are given in seconds, memory usages (“cmem” / “dmem”) given in GB (G = 10^9). The best three results in a row are marked with a number in parentheses. BSC uses 12 threads, 7zip (up to) 6 threads, zstd 14 threads and MBGC 8 threads.

Table 4. Compression results on non-bacterial genome collections, *S. cerevisiae* and *S. paradoxus*.

	S. cerevisiae (39 genomes, 486 MB)					S. paradoxus (36 genomes, 429 MB)				
	ratio	ctime	dtime	cmem	dmem	ratio	ctime	dtime	cmem	dmem
GDC 2	(1)109.8	4.12	(2)0.57	(1)0.52	(2)0.14	(2)80.7	21.92	(3)0.82	(2)0.51	(2)0.17
HRCM	78.8	7.13	2.85	1.18	(1)0.06	52.6	8.30	3.18	1.18	(1)0.07
BSC -p -b2047	52.9	10.76	2.65	2.49	2.44	33.8	9.59	2.59	2.21	2.16
7z -md4g	(2)100.6	316.38	(3)0.73	4.92	0.50	(1)83.9	295.95	(2)0.69	4.41	0.44
zstd -3 –long=31	45.8	(1)0.98	(1)0.43	(2)0.54	(3)0.49	30.5	(1)0.87	(1)0.40	(1)0.49	(3)0.43
NAF -3 –long=31	67.0	(3)2.80	1.02	(3)0.63	(3)0.49	43.2	(3)2.61	0.92	(3)0.57	(3)0.43
NAF -19 –long=31	77.0	30.54	1.03	0.97	(3)0.49	43.2	33.54	0.93	0.91	(3)0.43
MBGC default	87.3	(2)1.68	0.82	1.93	0.80	49.1	(2)2.08	1.07	1.84	0.73
MBGC max	(3)90.6	3.72	1.04	1.50	0.87	(3)61.5	3.58	1.24	1.40	0.70

The columns "ratio" show the ratio of the input to the output size. Compress / decompress times (as "ctime" / "dtime") are given in seconds, memory usages ("cmem" / "dmem") given in GB (G = 10^9). The best three results in a column are marked with a number in parentheses. HRCM is single-threaded (except for the latter phase where it invokes 7zip), BSC uses 12 threads, 7zip (up to) 6 threads, zstd 14 threads and MBGC 8 threads.

Supplementary Material

Click here to access/download
Supplementary Material
MBGC_supplementary.pdf

Dear Editor,

We submit a thoroughly revised version of our submission:

Szymon Grabowski, Tomasz M. Kowalski:
"MBGC: Multiple Bacteria Genome Compressor".

We thank the Reviewers for their insightful and detailed comments. We tried our best to address all the remarks and issues pointed out.

The manuscript is modified and extended in many places (all changes in the main paper and the supplementary material are marked in color), including:

* extended Background section, with more references given and a broader perspective,
* experiments with a broader list of competitors (for some of them, the experiments were not successful, which is explained in detail in the suppl. mat.), in particular: NAF is added to tables in the main paper,
* section Method: extended discussion of the experimental results, explaining some methodological choices or software limitations,
* more systematic introduction of MBGC techniques and involved parameters,
* more details in the tables and figures (e.g., in their captions); note also that the top 3 or 4 tools in each category (e.g., compression ratio or decompression time) are marked in tables with a number in a superscript,
* colored lines in the figures,
* updated software versions (zstd, 7-zip, BSC, together with MBGC),
* Suppl. Material: added single FASTA file mode experiments and their discussion (sect. 2 and 4),
* Suppl. Material: MBGC parameters are now better explained (sect. 4.3),
* Suppl. Material: Fig. 5 (steps of the MBGC compression, on a high level) added,
* Suppl. Material: sect. 5 (MBGC backend compression) added,
* a couple of cosmetic changes throughout the main and supplementary manuscript.

Apart from the changes in the manuscript, the webpage of MBGC (https://github.com/kowallus/mbgc/) is now extended; e.g., exemplary data and scripts demonstrating usage of MBGC in basic compression scenarios are now provided. Also the API of our tool is changed/improved and now it is possible to install MBGC via conda:
conda install -c bioconda mbgc

Please note also that the max mode of MBGC was modified: the 2^32 bytes limitation on the REF size was removed. As this large reference buffer support may naturally lead to an increased memory usage, some internal memory management change (namely, we resigned from boosting the value of 'o' parameter) was also introduced, to mitigate this growth in practice.

Our datasets from experiments and auxiliary scripts are temporarily available at
http://coach.kis.p.lodz.pl/mbgc-datasets/

(In the future, we are going to move these data to GigaDB, as required.)

Yours sincerely,
Szymon Grabowski (on behalf of both co-authors)
