# Supplementary material for: MBGC: Multiple Bacteria Genome Compressor
Source: Gigascience. 2022 Jan 27;11:giab099. doi: 10.1093/gigascience/giab099 (PMC8848312; doi:10.1093/gigascience/giab099)
Supplement: giab099_GIGA-D-21-00217_Revision_2 [file giab099_giga-d-21-00217_revision_2.pdf]

GigaScience

MBGC: Multiple Bacteria Genome Compressor

--Manuscript Draft--

**Manuscript Number:**	GIGA-D-21-00217R2
**Full Title:**	MBGC: Multiple Bacteria Genome Compressor
**Article Type:**	Research
**Funding Information:**	
**Abstract:**	\begin{abstract}<br>\textbf{Background}<br>Genomes within the same species reveal large similarity, exploited by specialized multiple genome compressors.<br>The existing algorithms and tools are however targeted at large, e.g., mammalian, genomes, and their performance on bacteria strains is rather moderate. \\<br>\textbf{Results}<br>In this work, we propose MBGC, a specialized genome compressor making use of specific redundancy of bacterial genomes.<br>Its characteristic features are finding both direct and reverse-complemented LZ-matches, as well as a careful management of a reference buffer in a multi-threaded implementation.<br>Our tool is not only compression efficient, but also fast.<br>On a collection of 168,311 bacterial genomes, totalling 587\,GB, we achieve the compression ratio around the factor of 1265,<br>and the compression (resp. decompression) speed around<br>1580\,MB/s<br>(resp.\ 780\,MB/s) using<br>8 hardware threads, on a computer with a 14-core~/~28-thread CPU and a fast SSD, being<br>almost 3 times more succinct and over 6 times faster in the compression than the next best competitor. \\<br>\end{abstract}
**Corresponding Author:**	Szymon Grabowski, D.Sc.<br>Lodz University of Technology<br>Łódź, POLAND
**Corresponding Author Secondary Information:**	
**Corresponding Author's Institution:**	Lodz University of Technology
**Corresponding Author's Secondary Institution:**	
**First Author:**	Szymon Grabowski, D.Sc.
**First Author Secondary Information:**	
**Order of Authors:**	Szymon Grabowski, D.Sc.
	Tomasz M. Kowalski, PhD
**Order of Authors Secondary Information:**	
**Response to Reviewers:**	Thank you for the kind words and providing own results on a few (extra) datasets. We reply to your comments below.<br><br>1. The version flag does not appear in the menu. Please, provide it. I only could retrieve it by<br>entering the usual flag. For non-coding users, it may be a problem.<br><br>RESPONSE:<br>We have prepared a new mbgc release (v1.2.1) to include the suggestions concerning

the menu. The release is also added to conda.
Now the version flag (as well as the version notice) are displayed by default:

./mbgc
Multiple Bacteria Genome Compressor (MBGC) v1.2.1 (c) Szymon Grabowski,
Tomasz Kowalski, 2021

Usage for multiple file compression (list of files given as input):
    mbgc [-c compressionMode] [-t noOfThreads] <sequencesListFile> <archiveFile>
Usage for single file compression:
    mbgc [-c compressionMode] [-t noOfThreads] -i <inputFastaFile> <archiveFile>
Usage for decompression:
    mbgc -d [-t noOfThreads] [-f pattern] [-l dnaLineLength] <archiveFile>
[<outputPath>]

Basic options:
    -c select compression mode (speed: 0; default: 1; repo: 2; max: 3)
    -d decompression mode
    -l format decompressed DNA (i.e., sets the number of bases per row)
    -f decompress files with names containing the given pattern
    -t number of threads used (default: 8)
    -h print full help and exit
    -v print version number and exit

./mbgc -v
Multiple Bacteria Genome Compressor (MBGC) v1.2.1 (c) Szymon Grabowski,
Tomasz Kowalski, 2021

2. Also, the “mbgc: invalid option –” menu is more informative than the MBGC menu.
For example,
I noticed that there are three compression levels (level 1 only for developers) besides
other
parametrization. Perhaps adding a flag for the extended menu at the default menu
would help.

RESPONSE:
Thank you. The flags -c and -h have been added to default options list (see the
previous response).
In the new release we have enabled two additional compression modes oriented
toward the decompression speed.
The changes are fully backwards compatible with the previous release (v1.2) and
the details of the compression modes available earlier have not changed
(except that the default mode can be explicitly called also using -c 1 instead of -c 2
option).
We have also improved the 'invalid option' behavior to display only essential errors and
help flag info:

$ ./mbgc -z
./mbgc: invalid option -- 'z'
./mbgc: For compression using files list as input expected 2 arguments after options
(found 0)
try 'mbgc -h' for more information

3. I got some warnings that can be addressed (in red):
$ mbgc -t 1 -i DS.fa out_DS.fa
SparseEM PARAMETERS: k-mer exact match length = 32; hash pattern length = 28;
HASH_SIZE = 67108864; k1 = 16; k2 = 1; skipMargin = 16
processed reference dataset - 726 [ms]
matching finished - 34315 [ms]
OMP: Info #271: omp_set_nested routine deprecated, please use
omp_set_max_active_levels instead.
OMP: Info #271: omp_set_nested routine deprecated, please use

Powered by Editorial Manager® and ProduXion Manager® from Aries Systems Corporation

	omp_set_max_active_levels instead.<br>OMP: Info #271: omp_set_nested routine deprecated, please use omp_set_max_active_levels instead.<br>OMP: Info #271: omp_set_nested routine deprecated, please use omp_set_max_active_levels instead.<br>compressed 1193491896 bytes to 73588349 (6.166% == x16.2)<br>encoding time - 57456 [ms]<br>(the same flag happens in the decompression process)<br><br>RESPONSE:<br>Thank you for the observation. Unfortunately, it is not easy to get rid of this issue.<br>For one point, we don't have those (or any, in fact) warnings on our platform.<br>Changing the omp_set_nested routine to omp_set_max_active_levels is essentially possible, but unfortunately makes the (parallel) backend compression much slower (in our gcc10 based environment).<br>According to https://syten.eu/docs/faq.html, omp_set_nested warning seems to be a well-known issue, and "this is only a warning which may be annoying but does not hurt. It will go away as soon as the compilers implement OpenMP 5.0 and hence set the _OPENMP macro correctly."<br>We also checked https://gcc.gnu.org/wiki/openmp and the brief report in the top lines suggests that the OpenMP 5.0 specification is not yet fully implemented in gcc (despite progress in recent versions).<br>Moreover, changes in our codes aiming to eliminate the need for omp_set_nested altogether are laborious and can't be implemented in a few days. We intend to introduce an appropriate fix in a future version and we are grateful for your suggestion.<br><br><br>Regarding the article and supplementary material (specially in the last), in my opinion, I would avoid using depreciation expressions, such as “mediocre” (and others) to express classifications about the work/tools developed by other scientists, as well as strong claims using datasets that may not represent the need/purpose of computational biologists / bioinformaticians entirely. Mainly because there are many purposes: if we are constrained with space and RAM it may be worth using high-compression tools at the expense of time. Another purpose is the estimation of the Kolmogorov complexity of genomes.<br><br>RESPONSE:<br>We changed the "mediocre" word, in a few places, to: rather moderate / more or less average etc.<br>Concerning the different purposes of compression tools (or genomic datasets), please note the ending sentences of our Background section:<br>"We note that there exist also other possible aspects of compressors (or compression-based tools), e.g., random access support~\citep{KuruppuPZ10,DG2011,BCGGKNOPT2020} or searching directly in the compressed data, also in an approximate manner~\citep{RWR2014}.<br>For more references, see the survey~\citep{GP2015}."<br><br>That is, we limit our scope to practical multiple genome (de)compression and standard settings (e.g., mid-end workstations).<br><br><br>The recent changes in the manuscript are marked red. Once again, thank you for your review.
**Additional Information:**	
**Question**	**Response**
Are you submitting this manuscript to a special series or article collection?	No
**Experimental design and statistics**	Yes

Powered by Editorial Manager® and ProduXion Manager® from Aries Systems Corporation

Full details of the experimental design and statistical methods used should be given in the Methods section, as detailed in our Minimum Standards Reporting Checklist. Information essential to interpreting the data presented should be made available in the figure legends.<br><br>Have you included all the information requested in your manuscript?	
**Resources**<br><br>A description of all resources used, including antibodies, cell lines, animals and software tools, with enough information to allow them to be uniquely identified, should be included in the Methods section. Authors are strongly encouraged to cite Research Resource Identifiers (RRIDs) for antibodies, model organisms and tools, where possible.<br><br>Have you included the information requested as detailed in our Minimum Standards Reporting Checklist?	Yes
**Availability of data and materials**<br><br>All datasets and code on which the conclusions of the paper rely must be either included in your submission or deposited in publicly available repositories (where available and ethically appropriate), referencing such data using a unique identifier in the references and in the "Availability of Data and Materials" section of your manuscript.<br><br>Have you have met the above requirement as detailed in our Minimum Standards Reporting Checklist?	Yes

Manuscript Click here to access/download;Manuscript;main.tex

OXFORD (GIGA)n SCIENCE

GigaScience, 2021, 1–9

doi: xx.xxxx/xxxx
Manuscript in Preparation
Paper

PAPER

MBGC: Multiple Bacteria Genome Compressor

Szymon Grabowski[1,*,†] and Tomasz M. Kowalski[2,*,†]

[1]Institute of Applied Computer Science, Lodz University of Technology, Poland

*{sgrabow,tkowals}@kis.p.lodz.pl
†Contributed equally.

Abstract

Background Genomes within the same species reveal large similarity, exploited by specialized multiple genome compressors. The existing algorithms and tools are however targeted at large, e.g., mammalian, genomes, and their performance on bacteria strains is rather moderate.
Results In this work, we propose MBGC, a specialized genome compressor making use of specific redundancy of bacterial genomes. Its characteristic features are finding both direct and reverse-complemented LZ-matches, as well as a careful management of a reference buffer in a multi-threaded implementation. Our tool is not only compression efficient, but also fast. On a collection of 168,311 bacterial genomes, totalling 587 GB, we achieve the compression ratio around the factor of 1265, and the compression (resp. decompression) speed around 1580 MB/s (resp. 780 MB/s) using 8 hardware threads, on a computer with a 14-core / 28-thread CPU and a fast SSD, being almost 3 times more succinct and over 6 times faster in the compression than the next best competitor.

Key words: Algorithms, Data compression, Multiple genome compression, FASTA, Pathogens

Background

Genome compression is a fairly old research topic, dating back to mid-1990s [1]. It was soon realized that even sophisticated techniques for compressing a single genome, e.g., [2], cannot offer much higher compression ratios than simple packing of DNA symbols into 2 bits per each (see also the recent experimental comparison [3]). The interest of researchers thus shifted into relative compression of a genome given a reference [4, 5, 6, 7, 8], typically representing the same species, or compression of a given collection of genomes without an external reference [9, 10, 11]. Some of those proposals apply quite advanced techniques (e.g., GDC 2 [11], GeCo3 [12]), while other use rather simple input preprocessing followed by a general-purpose backend compressor, like 7zip in DELIMINATE [13] or zstd in NAF [14]. For example, GDC 2 uses two-pass LZ77 matching and the matches in the latter pass can be built of several matches found in the former pass, to obtain unsurpassed compression ratios on large human genome collections (e.g., the ratio of around 9,500 on 1092 human diploid genomes). GeCo3 [12] combines the power of neural networks with specific DNA models, but its compression on a 2–4 gigabyte genomic collections already takes hours. Allowing mismatches (mutations) in matches, leveraging thus a generalized notion of a standard LZ-match, proved successful in MemRGC [8], a relative compressor for a single genome.

The abundance of full genomes available in major repositories, like NCBI or 1KGP, in recent years poses a challenge to compress them efficiently, preferably combining high compression ratios, fast compression and decompression, and reasonable memory requirements. In this work, we focus on the compression of bacterial genomes (without an external reference), for which existing genome collection compressors are not appropriate from algorithmic or technical reasons (e.g., ignoring reverse-complemented matches, slow compression of long DNA sequences interspaced with EOL symbols, lack of N symbol support or constraints concerning the number of sequences in a single FASTA file). We note that there exist also other possible aspects of compressors (or compression-based tools), e.g., random access support [15, 9, 16] or searching directly in the compressed data, also in an approximate manner [17]. For more references, see the survey [18].

Compiled on: December 3, 2021.
Draft manuscript prepared by the author.

Key Points

- Bacterial genomes are highly similar, due to repeating direct and reverse-complemented substrings.
- Our bacterial genome compressor, MBGC, achieves compression ratios up to above 1000, much higher than its competitors.
- A careful multi-threaded implementation allows to reach (de)compression speed above 1 GB/s on a mid-end workstation.

Analyses

For the experiments we took a large collection of 168,311 bacterial genomes in the FASTA format from the NCBI Pathogen Detection project, and four 1024-genome subsets of it, each representing a single species (except for a joint subset with E. Coli and Shigella genomes). The proposed MBGC and other compressors were tested on a Linux (Debian) machine equipped with a 14-core Intel Core i9-10940X 3.3 GHz CPU, 128 GB of DDR4-RAM (CL 16, clocked at 2666 MHz) and a fast SSD (ADATA 2 TB M.2 PCIe NVMe XPG SX8200 Pro). MBGC is written in C++ and was compiled with gcc 10.2.1. The disk cache was flushed between runs, to have raw reads of the input files from the disk. By the compression ratio we mean the ratio between the original input size and the compressed size, e.g., reducing a 500 MB input to 500 kB results in the compression ratio of 1000. Also, if the ratio improves from 1000 to 1500, e.g., due to changing some parameters of the compressor, we can say that the compression ratio improves by a factor of 1.5 (or by 50%).

For the competitors of MBGC we chose one multiple genome compressor (HRCM [7]), one more versatile bioinformatics data oriented compressor (NAF), and a few popular high-quality general-purpose compressors (BSC, 7zip, zstd). Note that NAF makes use of zstd as its backend compressor. Our selection is based on practical performance of the tools, concerning the compression ratio and (de)compression speed, within reasonable memory requirements. Some well-known multiple genome compressors, namely GDC 2 [11], iDoComp [6] and memRGC [8], are not used in our (main) experiments, for the reasons explained in the supplementary material.

As it can be seen (Table 1–3), MBGC in the max mode wins easily in the compression ratio on the *E. coli*, *L. monocytogenes* and *S. enterica* subsets, as well as on the total collection. MBGC with the default settings also usually dominates over the rest of the contenders, yet 7zip sometimes wins over it by a few percent, due to its large (4 GB) sliding window; the same feature also makes 7zip the most memory consuming (per worker thread) tool. The only case where 7zip beats MBGC max in the compression ratio is the 1024-genome *C. jejuni* collection (Table 1). MBGC also dominates in the compression times, although not always in the decompression times. Both the compression and the decompression speed of our solution are at least in the order of hundreds of MBs per second, partly due to a multi-threaded implementation. The most successful case is arguably the large *S. enterica* collection (Table 2), where MBGC default slightly exceeds 2 GB/s of the compression speed, achieving the compression ratio of 5786, which contrasts with compression ratio of 1312 from 11 times slower NAF, and of 696 from 481 times slower 7zip. The performance of the specialized genome compressor, HRCM, is more or less average, and we refrained from running it on the whole collection, as the compression would take several days.

MBGC in the default mode is more than an order of magnitude faster than NAF -19 in compression, and at least twice faster in the decompression. The gap in the compression ratio between MBGC and NAF (in its stronger mode) grows with larger collections, reaching a factor of 4.4 for the whole *S. enterica*, and is about 2.9 for the collection of all genomes. On the other hand, NAF is more memory-frugal, which may matter if the experiments are run, e.g., on a standard laptop (MBGC default needs 23 GB of RAM to compress the whole collection). Zstd (experiments of its default mode -3 are only presented) is significantly faster than NAF with the same settings, but offers a noticably worse compression, sometimes even twice. Let us also comment the performance of the stronger MGBC mode. We can notice that MBGC max obtains the compression ratio by a few percent better than the default mode (with the largest gain for the 4×1024 collection) but it is 2.5–4 times slower in the compression. The compression and decompression memory usage remains reasonable (although not as good as for NAF and zstd), and the max mode even tends to be more frugal than the default mode.

The results of 7zip were obtained limiting threads usage to 6 (to avoid excessive memory usage during the compression). For smaller collections it is by more than an order of magnitude slower in compression (but faster in the decompression) than NAF (-19) while its compression ratio is usually comparable to NAF's (although it varies for individual cases). BSC, which is a strong general-purpose compressor based on the Burrows–Wheeler transform (BWT), performed relatively poor on larger pathogen collections, obtaining compression ratio a few times smaller than other competitors. Moreover, it is the slowest in the decompression and is also quite memory-hungry (particularly striking in the decompression) in our experiments, which can be explained by running 12 blocks of (up to) 2 GB each in parallel.

We point out that for the purpose of testing general-purpose tools (zstd, BSC and 7zip) we applied a unified strategy not to hamper their compression in any way. First, the End-Of-Line (EOL) symbols were removed from the DNA strings in the input files prior to the experiment. As a side note, we point out that MBGC accepts EOL symbols in the input, but does not preserve them in the decompressed output (it uses no EOLs in those strings by default or can insert EOLs in regular gaps in DNA strings, as specified by the user). Second, zstd and BSC work with a single file input (and output) and for this reason we combined the input into a TAR archive (the preprocessing time for the compression process and the postprocessing time for the decompression process were not included).

Preliminary experiments (with 1024 genome collections) show that on the original data (i.e., with EOLs preserved) 7zip needs about 40% more time to compress and its compression ratio is worse by a factor of 2–3. The respective losses are even greater for zstd (around 3–7 in the compression ratio, and 2 in the compression time with regard to the stronger mode). Such striking differences are however understandable; there are many long LZ-matches in our data, which are broken in "random" positions with the EOL characters.

It may be interesting to check the impact of reverse-complement matches on the MBGC performance. It is significant indeed; according to our preliminary experiments, on *C. jejuni* and *L. monocytogenes* the compression ratio with RC-matches turned off deteriorates roughly by a factor between 1.1 and 1.7 in the default mode.

Throughout all the presented experiments (except for those

presented in Fig. 3, in MBGC and "ncbi" scenarios) the input data are in the uncompressed (FASTA) format. Still, MBGC can read gzipped FASTA and we briefly checked how it affects overall performance. The gzipped stream is decompressed with the aid of libdeflate (https://github.com/ebiggers/libdeflate), a library for fast whole-buffer Deflate-based decompression (and compression as well, but we use it only for reading). On the individual species collections the compression time gets slightly better (e.g., by even 21% for *S. enterica*), with 12.5% speedup for the whole genome collection (all numbers with respect to the default mode of MBGC). The compression ratio varies a little (due to unpredictable access to genomes with the worker threads), usually below 1%.

Fig. 1 shows the compression ratio and compression speed with varying the number of threads from 1 to 28. The speed does not improve with more than 6 threads (but perhaps it would with even more efficient disk I/O). The compression ratio is rather unaffected for *E. coli* and *S. enterica*, but using already more than 1 thread for *C. jejuni* and *L. monocytogenes* yields a few percent compression loss. For *C. jejuni* the gap is as large as about 15% when the number of threads grows from 1 to (the default) 8. On the other hand, using 8 threads is about 3–4 times faster than 1 thread in the compression for all four datasets and for this reason we find the compression loss in half of the cases rather acceptable.

Finally, in Fig. 2 we can see how the compression ratio and compression times change when more and more genomes are given as the input. The number of threads was set to 8 (default). As expected, the compression time grows roughly linearly (note the X-axis scale), but the compression ratio improves, as for further genomes more similar "pieces" can be found in the already processed collection (or, to be more precise, in the currently used *REF* sequence). The only exception is *E. coli*, where after processing about 2,000 genomes the compression ratio first deteriorates somewhat and then no longer improves. This can be easily explained by the heterogeneity of this dataset, which comprises both E. coli and (closely related to E. coli, but different) Shingella genomes.

For a separate experiment, we took two non-bacterial genome collections, *S. cerevisiae* and *S. paradoxus* (Table 4). We didn't expect MBGC to be competitive here, and indeed, GDC 2 and 7z are superior in the compression ratio but MBGC remains the second fastest (after zstd -3) tool in the compression process while still maintaining a relatively high compression ratio. A better overall choice is, however, GDC 2, with a significantly higher compression ratio and being only slightly slower in the compression than MBGC max on *S. cerevisiae*. On the other hand, the compression speed difference is more than 6-fold, in favor of MBGC max, in case of *S. paradoxus*. In decompression, zstd is the fastest, followed by GDC 2 and 7z, and then by NAF and MBGC. BSC and HRCM are more than twice slower in the decompression than MBGC. Of these two, HRCM is a better pick due to higher compression ratio and relatively fast compression.

In the supplementary material we also present compression results for a small collection of human genomes (hg16, . . . , hg19). Although these kinds of data are not the target of MBGC, our tool performs satisfactorily here as well, with quite competitive compression ratios and speed.

Potential implications

Our experiments with MBGC show that the genomes of some bacteria species can be collectively compressed by a factor exceeding 1000, at the (de)compression speed over 1 GB/s (as shown on the total collection of 168k pathogen individuals). This may be an argument for replacing the dominating gzip compression format with a much more resource-effective solution in DNA repositories, both for the end user (i.e., faster dissemination of genomic data) and the data resource management (e.g., easier backup). A slightly less obvious, but still promising application, could be using the proposed format for rapid download. To this end, the genomes selected by a user to download could then be lumped together and compressed by a factor, say, between 10 and 100 (depending on the count and similarity of the datasets of choice), which is likely to offset the cost of the compression process. It is not clear if and how *caching* compressed groups of genomes downloaded together could improve this process, yet this possibility and resulting tradeoffs seem worth exploring.

Fig. 3 presents a combined measure expressed as the total time to transfer (download) the entire collection of our test genomes. Each bar consists of three parts, the compression time (at our test machine), the transfer time (assuming the network connection link of 10 Mbit/s or 100 Mbit/s, on the left and right figure, respectively), and the decompression time (at the same test machine). The used compression switches are: NAF -3 –long=31, pigz -6, mbgc -c 1 (default). The input for MBGC are the original gzip files (as provided in NCBI). The input FASTA files for pigz were stripped of EOL symbols prior to the compression. The "tar" bars basically correspond to trans-

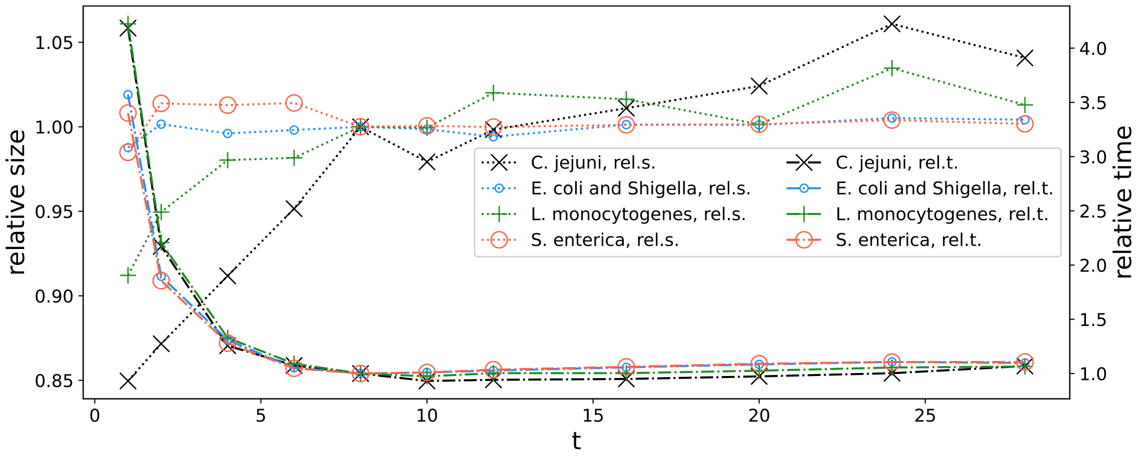

Figure 1. Relative compression ratios and times in the function of the number of threads, with respect to the default mode of MBGC. The left (resp. right) Y axes are related to relative compressed ratios (resp. compression times). On both (left and right) Y axes smaller is better.

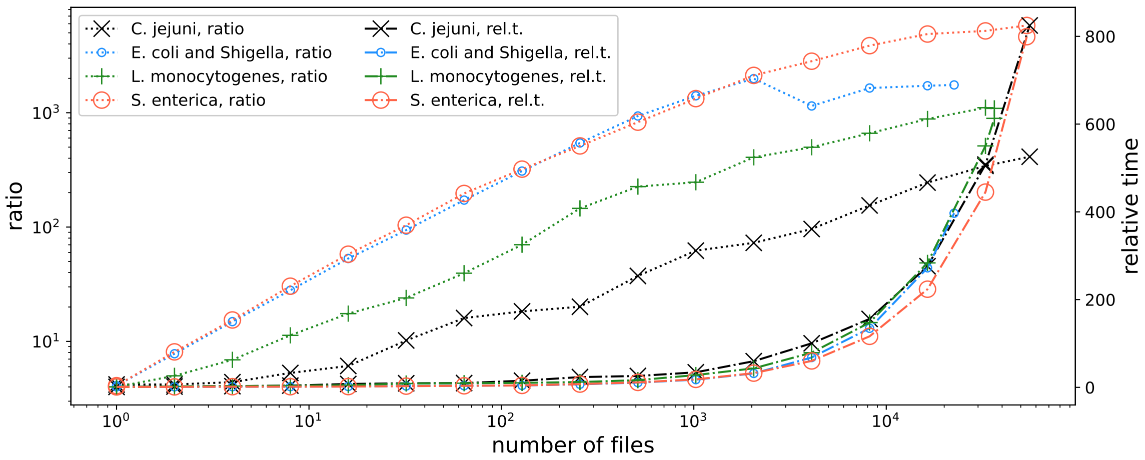

Figure 2. Compression ratios and relative times when the number of input genomes grows, with respect to the default mode of MBGC. The left (resp. right) Y axes are related to compressed ratios (resp. relative compression times). On the left (resp. right) Y axis greater (resp. smaller) values are better.

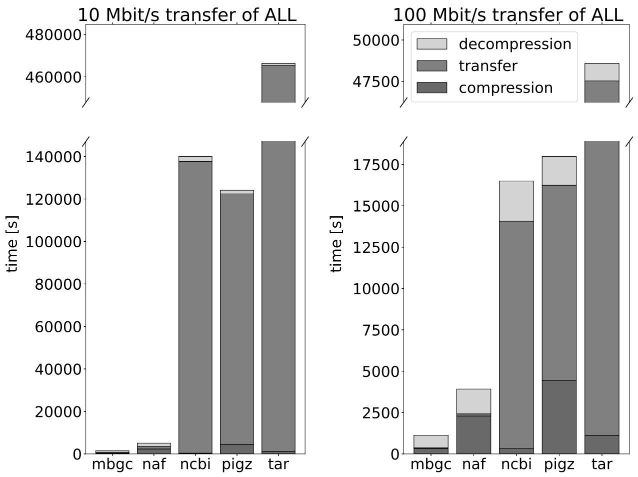

Figure 3. Total times of compressing, transferring and decompressing a collection of 168,311 genomes. The input in the MBGC and "ncbi" scenarios were gzipped FASTA files.

mitting raw FASTA files, where the compression phase is data tarring (merging), and the decompression phase is data untarring. The "ncbi" bars correspond to the gzip archives in the NCBI repository, where the compression time comprises only tarring the data (so, it is some lower bound estimation). Clearly, MBGC has a huge edge over the competitors, and only NAF comes relatively close with the faster network connection. Note also that even with a faster connection the gzip-based approaches (bars "ncbi" and "pigz", which is multithreaded gzip implementation) are more than an order of magnitude slower than MBGC. The gaps are generally greater with a slower connection, and for the transfer of 10 Mbit/s the advantage of MBGC over NAF is more than 3-fold and by a factor of almost 100 over "ncbi". Clearly (cf. also Fig. 2), the gains will be smaller with a smaller amount of data to download at a time, and also the impact of the compression and the decompression times grows with faster networks, making the results generally flatter. Similar figures for species collections of (all and 1024) genomes are included in the supplementary material.

We believe that the ideas behind MBGC can be adapted for a dedicated compressed index for bacterial genomes, allowing for fast pattern counting and reporting. Such an index could handle multi-genome mapping, i.e., mapping sequencing reads against multiple genomes in an efficient way (see, e.g., [18, 19, 20] and references therein). Compressed indexes for repetitive data have been a major research area in the string matching community in the past decade, but few solutions have been tested on a large scale, e.g., hundreds of gigabytes (one exception could be the MuGI index [21] which, for example, can maintain 1092 diploid human genomes in less than 8 GB of space, serving exact pattern queries of length 150 bp in below 80 µs on a commodity PC). Perhaps the major obstacle in running industry-scale experiments were construction costs, both in time and space, for many worst-case oriented indexing data structures. It could be argued that the level of similarity of bacterial genomes allows for relaxing the requirements and focusing on typical, not worst, cases, to obtain practical performance. Although the prospects are not fully clear, it is our opinion that the ideas of MBGC could be adapted to obtain a compressed index for bacterial collections combining high compression ratios, relatively low computational requirements of the construction and short access times.

Methods

There is significant redundancy in bacterial genomes which cannot be fully exploited using existing multiple genome compressors. The standard approach of finding repetitions between the currently processed genome and a reference genome (or possibly all previously processed genomes), and encoding them as LZ-phrases of the form (offset, length), is only moderately successful. We found out that many strings repeat as reverse-complements of corresponding strings from other genomes, a phenomenon known, but surprisingly rarely handled earlier (the COMRAD tool being an exception [22]).

It is also beneficial not to limit the reference to one, or a few, previous genome(s), but to allow finding matches occurring almost anywhere earlier. This, however, requires a potentially unbounded memory buffer. We mitigate this problem with building the reference string, i.e., a reservoir for possible matches, in an incremental manner, appending only blocks which are "new enough", that is, containing a relatively large fraction of DNA subsequences not seen before. This (general) approach, i.e., building "a dictionary of repeats", is known in the context of relative genome compression, see, e.g., [9] and [23].

As the key ideas of our solution, Multiple Bacteria Genome Compressor (MBGC), are already sketched, now we present the algorithm in detail.

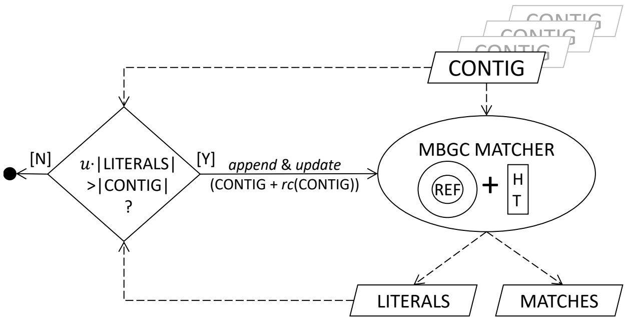

Figure 4. General scheme of the contig matching process with an emphasis on appending the reference buffer strategy

Basic algorithm

The main stages of MBGC compression are the contig matching process and the backend compression of matching products. Below we focus on explaining the former, essential stage.

The goal is to compress the sequence of genomes $G_1, \ldots, G_n$ in the FASTA format. The genomes consist of one or many contigs (by a contig, throughout the paper, we mean a sequence in the FASTA file). At the start the reference string *REF* is initialized with G_1 followed by $rc(G_1)$, where $rc(\cdot)$ stands for the reverse complement of the passed string. MBGC also stores a literal buffer, which is initialized with *REF* (but not its reverse complement). During the compression process, a hash table of fixed size (e.g., 2^{25} slots) is maintained, and the pairs of the form (h, pos) are inserted to it, where the positions *pos* are taken from *REF* accessed sparsely, with a stride of 16 symbols, and h are the hash values of corresponding k-mer seeds taken from the sampled positions. A collision on the hash h overwrites the previous value associated with it.

In the following steps the genomes $G_2, \ldots, G_n$ are taken one by one and LZ-matches of the form (*offset*, *length*), where *offset* is the position of *REF* where a match of length *length* begins, are sought. The contigs in the current genome are processed in their original order. If a match is not found for the given position (note that such a check takes a constant time, due to the extremely simple hash table organization), we move to the next position in the current contig, etc., and once we have a (tentative) match, we verify its k symbols and try to extend it maximally in both directions (with a restriction that matches cannot cross contig boundaries). The left extension of the current match is allowed to “swallow” the (whole) previous match(es). Surprisingly, this little idea is a powerful optimization trick which improves the compression ratio sometimes by more than 50% on our datasets, and is also moderately beneficial for the compression speed, as there are significantly fewer LZ-matches for further encoding. To make this effect even stronger (by up to a few percent), the next position just after a match is *decreased* by m (which is 16 by default). Using such a “skip margin” in some cases allows to find longer matches.

Finally, the symbols between matches are added to the literal buffer. At this point, we can define the strategy for augmenting the *REF* string depicted in Fig. 4. Once we are at the end of a contig, the portion of its symbols not covered with matches is checked; if it is large enough (exceeds $1/u$ of the contig length, where $u = 192$ by default), the *REF* string is appended with the contig and its reverse complement. The rationale is that contigs too similar to some parts of *REF* are almost completely redundant and thus do not contribute enough to facilitate compression, but increase the memory requirement. This design decision was indeed very successful, as in our test data the string *REF* together with the concatenated literals often took less than 2% of the input. If, however, the contigs to compress are not similar enough to the previous ones, the *REF* string grows quickly and may reach its limit, which depends on the number of genomes in the collection and the size of the first genome (details in the supplementary material). From this point on, the *REF* string works like a circular buffer, i.e., instead of being *appended* it is being overwritten from the starting position.

The resulting streams of match data (offsets, lengths), literals, header and filename data, and flags are compressed with LZMA and PPMd, using a well-known open-source software development kit (LZMA SDK).

For easier understanding of the MBGC internals, we created an example (see Fig. 5 in the supplementary material). Moreover, the last section of the supplementary material covers the details of backend compression of the streams resulting from the matching stage.

MBGC in the max mode

The description above corresponds to the single-threaded version of our algorithm. MBGC is, however, multi-threaded. The max MBGC’s mode (invoked as mbgc -c 3), with preference to the compression ratio rather than compression speed, does not use multithreading except for parallel input, backend compression and possibly gzip decompression; to understand such a design decision, see Fig. 1 and the related discussion.

We note that in the max mode the initialization of *REF* with G_1 is not required. Since matching is sequential, it can be started from G_1 instead of G_2 (even with an empty *REF* sequence). G_1 will be used to extend *REF* before matching the remaining genomes.

As multi-threaded matching implementation uses more memory, upper-bounding the reference buffer by 2^{32} bytes in the default mode helps to reduce the memory consumption during the compression of larger collections. On the other hand, in the max mode the buffer is allowed to grow up to 2^{40} bytes, which is beneficial for the compression ratio.

The last major difference between MBGC compression modes concerns backend compression. To optimize the performance in the default mode, the most time consuming of the resulting streams (i.e., match data and literals) are broken down into blocks and compressed in parallel, sacrificing however some compression ratio.

Multi-threading

MBGC makes use of the producer-consumer dataflow pattern. Assuming t worker threads, we have at most $t - 1$ producers and at least one consumer for the compression. The producers decompress and handle the input (gzip) files in parallel and store them in buffers (if the input file is uncompressed, the gzip decompression phase is simply skipped); each producer can handle up to 32 files (genomes) in its buffer. The consumer parses headers and contigs, and performs the actual compression (maintaining the hash table, finding LZ-matches, etc.). Once a producer fills up its buffer, it switches to compress the next unprocessed genome (entering a temporary consumer mode), which serves as a simple load balancing technique.

When a genome is fully encoded, the *REF* sequence is prolonged with the relevant contigs; updates to *REF* are performed in a critical section, preserving the original genome order (via a queuing mechanism). Let us explain this issue in more detail. We take care that the area of *REF* in which a worker looks for matches is not overwritten with newer contigs by other workers. To this end, when a worker W begins its job, it marks a guard position in the *REF* which prevents other workers from

overwriting *REF* beyond this position until *W* terminates processing a current genome. It might mean that some contigs cannot be written to *REF* and are thus ignored. Fortunately, in our experiments this detrimental effect hampers the compression ratio rather negligibly. When the buffer of a producer is not full, the producer again fills up its buffer by reading and processing the input data, and the consumer proceeds to compress new genomes.

Availability of source code and requirements

- Project name: MBGC: Multiple Bacteria Genome Compressor
- Project home page: https://github.com/kowallus/mbgc
- Operating system(s): Linux
- Programming language: C++
- Other requirements: C++14 standard or higher, cmake 3.4 or higher
- License: e.g. GNU GPL v3.0

Availability of supporting data and materials

The pathogen data sets supporting the results of this article are available in the *US National Center for Biotechnology Information* repository: https://www.ncbi.nlm.nih.gov/pathogens.

The yeast datasests (*S. cerevisiae* and *S. paradoxus)* genomes were taken from *Sanger Institute* repository: ftp://ftp.sanger.ac.uk/pub/users/dmc/yeast/latest/.

All benchmark data are available online: http://coach.kis.p.lodz.pl/mbgc-datasets/.

Declarations

List of abbreviations

1KGP: 1000 Genomes Project; BSC: Block Sorting Compressor; COMRAD: COMpression using RedundAncy of Dna; EOL: End-Of-Line; GCC: GNU Compiler Collection; GDC2: Genome Differential Compressor 2; HRCM: Hybrid Referential Compression Method; MBGC: Multiple Bacteria Genome Compressor; MuGI: Multiple Genome Index; NAF: Nucleotide Archival Format; NCBI: National Center for Biotechnology Information; LZ: Lempel-Ziv; LZMA: Lempel-Ziv-Markov chain-Algorithm; PPMd: Prediction by Partial Matching (variant by Dmitry Shkarin).

Ethical Approval

Not applicable.

Consent for publication

Not applicable.

Competing Interests

The authors declare that they have no competing interests.

Funding

This work was partially supported by the Faculty of Electrical, Electronic, Computer, and Control Engineering, Lodz University of Technology, as a statutory activity (both authors).

Author's Contributions

S.G. developed the overall conception and participated in the design of the work and in drafting the manuscript. T.M.K. participated in the design of the work, implemented the tool and conducted all major experiments, and participated in drafting the manuscript. Both authors reviewed and approved the final manuscript.

Acknowledgements

Not applicable.

References

1. Grumbach S, Tahi F. Compression of DNA sequences. In: Proc. Data Compression Conference, IEEE; 1993. p. 340–350.
2. Cao MD, et al. A simple statistical algorithm for biological sequence compression. In: Proc. Data Compression Conference, IEEE; 2007. p. 43–52.
3. Kryukov K, et al. Sequence Compression Benchmark (SCB) database—A comprehensive evaluation of reference-free compressors for FASTA-formatted sequences. GigaScience 2020;9(7):1–12.
4. Christley S, et al. Human genomes as email attachments. Bioinformatics 2009;25(2):274–275.
5. Pavlichin DS, et al. The human genome contracts again. Bioinformatics 2013;29(17):2199–2202.
6. Ochoa I, et al. iDoComp: a compression scheme for assembled genomes. Bioinformatics 2015;31(3):626–633.
7. Yao H, et al. HRCM: An Efficient Hybrid Referential Compression Method for Genomic Big Data. BioMed Research International 2019;2019:3108950.
8. Liu Y, et al. Allowing mutations in maximal matches boosts genome compression performance. Bioinformatics 2020;36(18):4675–4681.
9. Deorowicz S, Grabowski S. Robust relative compression of genomes with random access. Bioinformatics 2011;27(21):2979–2986.
10. Wandelt S, Leser U. FRESCO: Referential compression of highly similar sequences. IEEE/ACM Transactions on Computational Biology and Bioinformatics 2013;10(5):1275–1288.
11. Deorowicz S, et al. GDC 2: Compression of large collections of genomes. Sci Rep 2015;5:11565.
12. Silva M, et al. Efficient DNA sequence compression with neural networks. GigaScience 2020;9(11). https://doi.org/10.1093/gigascience/giaa119, giaa119.
13. Mohammed MH, et al. DELIMINATE—a fast and efficient method for loss-less compression of genomic sequences: Sequence analysis. Bioinformatics 2012;28(19):2527–2529. https://doi.org/10.1093/bioinformatics/bts467.
14. Kryukov K, et al. Nucleotide Archival Format (NAF) enables efficient lossless reference-free compression of DNA sequences. Bioinformatics 2019;35(19):3826–3828. https://doi.org/10.1093/bioinformatics/btz144.
15. Kuruppu S, et al. Relative Lempel-Ziv Compression of Genomes for Large-Scale Storage and Retrieval. In: Chávez E, Lonardi S, editors. String Processing and Information Retrieval - 17th International Symposium, SPIRE 2010, Los Cabos, Mexico, October 11-13, 2010. Proceedings, vol. 6393 of Lecture Notes in Computer Science Springer; 2010. p. 201–206. https://doi.org/10.1007/978-3-642-16321-0_20.
16. Belazzougui D, et al. Block Trees. Journal of Computer and System Sciences 2021;117:1–22.

Table 1. Compression results – collections of 1024 genomes.

	HRCM	BSC -p -b2047	7z -md4g	zstd -3 -long=31	NAF -3 -long=31	NAF -19 -long=31	MBGC default	MBGC max
				C. jejuni (1.78 GB)				
ratio	15.0	40.2	(1)78.5	31.2	43.0	54.2	(3)62.2	(2)73.1
ctime	196.3	38.0	1064.5	(2)4.0	(3)9.8	136.2	(1)3.7	12.4
dtime	27.0	10.6	(3)3.0	(1)1.5	4.4	4.4	(2)2.8	3.7
cmem	(2)1.76	9.00	18.00	1.92	1.90	2.24	(3)1.78	(1)1.19
dmem	(1)0.28	8.81	1.79	1.76	1.76	1.76	(3)1.45	(2)1.34
				E. coli (4.87 GB)				
ratio	158.2	166.9	377.5	357.8	495.2	(3)530.0	(2)1406.1	(1)1452.3
ctime	308.3	127.4	2228.7	(3)10.0	20.8	57.5	(1)3.0	(2)7.3
dtime	28.9	42.4	5.6	(3)2.9	12.6	11.9	(1)1.5	(1)1.5
cmem	(2)1.88	24.04	45.98	2.31	(3)2.30	2.65	2.55	(1)1.22
dmem	(1)0.66	21.48	4.82	2.15	2.21	2.21	(2)1.17	(2)1.17
				L. monocyt. (3.09 GB)				
ratio	39.3	70.4	(2)268.8	82.9	131.1	163.3	(3)243.0	(1)274.9
ctime	225.5	123.5	1838.1	(2)4.5	13.8	102.8	(1)3.2	(3)9.3
dtime	26.0	36.7	4.0	(1)2.1	7.6	7.6	(1)2.1	(3)2.4
cmem	(2)1.79	15.29	31.82	2.31	2.30	2.63	(3)2.27	(1)1.25
dmem	(1)0.30	15.30	3.07	2.15	2.15	2.15	(3)1.55	(2)1.41
				S. enterica (5.2 GB)				
ratio	268.4	130.0	472.4	427.9	547.5	(3)607.1	(2)1329.8	(1)1356.8
ctime	308.2	130.6	2350.3	(2)6.1	20.4	36.1	(1)3.1	(3)7.3
dtime	28.2	43.0	5.9	(3)3.0	12.8	12.9	(1)1.7	(2)1.9
cmem	(2)1.91	25.67	49.45	2.31	(3)2.30	2.64	2.62	(1)1.22
dmem	(1)0.48	25.68	5.15	2.15	2.17	2.17	(3)1.27	(2)1.26

The rows "ratio" show the ratio of the input to the output size. Compress / decompress times (as "ctime" / "dtime") are given in seconds, memory usages ("cmem" / "dmem") given in GB (G = 10^9). The best three results in a row are marked with a number in parentheses. HRCM is single-threaded (except for the latter phase where it invokes 7zip), BSC uses 12 threads, 7zip (up to) 6 threads, zstd 14 threads and MBGC 8 threads.

17. Rahn R, et al. Journaled string tree–a scalable data structure for analyzing thousands of similar genomes on your laptop. Bioinformatics 2014;30(24):3499–3505. https://doi.org/10.1093/bioinformatics/btu438.
18. Gagie T, Puglisi S. Searching and Indexing Genomic Databases via Kernelization. Front Bioeng Biotechnol 2015;3.
19. Kuhnle A, et al. Efficient Construction of a Complete Index for Pan-Genomics Read Alignment. J Comput Biol 2020;27(4):500–513.
20. Sherman RM, Salzberg SL. Pan-genomics in the human genome era. Nature Reviews Genetics 2020;21:243–254.
21. Danek A, et al. Indexes of Large Genome Collections on a PC. PLoS ONE 2014;9(10):1–12.
22. Kuruppu S, et al. Iterative Dictionary Construction for Compression of Large DNA Data Sets. IEEE ACM Trans Comput Biol Bioinform 2012;9(1):137–149.
23. Kuruppu S, et al. Reference Sequence Construction for Relative Compression of Genomes. In: SPIRE, vol. 7024 of Lecture Notes in Computer Science Springer; 2011. p. 420–425.

Table 2. Compression results – large species collections.

	BSC -p -b2047	7z -md4g	zstd -3 -long=31	NAF -3 -long=31	NAF -19 -long=31	MBGC default	MBGC max
C. jejuni (55,627 genomes, totalling 98.38 GB)							
ratio	69.7	164.9	74.9	137.1	(3)176.6	(2)412.5	(1)450.6
ctime	969.2	22881.0	(2)211.9	489.5	2570.7	(1)92.7	(3)400.8
dtime	241.3	129.3	(3)127.3	280.3	250.9	(1)78.5	(2)102.3
cmem	128.84	122.54	(2)2.32	(1)2.31	(3)2.66	8.78	7.22
dmem	129.21	34.59	(1)2.15	(3)2.70	(2)2.69	5.62	5.02
E. coli (22,523 genomes, totalling 114.67 GB)							
ratio	93.1	342.7	242.8	(3)460.9	458.5	(2)1747.4	(1)2051.6
ctime	1195.1	30086.0	(2)165.5	446.3	1313.5	(1)65.0	(3)216.1
dtime	291.6	(3)160.2	164.7	296.0	288.7	(2)83.3	(1)78.4
cmem	128.84	122.48	(1)2.31	(1)2.31	(3)2.66	9.66	10.87
dmem	129.17	34.48	(1)2.15	(3)3.10	(3)3.10	3.25	(2)2.66
L. monocyt. (36,448 genomes, totalling 112.00 GB)							
ratio	90.2	(3)328.2	137.3	274.9	323.9	(2)1086.9	(1)1160.2
ctime	1166.4	28065.0	(2)162.7	450.1	1805.7	(1)68.0	(3)263.8
dtime	287.6	(3)162.0	184.5	286.4	279.0	(1)84.2	(2)97.2
cmem	128.84	122.48	(2)2.32	(1)2.31	(3)2.66	7.85	7.03
dmem	129.13	34.48	(1)2.15	(2)2.39	(2)2.39	3.82	3.30
S. enterica (53,713 genomes, totalling 262.21 GB)							
ratio	156.7	695.5	606.0	1205.7	(3)1312.0	(2)5786.0	(1)5881.1
ctime	2655.8	61182.0	(2)290.2	909.9	1423.9	(1)127.3	(3)342.1
dtime	618.0	440.4	(3)396.5	659.9	662.3	(2)270.3	(1)262.0
cmem	128.84	122.46	(1)2.31	(1)2.31	(3)2.66	11.31	10.48
dmem	129.06	34.43	(1)2.15	2.80	2.79	(3)2.51	(2)2.36

The rows "ratio" show the ratio of the input to the output size. Compress / decompress times (as "ctime" / "dtime") are given in seconds, memory usages ("cmem" / "dmem") given in GB (G = 10^9). The best three results in a row are marked with a number in parentheses. BSC uses 12 threads, 7zip (up to) 6 threads, zstd 14 threads and MBGC 8 threads.

Table 3. Compression results – mixed species collections.

	BSC -p -b2047	7z -md=4g	zstd -3 -long=31	NAF -3 -long=31	NAF -19 -long=31	MBGC default	MBGC max
168,311 genomes (587.26 GB)							
ratio	105.3	354.5	193.6	369.0	(3)434.0	(2)1266.6	(1)1411.4
ctime	5824.0	140902.0	(2)846.1	2287.3	7100.0	(1)370.9	(3)1271.6
dtime	1379.7	(3)880.8	970.2	1506.2	1499.7	(1)749.2	(2)757.2
cmem	128.84	122.54	(1)2.31	(1)2.31	(3)2.66	23.06	15.93
dmem	129.22	34.59	(1)2.15	(2)4.51	(3)4.53	8.70	10.73
4×1024 genomes (14.94 GB)							
ratio	88.2	(2)239.2	124.5	177.1	214.6	(3)220.8	(1)351.5
ctime	189.4	4124.0	(2)24.4	62.6	334.6	(1)13.4	(3)36.5
dtime	56.7	15.5	(3)9.9	37.4	37.3	(2)8.9	(1)8.6
cmem	73.79	122.48	(2)2.31	(2)2.31	2.65	4.69	(1)2.22
dmem	73.82	14.83	(1)2.15	(2)2.24	(2)2.24	3.24	2.83

The rows "ratio" show the ratio of the input to the output size. Compress / decompress times (as "ctime" / "dtime") are given in seconds, memory usages ("cmem" / "dmem") given in GB (G = 10^9). The best three results in a row are marked with a number in parentheses. BSC uses 12 threads, 7zip (up to) 6 threads, zstd 14 threads and MBGC 8 threads.

Table 4. Compression results on non-bacterial genome collections, *S. cerevisiae* and *S. paradoxus*.

	S. cerevisiae (39 genomes, 486 MB)					S. paradoxus (36 genomes, 429 MB)				
	ratio	ctime	dtime	cmem	dmem	ratio	ctime	dtime	cmem	dmem
GDC 2	(1)109.8	4.12	(2)0.57	(1)0.52	(2)0.14	(2)80.7	21.92	(3)0.82	(2)0.51	(2)0.17
HRCM	78.8	7.13	2.85	1.18	(1)0.06	52.6	8.30	3.18	1.18	(1)0.07
BSC -p -b2047	52.9	10.76	2.65	2.49	2.44	33.8	9.59	2.59	2.21	2.16
7z -md4g	(2)100.6	316.38	(3)0.73	4.92	0.50	(1)83.9	295.95	(2)0.69	4.41	0.44
zstd -3 –long=31	45.8	(1)0.98	(1)0.43	(2)0.54	(3)0.49	30.5	(1)0.87	(1)0.40	(1)0.49	(3)0.43
NAF -3 –long=31	67.0	(3)2.80	1.02	(3)0.63	(3)0.49	43.2	(3)2.61	0.92	(3)0.57	(3)0.43
NAF -19 –long=31	77.0	30.54	1.03	0.97	(3)0.49	43.2	33.54	0.93	0.91	(3)0.43
MBGC default	87.3	(2)1.68	0.82	1.93	0.80	49.1	(2)2.08	1.07	1.84	0.73
MBGC max	(3)90.6	3.72	1.04	1.50	0.87	(3)61.5	3.58	1.24	1.40	0.70

The columns “ratio” show the ratio of the input to the output size. Compress / decompress times (as “ctime” / “dtime”) are given in seconds, memory usages (“cmem” / “dmem”) given in GB (G = 10^9). The best three results in a column are marked with a number in parentheses. HRCM is single-threaded (except for the latter phase where it invokes 7zip), BSC uses 12 threads, 7zip (up to) 6 threads, zstd 14 threads and MBGC 8 threads.

Click here to access/download
Supplementary Material
MBGC_supplementary.pdf

cover letter Click here to access/download;Personal Cover;cover_letter.txt

Dear Editor,

We submit a revised version of our manuscript:

"MBGC: Multiple Bacteria Genome Compressor"
by
Szymon Grabowski and Tomasz M. Kowalski.

We thank Reviewer #1 for his comments. We address them in the provided responses.

The manuscript is (slightly) modified in a few places, marked in color. We also prepared a new mbgc release (v1.2.1) to include the Reviewer's suggestions concerning the menu.

Yours sincerely,
Szymon Grabowski (on behalf of both co-authors)
