# Supplementary material for: MBGC: Multiple Bacteria Genome Compressor
Source: Gigascience. 2022 Jan 27;11:giab099. doi: 10.1093/gigascience/giab099 (PMC8848312; doi:10.1093/gigascience/giab099)
Supplement: giab099_Supplemental_File [file giab099_supplemental_file.pdf]

Supplementary Material to:
“MBGC: Multiple Bacteria Genome Compressor”

Szymon Grabowski and Tomasz M. Kowalski

December 2021

1 Datasets

All the bacterial datasets used in the experiment are taken from the NCBI Pathogen Detection project (https://www.ncbi.nlm.nih.gov/pathogens) and belong to the following species:

- Campylobacter jejuni
  https://www.ncbi.nlm.nih.gov/pathogens/isolates/#taxgroup_name%3A%22Campylobacter%20jejuni%22
  (55,627 genomes, totalling 27,755 MB in gzip),
- E.coli and Shigella
  https://www.ncbi.nlm.nih.gov/pathogens/isolates/#taxgroup_name%3A%22E.coli%20and%20Shigella%22
  (22,523 genomes, totalling 33,708 MB in gzip),
- Listeria monocytogenes
  https://www.ncbi.nlm.nih.gov/pathogens/isolates/#taxgroup_name%3A%22Listeria%20monocytogenes%22
  (36,448 genomes, totalling 32,775 MB in gzip),
- Salmonella enterica:
  https://www.ncbi.nlm.nih.gov/pathogens/isolates/#taxgroup_name%3A%22Salmonella%20enterica%22
  (53,713 genomes, totalling 77,239 MB in gzip).

The full list of the bacterial genomes (URLs) used in our experiments is available at https://github.com/kowallus/mbgc/releases/download/v1.2.1/tested_samples_lists.7z

We also use the following yeast collections (downloaded on Nov. 25, 2020):

- Saccharomyces cerevisiae
  ftp://ftp.sanger.ac.uk/pub/users/dmc/yeast/latest/cere_assemblies.tgz
  (39 genomes, totalling 494 MB in FASTA),
- Saccharomyces paradoxus
  ftp://ftp.sanger.ac.uk/pub/users/dmc/yeast/latest/para_assemblies.tgz
  (36 genomes, totalling 436 MB in FASTA).

For experiments presented in Table 1 and Table 2 we use the following small H. sapiens collection (assembled by the International Human Genome Project between 2003–2009):

- H. sapiens hg16
  ftp://hgdownload.soe.ucsc.edu/goldenPath/hg16/chromosomes
  (25 chromosomes, totalling 3,132 MB in FASTA),
- H. sapiens hg17
  ftp://hgdownload.soe.ucsc.edu/goldenPath/hg17/chromosomes
  (25 chromosomes, totalling 3,138 MB in FASTA),
- H. sapiens hg18
  ftp://hgdownload.soe.ucsc.edu/goldenPath/hg18/chromosomes
  (25 chromosomes, totalling 3,142 MB in FASTA),

- H. sapiens hg19
  `ftp://hgdownload.soe.ucsc.edu/goldenPath/hg19/chromosomes`
  (25 chromosomes, totalling 3,157 MB in FASTA).

Finally, in single FASTA file mode experiments (Tables 3–5) we use the following RNA, protein and DNA datasets (downloaded on Oct. 7, 2021):

- SILVA 132 LSURef
  `https://ftp.arb-silva.de/release_132/Exports/SILVA_132_LSURef_tax_silva.fasta.gz`
  (198,843 RNA sequences, totalling 610 MB in FASTA),
- SILVA 132 SSURef
  `https://ftp.arb-silva.de/release_132/Exports/SILVA_132_SSURef_tax_silva.fasta.gz`
  (2,090,668 RNA sequences, totalling 3,282 MB in FASTA),
- H. sapiens GRCh38 peptides all
  `ftp://ftp.ensembl.org/pub/release-96/fasta/homo_sapiens/pep/Homo_sapiens.GRCh38.pep.all.fa.gz`
  (109,914 protein sequences, totalling 73 MB in FASTA),
- PDB
  `ftp://ftp.ncbi.nih.gov/blast/db/FASTA/pdbaa.gz`
  (132,163 protein sequences, totalling 98 MB in FASTA),
- UniProtKB Reviewed (Swiss-Prot)
  `ftp://ftp.uniprot.org/pub/databases/uniprot/current_release/knowledgebase/complete/uniprot_sprot.fasta.gz`
  (562,254 protein sequences, totalling 98 MB in FASTA),
- Influenza
  `ftp://ftp.ncbi.nih.gov/genomes/INFLUENZA/influenza.fna.gz`
  (817,587 DNA sequences, totalling 1,429 MB in FASTA),
- Mitochondrion 1
  `ftp://ftp.ncbi.nlm.nih.gov/refseq/release/mitochondrion/mitochondrion.1.1.genomic.fna.gz`
  (7,819 DNA sequences, totalling 229 MB in FASTA).

For convenience, all collections used in our experiments are made available in various compressed formats: `http://coach.kis.p.lodz.pl/mbgc-datasets`

2 Tested programs

The following programs, with the set parameters (e.g., for number of threads set to 12), were used in our experiments, with their results presented either in the main paper or in Section 4 of Supplementary Material.

HRCM (Hybrid Referential Compression Method)
(version from 2020-Oct-12, `https://github.com/haicy/HRCM/`):

collection of FASTA files compression:

```
hrcm compress -r <ref-file> -f <sequences-list-file>
```

collection of FASTA files decompression:

```
hrcm decompress -r <ref-file> -f <sequences-list-file>
```

GDC 2 (Genome Differential Compressor 2)
(version from 2020-Jun-24, `https://github.com/refresh-bio/GDC2`):

collection of FASTA files compression:

```
gdc2 c <archive-file> @<sequences-list-file>
```

collection of FASTA files decompression:

```
gdc2 d <archive-file>
```

BSC (Block Sorting Compressor)
(version 3.2.3 from 2021-Sep-30, `https://github.com/IlyaGrebnov/libbsc/releases/tag/3.2.3`):

single file (e.g., FASTA or tar) compression:

```
bsc e <in-file> -o <archive-file> -p -b2047
```

single file (e.g., FASTA or tar) decompression:

```
bsc d <archive-file> <out-file>
```

The number of worker threads used by BSC was OS-limited (up to 12).

7-Zip (x64)
(version 21.03 beta from 2021-Jul-20, `https://www.7-zip.org/`):

single file (e.g., FASTA or tar) compression:

```
7zz a -t7z -m0=lzma2 -mmt6 -mx=9 -md=4g <archive-file> <in-file>
```

single file (e.g., FASTA or tar) decompression:

```
7zz x -o<out-path> <archive-file>
```

zstd (64-bit)
(version 1.5.0 from 2021-May-14, `https://facebook.github.io/zstd/`):

single file (e.g., FASTA or tar) compression (level -3):

```
zstd -3 --long=31 -T14 <in-file> -o <archive-file>
```

single file (e.g., FASTA or tar) compression (level -19):

```
zstd -19 --long=31 -T14 <in-file> -o <archive-file>
```

single file (e.g., FASTA or tar) decompression:

```
zstd -d --long=31 <archive-file> -o <out-file>
```

NAF (Nucleotide Archival Format)
(version 1.3.0 from 2021-May-17, `https://github.com/KirillKryukov/naf`):

single FASTA file compression (level -3):

```
ennaf <in-file> -3 --long 31 --text -o <archive-file>
```

single FASTA file compression (level -19):

```
ennaf <in-file> -19 --long 31 --text -o <archive-file>
```

single FASTA file decompression:

```
unnaf <archive-file> -o <out-file>
```

DELIMINATE
(version 1.3c from 2021-May-17, received from its Authors):

single FASTA file compression:
`delim a` *<in-file>*

single FASTA file decompression:
`delim e` *<archive-file>*

mbgc
(version 1.2.1 from 2021-Dec-02, `https://github.com/kowallus/mbgc/releases/tag/v1.2.1`):

collection of FASTA files compression (default level, -c 1):
`mbgc -c 1` *<sequences-list-file>* *<archive-file>*

collection of FASTA files compression (max level, -c 3):
`mbgc -c 3` *<sequences-list-file>* *<archive-file>*

single FASTA file compression (default level, -c 1):
`mbgc -c 1 -i` *<in-file>* *<archive-file>*

single FASTA file compression (max level, -c 3):
`mbgc -c 3 -i` *<in-file>* *<archive-file>*

decompression:
`mbgc -d` *<archive-file>* *<out-path>*

Note that the default MBGC compression (-c 1) can be run with a simplified syntax:
`mbgc` *<sequences-list-file>* *<archive-file>*

2.1 Conversion of FASTA files collection to a single file

NAF and DELIMINATE tools directly do not support compression of a FASTA files collection. Working with multiple FASTA files is possible by combining them into a single Multi-Multi-FASTA file using mumu.pl script (script and more information can be found at `https://github.com/KirillKryukov/mumu`).

Packing multiple FASTA files into a Multi-Multi-FASTA file:
`mumu.pl --stdin <` *<sequences-list-file>* `>` *<Multi-Multi-FASTA-file>*

Unpacking a Multi-Multi-FASTA file:
`mumu.pl --unpack --dir` *<out-path>* `<` *<Multi-Multi-FASTA-file>*

We have omitted the time of packing and unpacking in the results of our experiments.

3 Test setup

All experiments were run on a Linux (Debian) machine equipped with a 14-core Intel Core i9-10940X 3.3 GHz CPU, 128 GB of DDR4-RAM (CL 16, clocked at 2666 MHz) and a fast SSD (ADATA 2 TB M.2 PCIe NVMe XPG SX8200 Pro, sequential read speed 3500 MB/s, sequential write speed 3000 MB/s). MBGC is written in C++14 and was compiled with gcc 10.2.1 -O3 -DNDEBUG -mavx2. The disk cache was flushed between runs, to have raw reads of the input files from the disk.

Table 1: Compression results – small collections of human genomes (chromosomes separated).

	hg16 + ... + hg19 all chromosomes				
	ratio	ctime	dtime	cmem	dmem
HRCM	14.35	381.9	7.21	2.52	(3)0.97
GDC 2	13.98	1421.6	(4)2.94	3.55	(2)0.88
DELIMINATE	6.48	657.2	38.79	(1)0.72	(1)0.07
BSC -p -b2047	14.87	287.0	5.88	5.06	4.95
7z -md4g	(1)17.92	11653.1	2.97	9.98	1.05
zstd -3 –long=31	13.17	(1)14.8	(1)0.95	(2)1.09	(4)0.99
NAF -3 –long=31	13.24	(2)76.9	(2)2.63	(3)1.13	(4)0.99
NAF -19 –long=31	(4)16.46	2068.3	(3)2.76	(4)1.47	(4)0.99
MBGC default	(3)17.06	(3)126.8	7.24	4.20	3.31
MBGC max	(2)17.71	(4)271.6	11.44	9.77	3.48

The column "ratio" shows the ratio of the input to the output size. Compress / decompress times (as "ctime" / "dtime") given in seconds, memory usages ("cmem" / "dmem") given in GB (G = 10^9). The best four results are marked with a number in parentheses. NAF is single-threaded, DELIMINATE uses 2 threads, HRCM is single-threaded (except for the latter phase where it invokes 7zip), BSC uses 12 threads, 7zip (up to) 6 threads, zstd 14 threads and MBGC 8 threads.

4 Additional tables and figures

MBGC allows extracting only selected files (matching the given pattern), but decompression time depends on the location of the decompressed file in the archive. MBGC cannot decompress, e.g., a single genome from a compressed collection without prior processing (in memory) the previous genomes. In other words, there is no random access to a compressed collection; extracting a genome close to the beginning of the archive takes however less time than extracting one close to its end. Extracting a single file close to the beginning (resp. end) of the archive requires 20.5% (resp. 68.3%) of the total decompression time on average, where in the experiment four 1k genome collections and four whole-species genome collections were used. On the whole collections only, the time gets better and drops to 9.0% (resp. 39.7%). For the whole collection of *S. enterica* extracting the first file is possible only in 1.3% of the total compression time, whereas in case of *C. jejuni* the same task requires 20.1% of the total compression time. We note that extracting, e.g., the (single) last genome in the archive is significantly faster than extracting the whole archive due to reduced I/O. It is clear that dividing the input data into blocks of several genomes each poses a tradeoff between compression ratio and single genome extraction time (small blocks improve the extraction time but hamper the compression), but we have not examined this aspect carefully, possibly leaving it for a future work. We also point out that the current version of MBGC does not allow to update an archive with new genomes.

We ran the experiments on human genomes (each genome consists of multiple input files, one per chromosome) in two settings. In Table 1 multiple archives were created, arranged by chromosome. More precisely, a given compressor was to create an archive with four chr1 sequences, from the genomes hg16, ..., hg19, similarly an archive with four chr2 sequences, etc., and the sizes (and (de)compression times) were summed, and the presented (de)compression memory is the peak memory usage during such a procedure. In Table 2 the whole genomes are stored one by one, and we also present compression results for prefixes of our collection (e.g., the top five lines present the compression ratio, compression time, decompression time, compression memory and decompression memory for hg16 only).

In the former experiment (Table 1) in compression ratio MBGC gives way only to 7z (the difference is up to 5%), but MBGC is close to two orders of magnitude faster in compression (slower in decompression, though). One of the stronger tools in this comparison is NAF -19 -long=31, compression ratio only a few percent behind 7z and MBGC in the compression ratio, yet over 16 times slower than MBGC default in the compression. Definitely the fastest (and also among the most memory frugal) compressor and decompressor is zstd -3 -long=31, but its compression ratio is by around 25% worse than MBGC's. GDC 2 could spread its wings on larger collections; its reference genome compression is not quite on par with, e.g., MBGC, but its relative compression in human data is significantly stronger. The latter experiment (Table 2) shows general-purpose compressors and MBGC encoding multiple whole human genomes sequentially. General-purpose compressors cannot improve their compression ratio, presumably due to memory limitations, and their compression ratio remains on the level between 3.56 (zstd -3) and 6.47 (7z). The good result of 7z can be attributed to its large maximum buffer (in recent versions of the tool), which is of size 4 GB. MBGC in the default mode raised its compression ratio from 4.90 (one genome only) to 10.20 (four genomes). Even a larger improvement was observed for MBGC max: from 4.90 (one genome) to 18.60 (four genomes). This can be explained by the positive impact of a large reference buffer, exceeding 2^{32}

Table 2: Compression results – small collections of human genomes (ordered by genomes).

	BSC -p -b2047	DELI-MINATE	7z -md4g	zstd -3 –long=31	NAF -3 –long=31	NAF -19 –long=31	MBGC default	MBGC max
				hg16 (3.13 GB)				
ratio	$^{(1)}$5.12	$^{(2)}$5.00	4.89	3.56	3.56	4.48	$^{(3)}$4.90	$^{(3)}$4.90
ctime	$^{(3)}$161.5	193.6	4426.0	$^{(1)}$5.0	$^{(2)}$30.6	1866.7	162.7	319.1
dtime	67.9	123.6	28.9	$^{(1)}$4.8	$^{(3)}$9.9	$^{(2)}$9.4	65.1	115.8
cmem	15.36	$^{(1)}$0.72	31.94	$^{(3)}$2.32	$^{(2)}$2.29	2.63	10.40	26.41
dmem	10.75	$^{(1)}$0.07	3.72	$^{(2)}$2.15	$^{(2)}$2.15	$^{(2)}$2.15	10.17	12.12
				hg16 + hg17 (6.27 GB)				
ratio	5.13	5.01	$^{(3)}$6.13	3.56	3.56	4.48	$^{(2)}$7.50	$^{(1)}$9.80
ctime	$^{(3)}$169.3	400.5	6834.0	$^{(1)}$10.1	$^{(2)}$61.0	3751.0	174.4	334.8
dtime	80.3	249.3	31.2	$^{(1)}$9.7	$^{(3)}$19.1	$^{(2)}$18.2	86.2	119.5
cmem	30.74	$^{(1)}$0.72	60.66	$^{(3)}$2.32	$^{(2)}$2.29	2.63	14.04	28.17
dmem	30.75	$^{(1)}$0.07	7.19	$^{(2)}$2.15	$^{(2)}$2.15	$^{(2)}$2.15	14.07	17.05
				hg16 + hg17 + hg18 (9.41 GB)				
ratio	5.13	5.01	$^{(3)}$6.64	3.56	3.56	4.48	$^{(2)}$8.60	$^{(1)}$14.40
ctime	$^{(3)}$188.6	617.9	7331.0	$^{(1)}$18.7	$^{(2)}$91.3	5634.0	206.9	364.5
dtime	84.1	373.3	35.1	$^{(1)}$13.8	$^{(3)}$28.6	$^{(2)}$26.9	112.9	125.5
cmem	46.15	$^{(1)}$0.72	89.85	$^{(3)}$2.32	$^{(2)}$2.29	2.63	17.89	34.25
dmem	42.95	$^{(1)}$0.07	10.67	$^{(2)}$2.15	$^{(2)}$2.15	$^{(2)}$2.15	18.22	26.30
				hg16 + hg17 + hg18 + hg19 (12.57 GB)				
ratio	5.13	5.01	$^{(3)}$6.47	3.56	3.56	4.48	$^{(2)}$10.20	$^{(1)}$18.60
ctime	$^{(3)}$204.8	803.9	7535.0	$^{(1)}$20.1	$^{(2)}$124.7	7536.0	215.2	403.1
dtime	87.4	495.1	$^{(3)}$37.4	$^{(1)}$18.7	37.5	$^{(2)}$36.2	131.7	134.3
cmem	61.62	$^{(1)}$0.72	121.46	$^{(3)}$2.32	$^{(2)}$2.29	2.63	20.82	40.52
dmem	61.65	$^{(1)}$0.07	14.30	$^{(2)}$2.15	$^{(2)}$2.15	$^{(2)}$2.15	21.90	35.56

The rows "ratio" show the ratio of the input to the output size. Compress / decompress times (as "ctime" / "dtime") are given in seconds, memory usages ("cmem" / "dmem") given in GB ($G = 10^9$). The best three results are marked with a number in parentheses. NAF is single-threaded, DELIMINATE uses 2 threads, BSC uses 12 threads, 7zip (up to) 6 threads, zstd 14 threads and MBGC 8 threads.

Table 3: Compression results for single-file inputs (single Multi-Multi-FASTA); RNA data

	SILVA 132 LSURef (0.61 GB)					SILVA 132 SSURef (3.28 GB)				
	ratio	ctime	dtime	cmem	dmem	ratio	ctime	dtime	cmem	dmem
DELIMINATE	10.86	23.41	28.53	$^{(4)}$0.72	$^{(3)}$0.07	11.36	141.74	153.71	$^{(3)}$0.72	$^{(1)}$0.11
BSC -p -b2047	$^{(2)}$51.47	13.07	4.46	3.10	3.03	$^{(1)}$54.59	121.72	37.58	16.24	16.25
7z -md4g	$^{(1)}$52.22	129.84	1.16	6.51	0.62	$^{(2)}$42.08	839.49	6.54	33.52	3.33
zstd -3 –long=31	28.40	$^{(2)}$1.49	$^{(3)}$0.53	$^{(3)}$0.69	$^{(4)}$0.61	23.49	$^{(2)}$11.49	$^{(3)}$2.81	$^{(4)}$2.31	$^{(4)}$2.15
zstd -19 –long=31	$^{(3)}$47.65	19.69	$^{(2)}$0.52	1.46	$^{(4)}$0.61	$^{(3)}$41.89	114.50	$^{(2)}$2.57	3.73	$^{(4)}$2.15
NAF -3	15.74	$^{(1)}$1.34	$^{(4)}$0.54	$^{(1)}$0.02	$^{(1)}$0.03	13.77	$^{(1)}$7.15	3.13	$^{(1)}$0.03	$^{(2)}$0.30
NAF -3 –long=31	31.90	$^{(4)}$3.14	0.60	0.73	$^{(4)}$0.61	25.92	$^{(3)}$18.25	3.29	$^{(4)}$2.31	2.45
NAF -19	38.37	96.24	$^{(1)}$0.45	$^{(2)}$0.37	$^{(2)}$0.04	33.56	908.30	$^{(1)}$2.50	$^{(2)}$0.39	$^{(3)}$0.31
NAF -19 –long=31	41.49	45.72	0.57	1.07	$^{(4)}$0.61	$^{(4)}$34.97	441.03	$^{(4)}$3.10	2.66	2.45
MBGC default	36.00	$^{(3)}$2.90	2.56	1.40	0.68	31.40	$^{(4)}$19.62	16.07	5.51	2.47
MBGC max	$^{(4)}$45.60	6.33	2.63	1.23	0.97	34.10	52.89	17.02	5.41	4.92

The columns "ratio" show the ratio of the input to the output size. Compress / decompress times (as "ctime" / "dtime") given in seconds, memory usages ("cmem" / "dmem") given in GB ($G = 10^9$). The best four results are marked with a number in parentheses. NAF is single-threaded, DELIMINATE uses 2 threads, BSC uses 12 threads, 7zip (up to) 6 threads, zstd 14 threads and MBGC 8 threads.

bytes in this experiment, allowing to find more (or longer) matches. On the other hand, zstd -3 was clearly the fastest and also among the memory frugal compressors; its compression ratio, however, like NAF's, is hampered by the buffer, too small to fit a whole human genome. The least memory usage, for the compression and the decompression, was reached by DELIMINATE, whose performance according to the other criteria was moderate at best. MBGC in the default mode is almost twice faster in the compression than MBGC max, and also requires close to twice less memory, but considering the gap in the compression ratio the max mode should be a more practical choice, assuming of course that the test machine has enough RAM memory.

MBGC is able to compress a collection of sequences stored in a single file, via the `-i input.fasta` switch, which is useful if the input is stored in such form. Note that HRCM and GDC 2 cannot be run in this setting, as they require a separate reference dataset, i.e., a separate file. When compressing a single file, MBGC initializes the reference buffer with all sequences which start in the first 2^{21} bytes of the file. The single-file experiments (Tables 3–5) show, however, a

Table 4: Compression results for single-file inputs (single Multi-Multi-FASTA); protein data

	Homo sapiens GRCh38 peptides all (0.07 GB)					PDB (0.10 GB)					UniProtKB Reviewed (Swiss-Prot) (0.28 GB)				
	ratio	ctime	dtime	cmem	dmem	ratio	ctime	dtime	cmem	dmem	ratio	ctime	dtime	cmem	dmem
DELIMINATE	3.29	9.65	9.88	$^{(2)}$0.10	$^{(3)}$0.05	3.96	177.25	177.53	$^{(2)}$0.10	$^{(3)}$0.10	2.73	30.30	31.76	$^{(2)}$0.18	$^{(3)}$0.12
BSC -p -b2047	5.96	$^{(4)}$2.02	0.50	0.38	0.38	6.12	$^{(4)}$2.69	0.66	0.51	0.50	3.45	$^{(4)}$7.82	2.55	1.43	1.40
7z -md4g	3.98	13.59	0.80	0.80	0.09	$^{(1)}$7.39	27.55	0.55	1.02	0.12	$^{(1)}$4.77	142.92	2.41	3.04	0.34
zstd -3 –long=31	6.61	$^{(1)}$0.15	$^{(1)}$0.10	$^{(2)}$0.10	$^{(4)}$0.07	4.37	$^{(1)}$0.20	$^{(1)}$0.15	$^{(3)}$0.13	$^{(3)}$0.10	3.80	$^{(1)}$0.51	$^{(1)}$0.41	$^{(3)}$0.34	$^{(4)}$0.28
zstd -19 –long=31	7.70	19.24	$^{(3)}$0.11	0.26	$^{(4)}$0.07	$^{(4)}$6.58	29.46	$^{(1)}$0.15	0.29	$^{(3)}$0.10	$^{(3)}$4.58	39.21	$^{(1)}$0.41	0.80	$^{(4)}$0.28
NAF -3	6.87	$^{(2)}$0.30	$^{(3)}$0.11	$^{(1)}$0.02	$^{(1)}$0.04	3.87	$^{(2)}$0.57	$^{(3)}$0.16	$^{(1)}$0.02	$^{(1)}$0.07	4.12	$^{(2)}$1.59	$^{(4)}$0.43	$^{(1)}$0.02	$^{(1)}$0.09
NAF -3 –long=31	7.24	$^{(3)}$0.43	0.12	$^{(4)}$0.20	0.08	4.86	$^{(3)}$0.65	0.17	$^{(4)}$0.19	$^{(3)}$0.10	4.13	$^{(3)}$2.07	0.47	$^{(4)}$0.36	0.29
NAF -19	$^{(1)}$8.22	23.36	$^{(1)}$0.10	0.37	$^{(1)}$0.04	6.06	38.77	$^{(3)}$0.16	0.37	$^{(1)}$0.07	$^{(2)}$4.76	103.46	$^{(1)}$0.41	0.38	$^{(1)}$0.09
NAF -19 –long=31	$^{(4)}$7.81	21.74	0.14	0.54	0.08	6.47	39.08	0.19	0.53	$^{(3)}$0.10	$^{(4)}$4.55	104.81	0.54	0.71	0.29
MBGC default	$^{(3)}$7.90	3.23	3.04	0.62	0.64	$^{(3)}$6.60	4.70	4.67	0.65	0.64	4.00	22.92	25.64	1.40	1.56
MBGC max	$^{(2)}$8.10	5.04	4.40	0.37	0.42	$^{(2)}$6.70	6.83	5.92	0.53	0.57	4.00	44.12	38.18	1.12	1.24

The columns "ratio" show the ratio of the input to the output size. Compress / decompress times (as "ctime" / "dtime") given in seconds, memory usages ("cmem" / "dmem") given in GB ($G = 10^9$). The best four results are marked with a number in parentheses. NAF is single-threaded, DELIMINATE uses 2 threads, BSC uses 12 threads, 7zip (up to) 6 threads, zstd 14 threads and MBGC 8 threads.

Table 5: Compression results for single-file inputs (single Multi-Multi-FASTA); DNA data

	Influenza (1.43 GB)					Mitochondrion_1 (0.23 GB)				
	ratio	ctime	dtime	cmem	dmem	ratio	ctime	dtime	cmem	dmem
DELIMINATE	68.99	36.95	48.55	$^{(3)}$0.72	$^{(1)}$0.07	$^{(3)}$6.22	8.69	7.89	0.59	$^{(3)}$0.06
BSC -p -b2047	$^{(1)}$87.02	30.23	8.74	7.22	7.07	5.80	5.71	1.74	1.17	1.14
7z -md4g	69.77	243.89	2.28	14.86	1.44	$^{(1)}$7.88	184.54	1.40	2.32	0.26
zstd -3 –long=31	43.70	$^{(2)}$4.40	$^{(4)}$1.15	1.56	$^{(4)}$1.41	4.29	$^{(1)}$0.36	$^{(4)}$0.37	$^{(2)}$0.27	$^{(4)}$0.23
zstd -19 –long=31	$^{(3)}$73.89	39.76	$^{(3)}$1.10	2.77	$^{(4)}$1.41	$^{(2)}$6.59	61.71	$^{(3)}$0.36	0.62	$^{(4)}$0.23
NAF -3	35.94	$^{(1)}$2.19	$^{(2)}$1.09	$^{(1)}$0.02	$^{(2)}$0.13	3.85	$^{(2)}$1.06	$^{(2)}$0.35	$^{(1)}$0.01	$^{(1)}$0.00
NAF -3 –long=31	59.58	$^{(4)}$6.83	1.34	$^{(4)}$1.46	1.43	4.42	$^{(3)}$1.70	0.40	$^{(4)}$0.37	$^{(4)}$0.23
NAF -19	$^{(4)}$73.67	239.00	$^{(1)}$0.96	$^{(2)}$0.38	$^{(2)}$0.13	5.98	134.43	$^{(1)}$0.28	$^{(3)}$0.35	$^{(2)}$0.01
NAF -19 –long=31	$^{(2)}$74.74	125.54	1.39	1.81	1.43	$^{(4)}$6.16	114.57	0.39	0.71	$^{(4)}$0.23
MBGC default	44.50	$^{(3)}$6.59	6.02	3.15	1.63	5.70	$^{(4)}$4.86	3.44	1.12	0.79
MBGC max	52.60	14.44	5.77	2.59	2.28	6.00	15.27	5.48	1.09	0.85

The columns "ratio" show the ratio of the input to the output size. Compress / decompress times (as "ctime" / "dtime") given in seconds, memory usages ("cmem" / "dmem") given in GB ($G = 10^9$). The best four results are marked with a number in parentheses. NAF is single-threaded, DELIMINATE uses 2 threads, BSC uses 12 threads, 7zip (up to) 6 threads, zstd 14 threads and MBGC 8 threads.

weaker spot of MBGC, which is not among the tools with best ratios (those are, usually, 7z, BSC, and NAF or zstd in its strongest mode). On the other hand, MBGC belongs to the faster contenders in the compression; it is usually beaten by NAF in modes -3 and zstd in all modes, but those tools are not clearly superior in the compression ratio. Unfortunately, MBGC's decompression is relatively slow. We do not comment the obtained results in detail, as diverse collections are not MBGC's forte.

Fig. 1 and Fig. 2 augment the results from the main article, presenting the total time to transfer (download) the given genome collection, but this time focusing on individual species collections. Assuming constant network connection speed of 10 Mbit/s (resp. 100 Mbit/s), repacking gzip-stored data (e.g., in NCBI repository) before the transfer using MBGC would benefit the clients with the 106× (resp. 18×) faster download on average (including the time to decompress the archive on the receiver's side). Particular MBGC download gains are mainly due to the compression ratio. In relation to the ncbi bar, they range from 5.1× (C. jejuni 1k genome collection with 100 Mbit/s network speed) to 165.5× (S. enterica 1k genome collection with 10 Mbit/s network speed).

4.1 MBGC properties

In the main paper, we pointed out the importance of reverse-complemented matches for the compression ratio of bacterial genome collections. Here we only mention that RC-matches, not surprisingly, do not help on the collection of human genomes and yeast collections (i.e., *S. cerevisiae* and *S. paradoxus*).

We also tried rotating the input collection of 1024 genomes from the same species (by a random number from $[1, 1023]$), or randomly permuting it, before MGBC compression, and the compression results varied by a few and sometimes even by more than 10% in the compression ratio (the ratios for the rotated or permuted data were often, but not always, worse than with the original file order), while the compression speed was more or less proportional to the ratio, i.e., worse compression was also slower.

As it is often the case in bioinformatics, dealing with very large data, the I/O speed has a significant impact on the overall performance. To this end, we note that *cached* read of the data (i.e., without flushing the disk buffers, which is

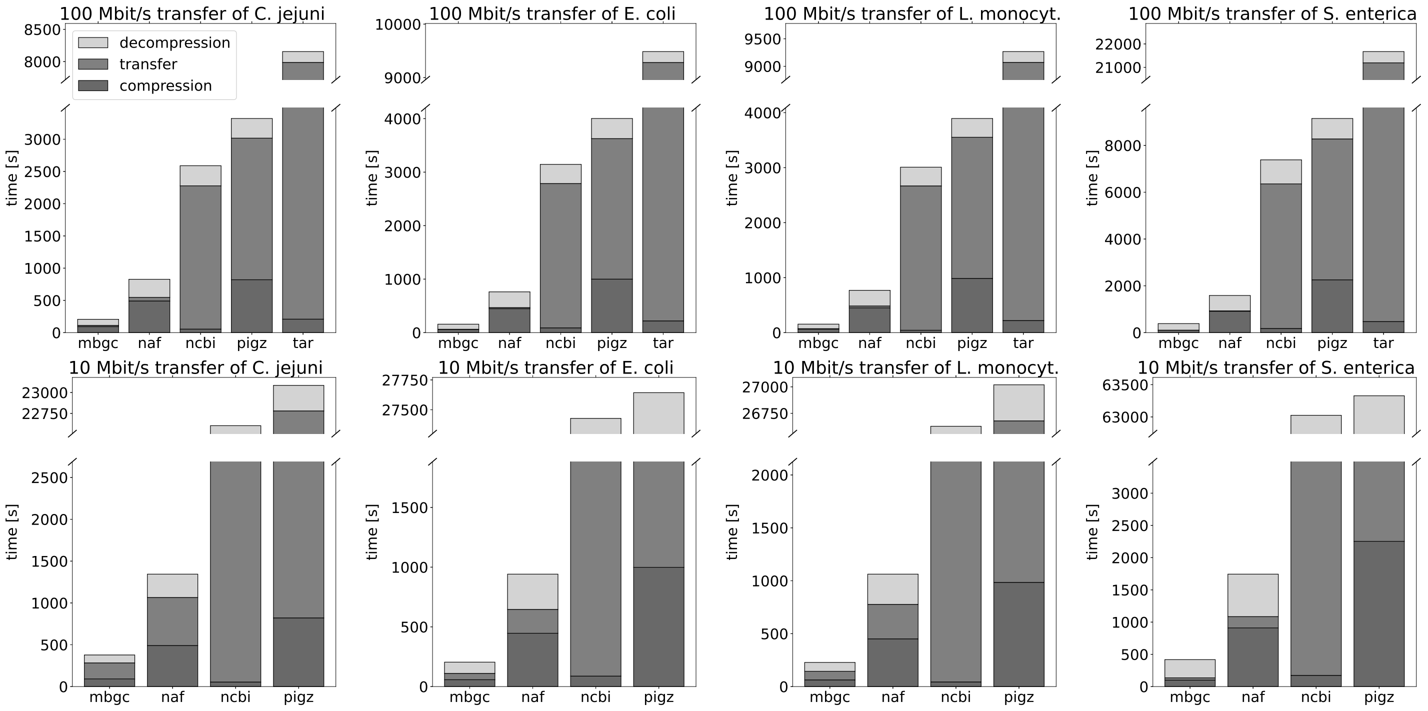

Figure 1: Total times of compressing, transferring and decompressing large collections of genomes, with network connection speed of 10 Mbit/s (bottom figures) and 100 Mbit/s (top figures)

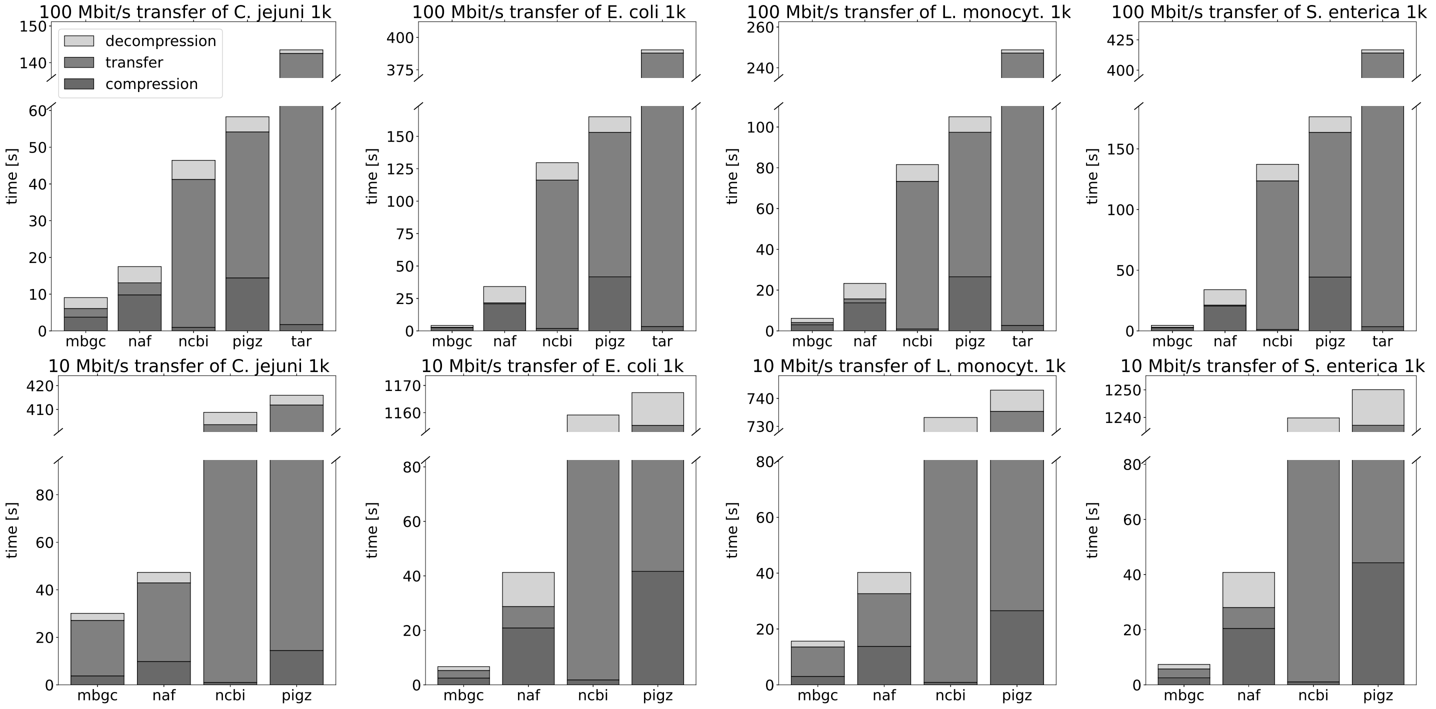

Figure 2: Total times of compressing, transferring and decompressing collections of 1k genomes, with network connection speed of 10 Mbit/s (bottom figures) and 100 Mbit/s (top figures)

standardly performed in our tests) makes MBGC default about 1.2 times faster in the compression, assuming that input is not much larger than the available RAM memory. It means that replacing our SSD with an (even) faster one would also have a similar effect. This observation corresponds to the uncompressed FASTA input; we have not tried to measure

such an effect with gzipped input.

4.2 Other tools

We have to admit we tried to compare our software also with other tools for (bacterial or not) genome collection compression: GDC 2, memRGC, iDoComp and MtGC, as well as with tools for single and multiple genome compression: GeCo3, Leon and MFCompress. The experiments were not successful though. GDC 2 refused to compress due to uneven number of contigs in the input files. We made it work by prior concatenation of all sequences within each input file. Its compression ratio on 1024-genome collections (Table 1 in the main paper) was rather good, between MBGC and NAF, but the compression time was usually more than 10 times longer than 7z's, the slowest competitor in that experiment. memRGC hanged during the compression. MtGC (`https://github.com/haicy/MtGC`) is a stronger variant of HRCM, yet its decompression failed in our experiments. The improvement in compression ratio with respect to HRCM also comes at a price of slower compression. Regarding iDoComp, we experimented with *C. jejuni* (1024-genome collection). It could not process two of our genomes (a problem with header parsing), so we removed them from the input. The compression time was more than 100 times longer than from MBGC default (408.1 s vs 3.7 s) and the resulting archive was by a factor of 10 times larger. We found those results not satisfactory (in other words, iDoComp is inappropriate for this kind of data) and performed no more tests with it. We note that in experiments on 1024-genome collections DELIMINATE has been roughly comparable with BSC regarding its compression speed an ratio, however the latter showed close to an order of magnitute better decompression speed. Finally, we decided to present only the results of BSC.

GeCo3 does not handle N symbols in the DNA. Leon and MFCompress require some data preprocessing. They work with a single FASTA file, so the input files had to be merged (using the mumu.pl script mentioned earlier). The performance of these tools was unsatisfactory. GeCo3 achieved compression ratios roughly comparable with BSC and HRCM, but was much slower (below 1 MB/s). Leon was not able to decompress some datasets, due to a segmentation fault, was rather slow in compression and offered the compression ratio somewhat worse than DELIMINATE's. Finally, MFCompress was quite weak in the compression ratio and slow in the decompression.

4.3 MBGC parameters

Fig. 3 and Fig. 4 present the impact of varying five MBGC parameters (k, m, u, s, o) on the overall compression ratios and compression times, on four bacterial datasets; each dataset is limited to 1024 genomes. We present these parameters in the next paragraphs. In the subfigures, only one parameter is modified at a time, while the others have default values.

The parameter k, whose default value is 32, is the minimum match length. It must be not less than the length of a seed (which is 28 by default) in the hash table (HT). A too small value of k, which thus also implies short seeds, leads to many collisions (where new entries overwrite the old ones in HT), while a too large k may disallow some short matches, and the hashing itself is more costly (although this is hardly an issue, if k is within reasonable limits).

The parameter m, which stands for "skipMargin", determines the position in the suffix of the most recent match from which the matching procedure will restart (skipping the rest of the match). Its default value is $m = 16$. The rationale behind this idea is to increase opportunities of finding a better match than the recent one (or even several recent matches). Increasing the skip margin further does not necessarily improve the compression ratio, but it tends to slow down the performance (for maximum mode m is slightly increased to 24).

The parameter u, or rather its reciprocal, determines the "growth rate" of the REF string. Its default value, $u = 192$, means that if and only if the fraction of the symbols in a just processed contig which is not covered with matches exceeds 1/192, then the REF string is appended with the contig and its reverse complement. Smaller values of u imply fewer contigs to meet this criterion, which makes the resulting REF shorter (which, however, does not necessarily reduce the compression memory usage, as a shorter REF tends to produce more literals, stored in the memory to be compressed later).

The parameter s, with the default value of 16, stands for the sampling step over the reference string. Larger s means that fewer substrings from REF become seeds to be inserted in the hash table (HT), which in turn makes the overall HT update faster and also requiring less memory (which is however also related to the parameter o, described later). On the other hand, with larger values of s some tentative LZ-matches may be missed.

Finally, the parameter o, which stands for "referenceFactorBinaryOrder" in the source code, sets an upper bound on the REF length, as $2^o \times (|G_1| \cdot 2)$, where $|G_1|$ is the length (in bytes) of the first genome in the processed collection, and the factor 2 corresponds to storing both G_1 and its reverse complement. The value of o is set to the largest integer from $\{5, 6, \ldots, 12\}$ such that the number of input files (genomes) is not smaller than 2^{-16+3o}. For example, for a 1024-genome collection we have $o = 8$, and assuming that $|G_1|$ is exactly 5 MB, we obtain that REF is limited to 2560 MB in its length. This parameter also affects the HT size. We assume the number of its slots is at least 2^{24}, but in fact its number of slots is 2^j for such maximal j that $2^j < |REF_{max}|/s$, where REF_{max} is the just discussed limit of the REF length, s is the

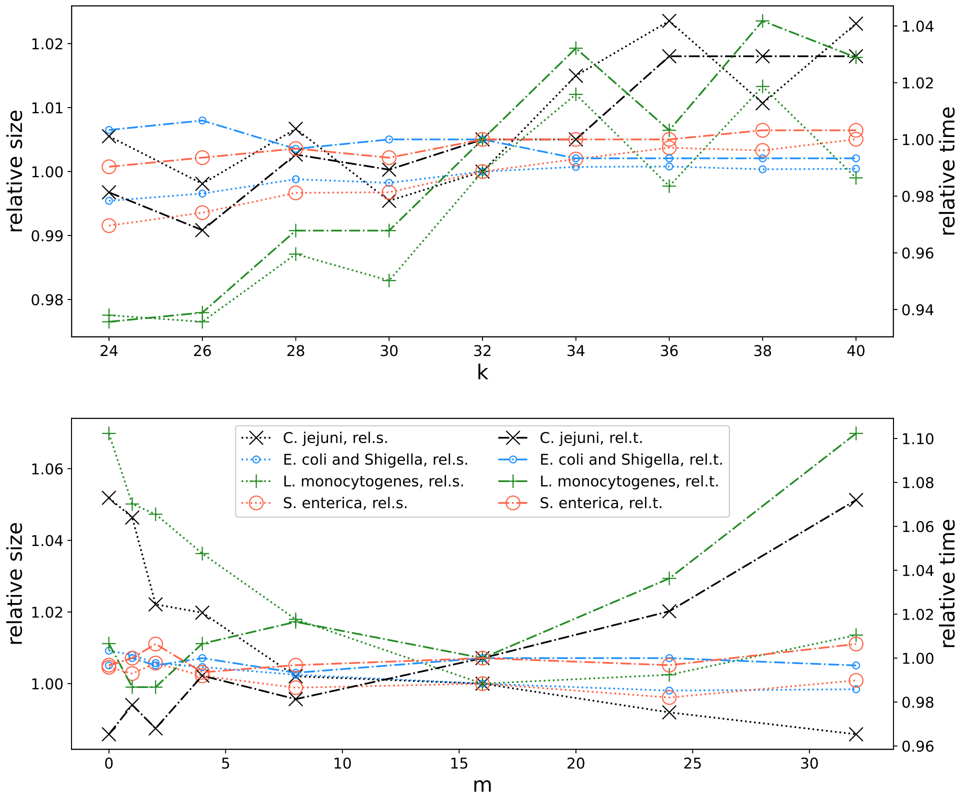

Figure 3: Relative compression ratios and times when one of the compression parameters is varied and the remaining parameters keep their default value. The left (resp. right) Y axes are related to relative compressed sizes (resp. compression times).

sampling step, and j is additionally upper-bounded by 31. The rationale for such a HT size is quite obvious, as we do not want to have more inserts to HT than its number of slots (as repeating seeds, for reasonable values of k and u, are not that frequent).

Let us now comment the results. The first observation is that we cannot speed up the default mode of MBGC more than just a little varying one of the presented parameters. On the other hand, we can it make much slower (by a factor 2 or more) in the compression by setting small values to s and/or o; a small value of o is a clearly bad choice. The impact of the parameter k on both the compression ratio and speed is fairly small in the whole presented range of values, from 24 to 40. The plot suggests that the smaller k values are better in terms of both speed and ratio, but actually this tendency is reversed for larger genome collections, therefore a value from the middle of the considered range should be favored overall. The compression is also fairly stable with varying u, if only it is not too small (note a compression loss exceeds 10% for the *L. monocytogenes* dataset when u is around 64 and below).

To facilitate understanding of MBGC's internals, e.g., when the reference sequence is prolonged or how the skip margin idea works, we made up an example (Fig. 5, showing the successive stages, from creating REF out of the first genome (G_1) to finding and left- and right-extending matches and possibly updating REF together with the hash table).

5 MBGC backend compression

In this short section, we explain how the by-products (streams) of our LZ77-like compression procedure are compressed in the last stage. For all of them the lzma library is used.

There are two essentially different algorithms handled by this library: LZMA (from the Lempel-Ziv'77 family) and PPMd (from the Prediction by Partial Matching family). PPMd makes use of two parameters: maximum memory usage (set to 192 MB in our application) and its maximum order. We use it to compress two (major) streams: of reference (i.e., G_1 only, without its reverse-complemented twin string) and literals, with order 7 and 2 blocks being compressed in parallel, and of match lengths, with order 6 and 3 blocks being compressed in parallel. The settings were found experimentally.

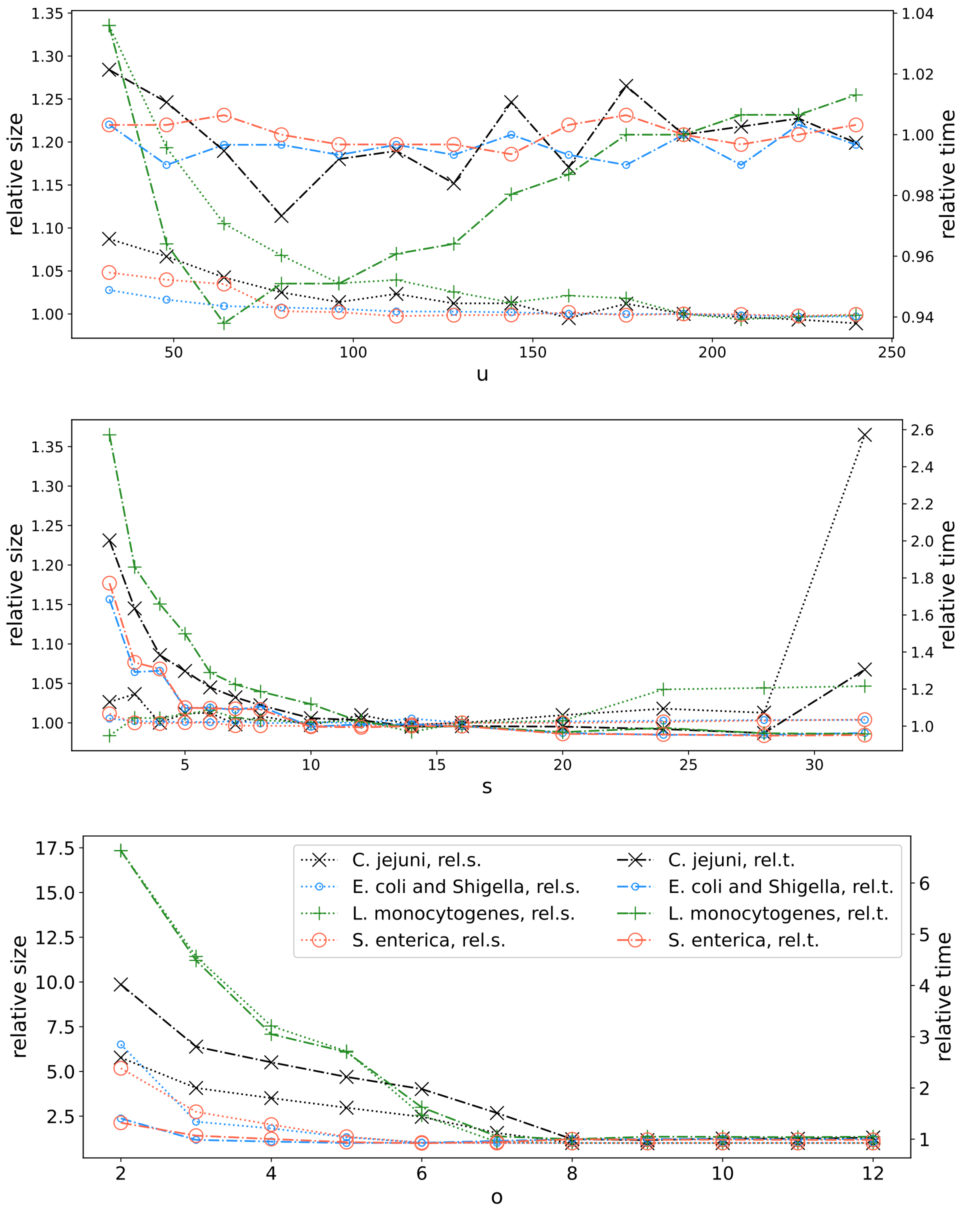

Figure 4: Relative compression ratios and times when one of the compression parameters is varied and the remaining parameters keep their default value. The left (resp. right) Y axes are related to relative compressed sizes (resp. compression times).

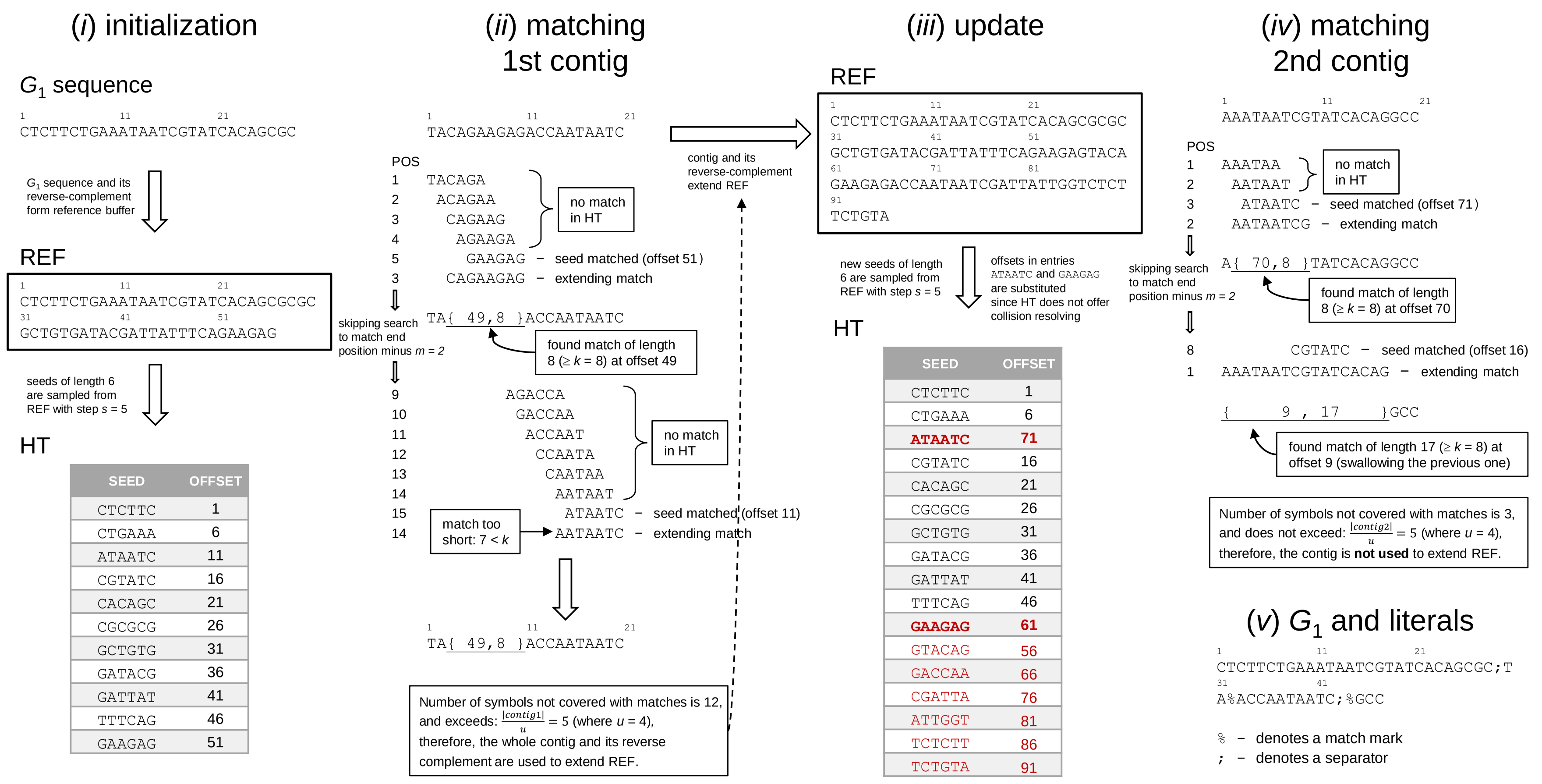

Figure 5: Steps of the MBGC compression, on a high level, for a toy example. The parameters used for this example are: seed length 6, $k = 8$, $m = 2$, $u = 4$, $s = 5$. (i) REF is created from the first genome, G_1, of length 28 (REF is now the concatenation of G_1 and $rc(G_1)$, and thus twice longer), and the hash table HT is populated with seeds taken from REF. (ii) Substrings from the first contig are sought in HT, and the found matches are left- and right-extended. Note how the skip margin idea is applied. After the contig is processed, the decision is made if REF should be extended (affirmative here). (iii) REF is appropriately extended and HT is updated; newer seeds overwrite older ones in case of a collision. The new seeds in HT are marked red, and additionally with bold typeface when overwriting. (iv) Processing another config. Note how the skip margin idea allows to swallow the previously found match (thus reducing the number of output matches). The decision if REF should be extended is negative this time. (v) The final stream of literals and matches, where matches are only marked with % and contig separators are marked with ;. The prefix of this stream of length 28 is the genome G_1.

Match lengths are 2-byte aligned, i.e., each length less than 2^{15} is stored on 2 bytes, but in rare cases of even a longer match the length is stored on 4 bytes.

LZMA is used to compress the stream of match offsets. We use it with its maximum compression mode, with extra parameters set to improve the compression on periodical data (namely, lp = 2, pb = 2, appropriate for 4-byte data items), and running 2 worker threads. Additionally, this LZMA stream is divided into 2 blocks compressed in parallel.

Note that the presented streams, which are the largest ones, are divided into 2 or 3 separate blocks, to increase the parallelism (different streams are also compressed in parallel). Such a partitioning into blocks is not used, however, for small inputs, as the minimum length of a block is set to 2^{20} bytes. There are a few other, minor, data streams (e.g., corresponding to sequence headers or file names) as well. Their impact on the overall compression (and other aspects, like compression speed) is rather negligible and for this reason we omit presenting details of their handling.
