# Supplementary material for: MBGC: Multiple Bacteria Genome Compressor
Source: Gigascience. 2022 Jan 27;11:giab099. doi: 10.1093/gigascience/giab099 (PMC8848312; doi:10.1093/gigascience/giab099)
Supplement: giab099_Response_to_Reviewer_Comments_Revision_2 [file giab099_response_to_reviewer_comments_revision_2.pdf]

Author's Response To Reviewer Comments

Close

Dear Editor,

We submit a final version of our manuscript:

"MBGC: Multiple Bacteria Genome Compressor"
by
Szymon Grabowski and Tomasz M. Kowalski.

The changes are:
* the GigaDB DOI is cited under "Availability of Supporting Data" and included in the References,
* references are all augmented with DOIs,
* bio.tools ID and SciCrunch.org RRID are now included in the "Availability of source code and requirements" section.

Additionally, all the color marking (red), previously introduced for Reviewers' convenience, is removed from the manuscript.

Yours sincerely,
Szymon Grabowski (on behalf of both co-authors)

Close
