# Supplementary material for: MBGC: Multiple Bacteria Genome Compressor
Source: Gigascience. 2022 Jan 27;11:giab099. doi: 10.1093/gigascience/giab099 (PMC8848312; doi:10.1093/gigascience/giab099)
Supplement: giab099_Reviewer_1_Report_Original_Submission — Diogo Pratas -- 7/27/2021 Reviewed [file giab099_reviewer_1_report_original_submission.pdf]

Reviewer Report

Title: MBGC: Multiple Bacteria Genome Compressor

Version: Original Submission **Date:** 7/27/2021

Reviewer name: Diogo Pratas

Reviewer Comments to Author:

This article presents a new compressor that uses both direct and reverse-complemented LZ-matches with multi-threaded and cache optimizations.
Generally, the reported results of this tool are exciting, and once confirmed, they have good applicability in the bioinformatics community.
However, I could not reproduce the results by lack of instructions, the benchmark is not representative of the state-of-the-art, and there are also several associated questions.
Below the comments are specified.
Regarding the experiments:
1. The experiments could not be reproduced. Unfortunately, the instructions and documentation are not clear (See below my tentatives).
2. The benchmarking is missing several well-known tools (for example, naf, geco3, Deliminate, MFCompress, Leon, ...). See, for example:
Kryukov, Kirill, et al. "Nucleotide Archival Format (NAF) enables efficient lossless reference-free compression of DNA sequences." Bioinformatics 35.19 (2019): 3826-3828.
Silva, Milton, et al. "Efficient DNA sequence compression with neural networks." GigaScience 9.11 (2020): giaa119.
Yao, Haichang, et al. "Parallel compression for large collections of genomes." Concurrency and Computation: Practice and Experience (2021): e6339.
To access more compressors, please see the following benchmark (that is already cited in the article):
https://academic.oup.com/gigascience/article/9/7/giaa072/5867695
Regarding the manuscript:
1. The State-of-the-art in genomic data compression (or at least in collections of genomes) is brief and does not offer a consistent and diverse description of the already developed tools.
2. "By the compression ratio we mean the ratio between the original input size and the compressed size. If, for example, the ratio improves from 1000 to 1500, e.g., due to changing some parameters of the compressor, we can say that the compression ratio improves 1.5 times (or by 50%)."
This sentence seems a little confusing (at least for me). Please, rephrase.
3. "The performance of the specialized genome compressor, HRCM [7], is only mediocre, and we refrained from running it on the whole collection, as the compression would take about a week.".
The purpose of a data compressor can be very different: to use in machines with lower RAM, for compression-based analysis, for long-term storage, research purposes, among others.
The qualification of HRCM without putting it into context seems to be depreciative.
Regarding the tool and documentation:

1. Although I have downloaded and compiled the tool, I had to dedicate some minutes to a "libdeflate" default version issue.

The majority of the bioinformatics community uses conda. In order to minimize installation issues for the users, please, provide a conda installation for the proposed tool. Also, the libdeflate can already be retrieved with conda. Then, with the instructions for the installation of mbgc, please, add this line to mbgc repository:

conda install -c bioconda libdeflate

Notice that this "conda" part is a suggestion that will facilitate the usage of mbgc by the bioinformatics community.

2. Running ./mbgc gives the output:

./mbgc: For compression expected 2 arguments after options (found 0)

try './mbgc -?' for more information

If the menu appears as default (no arguments besides the program's name), it will be much more helpful.

3. The program should have a version flag to depict the version of the program (besides the version at the menu). This feature is essential for integration/implementations (e.g., conda) and to differentiate from eventual new versions to the mbgc software.

4. Please, provide a running example at the help menu (with tiny existing sequences at the repository).

5. Is this characteristic of mbgc a strict property: "decompresses DNA streams 80 bases per line"?

This characteristic may create differences between original files and uncompressed files. Perhaps, having the possibility to have a custom line size would be a valuable feature, at least for data compression scientists to access and compare with other compressors, mainly because it makes the decompressor not completely lossless (although in practice, there is minimal information required to maintain the whole lossless property). Nevertheless, if the program decompresses FASTA data with a unique line size (for DNA bases) of 80 bases, this should also be mentioned in the article (besides what already exists in the repository).

6. The first impression was that "sequencesListFile" are the IDs of the bacterial genomes, then I found out that they are the URL-suffixes for the FASTA repository. Then, I start to wonder if mbgc could accept directly the FASTA containing the collection of genomes. How can the user provide the FASTA file directly? This feature would simplify a lot the usage of mbgc. Rationale: most of the reconstruction pipelines output multi-FASTA sequences in a single file. Therefore, this feature has direct applicability. Please, add more information about this in the help print and at the README. A higher goal would be to have stdin and stdout in compression/decompression as an option and the style of the argument as POSIX (Program Argument Syntax Conventions). This features are important for building bioinformatics pipelines and perform analysis (especially since the tools seems to be ultra-fast).

7. Table 1,2,3,4 (and the additional table at supplementary material) have "Compress / decompress times (as "ctime" / "dtime") are given in seconds," but no unity reference is provided in the cap to the cmemory and dmemory. Is this value on a GigaByte unity?

8. The README should provide a small example for testing purposes with the files already available at the repository or by efetch download (see below).

9. The reproducibility is hard to follow:

I had to search for the following procedure to test the software:

wget https://github.com/kowallus/mbgc/releases/download/v1.1/tested_samples_lists.7z
7z e tested_samples_lists.7z
After the cere download, also
tar -vzxf cere_assemblies.tgz
Then, I realized that it was missing the sequences, and by the NCBI interface, I lost track. I gave up after a few segmentation faults/combinations without understanding if the program or the settings generated the issue.
Please provide supplementary material and README with the complete instructions to reproduce the experiments (the exact commands).
Also, this simple way to download a multi-FASTA file with Escherichia Coli sequences may be helpful:
conda install -y -c conda-forge -c bioconda -c defaults entrez-direct
esearch -db nucleotide -query "Escherichia coli" | efetch -format fasta > Escherichia.mfa

Methods

Are the methods appropriate to the aims of the study, are they well described, and are necessary controls included? Choose an item.

Conclusions

Are the conclusions adequately supported by the data shown? Choose an item.

Reporting Standards

Does the manuscript adhere to the journal's guidelines on [minimum standards of reporting?]() Choose an item.

Choose an item.

Statistics

Are you able to assess all statistics in the manuscript, including the appropriateness of statistical tests used? Choose an item.

Quality of Written English

Please indicate the quality of language in the manuscript: Choose an item.

Declaration of Competing Interests

Please complete a declaration of competing interests, considering the following questions:

- Have you in the past five years received reimbursements, fees, funding, or salary from an organisation that may in any way gain or lose financially from the publication of this manuscript, either now or in the future?

- Do you hold any stocks or shares in an organisation that may in any way gain or lose financially from the publication of this manuscript, either now or in the future?
- Do you hold or are you currently applying for any patents relating to the content of the manuscript?
- Have you received reimbursements, fees, funding, or salary from an organization that holds or has applied for patents relating to the content of the manuscript?
- Do you have any other financial competing interests?
- Do you have any non-financial competing interests in relation to this paper?

If you can answer no to all of the above, write 'I declare that I have no competing interests' below. If your reply is yes to any, please give details below.

I declare that I have no competing interests.

I agree to the open peer review policy of the journal. I understand that my name will be included on my report to the authors and, if the manuscript is accepted for publication, my named report including any attachments I upload will be posted on the website along with the authors' responses. I agree for my report to be made available under an Open Access Creative Commons CC-BY license (http://creativecommons.org/licenses/by/4.0/). I understand that any comments which I do not wish to be included in my named report can be included as confidential comments to the editors, which will not be published.

Choose an item.

To further support our reviewers, we have joined with Publons, where you can gain additional credit to further highlight your hard work (see: https://publons.com/journal/530/gigascience). On publication of this paper, your review will be automatically added to Publons, you can then choose whether or not to claim your Publons credit. I understand this statement.

Yes Choose an item.
