# Supplementary material for: MBGC: Multiple Bacteria Genome Compressor
Source: Gigascience. 2022 Jan 27;11:giab099. doi: 10.1093/gigascience/giab099 (PMC8848312; doi:10.1093/gigascience/giab099)
Supplement: giab099_Reviewer_2_Report_Original_Submission — Jinyan Li -- 8/12/2021 Reviewed [file giab099_reviewer_2_report_original_submission.pdf]

Reviewer Report

Title: MBGC: Multiple Bacteria Genome Compressor

Version: Original Submission **Date:** 8/12/2021

Reviewer name: Jinyan Li

Reviewer Comments to Author:

This paper proposed a compression algorithm to compress sets of bacterial genome sequences. The motivation is based on the reason that the existing algorithms and tools are targeted at large, e.g., mammalian, genomes, and their performance on bacteria strains is unknown. The key idea of the proposed method is to detect characteristic features from both the direct and reverse-complemented copies of the reference genome via LZ-matching. The compression ratio is high and the compression speed is fast. Specifically, on a collection of 168,311 bacterial genomes (587 GB in file size), the algorithm achieved a compression ratio around the factor of 1260. The author claimed that the performance is much better than the existing algorithms. Overall, the quality of the paper is quite good.
I have two suggestions for the author to improve the manuscript:
1/ it's not clear to me about this sentence that "we focus on the compression of bacterial genomes, for which existing genome collection compressors are not appropriate from algorithmic or technical reasons." More clarifications are needed.
2/ With my own experience, GDC2 has a better performance on virus genome collections than HRCM. It's strongly suggested for the author to add the performance of GDC2 on the bacterial genome collections.

Methods

Are the methods appropriate to the aims of the study, are they well described, and are necessary controls included? Choose an item.

Conclusions

Are the conclusions adequately supported by the data shown? Choose an item.

Reporting Standards

Does the manuscript adhere to the journal's guidelines on minimum standards of reporting? Choose an item.

Choose an item.

Statistics

Are you able to assess all statistics in the manuscript, including the appropriateness of statistical tests used? Choose an item.

Quality of Written English

Please indicate the quality of language in the manuscript: Choose an item.

Declaration of Competing Interests

Please complete a declaration of competing interests, considering the following questions:

- Have you in the past five years received reimbursements, fees, funding, or salary from an organisation that may in any way gain or lose financially from the publication of this manuscript, either now or in the future?
- Do you hold any stocks or shares in an organisation that may in any way gain or lose financially from the publication of this manuscript, either now or in the future?
- Do you hold or are you currently applying for any patents relating to the content of the manuscript?
- Have you received reimbursements, fees, funding, or salary from an organization that holds or has applied for patents relating to the content of the manuscript?
- Do you have any other financial competing interests?
- Do you have any non-financial competing interests in relation to this paper?

If you can answer no to all of the above, write 'I declare that I have no competing interests' below. If your reply is yes to any, please give details below.

'I declare that I have no competing interests'

I agree to the open peer review policy of the journal. I understand that my name will be included on my report to the authors and, if the manuscript is accepted for publication, my named report including any attachments I upload will be posted on the website along with the authors' responses. I agree for my report to be made available under an Open Access Creative Commons CC-BY license (http://creativecommons.org/licenses/by/4.0/). I understand that any comments which I do not wish to be included in my named report can be included as confidential comments to the editors, which will not be published.

Choose an item.

To further support our reviewers, we have joined with Publons, where you can gain additional credit to further highlight your hard work (see: https://publons.com/journal/530/gigascience). On publication of this paper, your review will be automatically added to Publons, you can then choose whether or not to claim your Publons credit. I understand this statement.

Yes Choose an item.
