# Supplementary material for: MBGC: Multiple Bacteria Genome Compressor
Source: Gigascience. 2022 Jan 27;11:giab099. doi: 10.1093/gigascience/giab099 (PMC8848312; doi:10.1093/gigascience/giab099)
Supplement: giab099_Reviewer_3_Report_Original_Submission — idoia ochoa -- 8/13/2021 Reviewed [file giab099_reviewer_3_report_original_submission.pdf]

Reviewer Report

Title: MBGC: Multiple Bacteria Genome Compressor

Version: Original Submission **Date:** 8/13/2021

Reviewer name: idoia ochoa

Reviewer Comments to Author:

The authors present a novel tool for the compression of collections of bacterial genomes. The authors present sound results that demonstrate the performance gain of their tool, MBGC, with respect to the state-of-the-art. As such, I do not have concerns about the method itself. My main concerns are with respect the description of the tool, and how the results are presented. Next I list some of my suggestions (in no particular order):

Main Paper:

- Analysis section: Before naming MBGC specify that it is the proposed tool.
- Analysis section: Reference for HRCM. Mention here also that other tools such as iDoComp, GDC2, etc. are discussed in the Supplementary (this way the reader knows more tools were analyzed or at least tried on the data).
- Analysis section: The paragraph "Our experiments with MBGC show that... " is a little misleading, since it seems that the tool has the capacity to compress a collection and just extract a single genome from it. This becomes clear later in the text when it is discussed how the tool could be used to speed up the download of a collection of genomes from a repository. So maybe explain that in more detail here, or mention that it could be used to compress a bunch of genomes prior to download. And then point to the part of the text where this is discussed in more detail.
- Analysis section: The results talk about the "stronger MGBC mode", the "MGBC max", but in the tables it reads "MBGC default" or "MBGC -c 3". I assume "MBGC -c 3" refers to "MBGC max", but it is not stated anywhere. maybe better to call it "MBGC default" and "MBGC max".
- Analysis section: Although the method is explained later in the text, it would be a good idea to give a sense of the difference between the default and max modes of the tool. Or some hints on the trade-off between the two. Also, the parameter "-c 3" is never explained.
- Analysis section: Figures, it is difficult to see the trade-off between relative size and relative time, can you use colored lines? such that the same color refers to the same set of genomes. Also, in the caption, explain if we want small or high relative size and time. it may be clear, but better to clearly state it.
- Analysis section: there is a sentence that says "all figures w.r.t. the default mode of MBCG". It would be good also to state that in the caption, so that the reader knows which mode of the tool is being used to generate the presented results. and if the input files are gzipped or not. For example, for the following paragraph that starts with Fig. 1, it is not clear if the files are gzipped or not.
- Analysis section: First time GDC2 is mentioned, the first thing that comes to mind is why it was not used for the bacterial experiments. See my previous point on having a couple of sentences about the other tools that were considered, and why they are not included in the main tables/figures.
- Methods:

-- Here I am really missing a diagram explaining the main steps of the tool. It seems the paper has been rewritten slightly to fit the format of the journal and some things are not in the correct order. For example, it says the key ideas are already sketched, but i do not think that is true.
-- (offset, length) i assume refers to the position of the REF where the match begins, and the length of the match, but again, not really explained. A diagram would help. Also, when it is time to compress the pairs, are the offset delta encoded? or encoded as they are with a general compressor?
-- How are the produced tokens (offset, length, literals, etc.) finally encoded?
-- First time parameter "k" is mention, default value? Also, how can you do a left extension and "swallow" the previous match? is it because the previous match could have been at another position? otherwise if it was in that position it would have been already extended to the right, correct? i mean, it would have generated a longer match.
-- The "skip margin" idea is not well explained. not sure why the next position after a match is decreased by m. please explain better or use a diagram with an example.
-- when you mention 1/192, maybe already state that this is controlled by the parameter u. otherwise when you mention the different parameters is difficult to relate them to the explanation of the algorithm.
Availability of supp...
-- from from (typo)
Tables
-- Specify the number of genomes in each collection.
-- change MBGC -c 3 to MBGC max or something similar. (see my previous comment -c flag is not explained!)
Supplementary Material
-- move table 1 after the text for ease of reading
-- not clcear if the tool has random access or not. it is discussed the percentage of time (w.r.t. decompreessing the whole collection i believe) that it would take to decompress one of the first gneomes vs one of the last ones. this should be better explained. for example, if we decompress the last genome of the collection we will employ 100% of the time, right? given that previous genomes are part of REF (potentially). please explain better and discuss this point in the analysis part, not only in the supplementary. seems like an important aspect of the algorithm.
-- I assume this is not possible, but should be discussed as well. can you add a genome to an already compressed collection? this together with the random access capabilities will highlight better the main possible uses of the tool.
-- section 4.3: here HT is used, and then HT is introduced in the next paragraph. please revise the whole text and make sure everything is in the right order.
-- parameter m, please explain better.
-- add colors to figures, it will be easier to read them.
Overall, as I mentioned before, I believe the tool offers significant improvements with respect to the competitors for bacterial genomes, and performs well on non bacterial genomes as well. What should be improved for publication is the description of the method, since at the end of the day is the main contribution, and how the text is presented.

Methods

Are the methods appropriate to the aims of the study, are they well described, and are necessary controls included? Choose an item.

Conclusions

Are the conclusions adequately supported by the data shown? Choose an item.

Reporting Standards

Does the manuscript adhere to the journal's guidelines on minimum standards of reporting? Choose an item.

Choose an item.

Statistics

Are you able to assess all statistics in the manuscript, including the appropriateness of statistical tests used? Choose an item.

Quality of Written English

Please indicate the quality of language in the manuscript: Choose an item.

Declaration of Competing Interests

Please complete a declaration of competing interests, considering the following questions:

- Have you in the past five years received reimbursements, fees, funding, or salary from an organisation that may in any way gain or lose financially from the publication of this manuscript, either now or in the future?
- Do you hold any stocks or shares in an organisation that may in any way gain or lose financially from the publication of this manuscript, either now or in the future?
- Do you hold or are you currently applying for any patents relating to the content of the manuscript?
- Have you received reimbursements, fees, funding, or salary from an organization that holds or has applied for patents relating to the content of the manuscript?
- Do you have any other financial competing interests?
- Do you have any non-financial competing interests in relation to this paper?

If you can answer no to all of the above, write 'I declare that I have no competing interests' below. If your reply is yes to any, please give details below.

I declare that I have no competing interests

I agree to the open peer review policy of the journal. I understand that my name will be included on my report to the authors and, if the manuscript is accepted for publication, my named report including any attachments I upload will be posted on the website along with the authors' responses. I agree for my report to be made available under an Open Access Creative Commons CC-BY license (http://creativecommons.org/licenses/by/4.0/). I understand that any comments which I do not wish to be included in my named report can be included as confidential comments to the editors, which will not be published.

Choose an item.

To further support our reviewers, we have joined with Publons, where you can gain additional credit to further highlight your hard work (see: https://publons.com/journal/530/gigascience). On publication of this paper, your review will be automatically added to Publons, you can then choose whether or not to claim your Publons credit. I understand this statement.

Yes Choose an item.
